# Defects in microvillus crosslinking sensitize to colitis and inflammatory bowel disease

**DOI:** 10.15252/embr.202357084

**Published:** 2023-09-11

**Authors:** Bernadette Mödl, Monira Awad, Daniela Zwolanek, Irene Scharf, Katharina Schwertner, Danijela Milovanovic, Doris Moser, Katy Schmidt, Petra Pjevac, Bela Hausmann, Dana Krauß, Thomas Mohr, Jasmin Svinka, Lukas Kenner, Emilio Casanova, Gerald Timelthaler, Maria Sibilia, Sigurd Krieger, Robert Eferl

**Affiliations:** ^1^ Center for Cancer Research Medical University of Vienna & Comprehensive Cancer Center (CCC) Vienna Austria; ^2^ Department of Experimental and Translational Pathology, Institute of Clinical Pathology Medical University of Vienna Vienna Austria; ^3^ Department of Cranio‐Maxillofacial and Oral Surgery Medical University of Vienna Vienna Austria; ^4^ Cell Imaging & Ultrastructure Research University of Vienna Vienna Austria; ^5^ Joint Microbiome Facility of the Medical University of Vienna and the University of Vienna Vienna Austria; ^6^ Division of Microbial Ecology, Department of Microbiology and Ecosystem Science, Centre for Microbiology and Environmental Systems Science University of Vienna Vienna Austria; ^7^ Department of Laboratory Medicine Medical University of Vienna Vienna Austria; ^8^ Department of Analytical Chemistry University of Vienna Vienna Austria; ^9^ Joint Metabolome Facility University of Vienna and Medical University Vienna Vienna Austria; ^10^ Department of Laboratory Animal Pathology University of Veterinary Medicine Vienna Vienna Austria; ^11^ Center of Physiology and Pharmacology, Institute of Pharmacology Medical University of Vienna & Comprehensive Cancer Center (CCC) Vienna Austria

**Keywords:** brush border, CDHR5, colitis, microvilli, MUCDHL, Cell Adhesion, Polarity & Cytoskeleton, Immunology, Microbiology, Virology & Host Pathogen Interaction

## Abstract

Intestinal epithelial cells are covered by the brush border, which consists of densely packed microvilli. The Intermicrovillar Adhesion Complex (IMAC) links the microvilli and is required for proper brush border organization. Whether microvillus crosslinking is involved in the intestinal barrier function or colitis is currently unknown. We investigate the role of microvillus crosslinking in colitis in mice with deletion of the IMAC component CDHR5. Electron microscopy shows pronounced brush border defects in CDHR5‐deficient mice. The defects result in severe mucosal damage after exposure to the colitis‐inducing agent DSS. DSS increases the permeability of the mucus layer and brings bacteria in direct contact with the disorganized brush border of CDHR5‐deficient mice. This correlates with bacterial invasion into the epithelial cell layer which precedes epithelial apoptosis and inflammation. Single‐cell RNA sequencing data of patients with ulcerative colitis reveals downregulation of CDHR5 in enterocytes of diseased areas. Our results provide experimental evidence that a combination of microvillus crosslinking defects with increased permeability of the mucus layer sensitizes to inflammatory bowel disease.

## Graphical abstract

Crosslinking of microvilli in the intestinal brush border mediated by CDHR5 represents an important barrier against invasion of luminal bacteria. A combination of reduced crosslinking with increased mucus layer permeability may sensitize to inflammatory bowel disease.

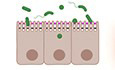

## Introduction

The microvilli in the brush border of intestinal epithelial cells significantly increase the surface area of the gut epithelial cells, providing more space for membrane proteins involved in nutrient uptake or defense against pathogens (Maroux *et al*, 1988; Koyama *et al*, 2002; Helander & Fandriks, 2014). Microvilli appear early in enterocyte differentiation. They form initial clusters that fuse into larger structures. Finally, they completely cover the entire surface of differentiated enterocytes (Crawley *et al*, 2014a). The formation of microvilli requires a pushing force provided by polymerization of a core actin bundle that deforms the cell membrane (Sheetz, 2001; Ohta *et al*, 2012). Individual actin filaments in the core actin bundle are connected by actin‐bundling proteins villin, espin, EPS5, and fimbrin (Crawley *et al*, 2014a). Fimbrin also anchors the roots of the core actin bundle to the terminal web, a contractile structure located beneath the inner lipid layer of the enterocyte apical membrane (Grimm‐Gunter *et al*, 2009). In addition, several proteins of the ERM (ezrin, radixin, moesin) and myosin families, such as ezrin, MYO1A, and MYO6 connect the actin core to the lipid bilayers of microvilli (Crawley *et al*, 2014a). Without these proteins, the membrane extrusions would go to their lowest energy state, leading to membrane coalescence and microvillus fusion (Saotome *et al*, 2004; Tyska *et al*, 2005; Hegan *et al*, 2012).

The microvilli are crosslinked by the intermicrovillar adhesion complex (IMAC), which is located at the tips of the microvilli and ensures their regular spatial packing (Crawley *et al*, 2014a, 2014b). The transmembrane proteins CDHR2 and CDHR5 belong to the protocadherin family and are part of the IMAC. Like classical cadherins, protocadherins are involved in cell adhesion but may also have additional functions in cell signaling (Kim *et al*, 2011). In the IMAC, the extracellular domains of CDHR2 and CDHR5 mediate microvillus crosslinking while their intracellular domains are connected to the cytoplasmic proteins USH1C, MYO7B, ANKS4B, and CALML4, which are anchored in the core actin bundle (Crawley *et al*, 2014a, 2014b, 2016; Choi *et al*, 2020). Deletion of CDHR2 in mice and in CACO‐2 BBe cells, which form a brush border at confluency, has revealed structural and biochemical consequences of IMAC ablation on microvillus organization (Crawley *et al*, 2014b; Pinette *et al*, 2019). The studies showed that ablation of CDHR2 resulted in reduced packing density of microvilli, shortening of microvilli, and reduced protein expression of several apical marker proteins such as intestinal alkaline phosphatase. In addition, microvillus tip localization of IMAC components CDHR5, USH1C, and MYO7B was lost (Pinette *et al*, 2019). Recently, a second IMAC, composed of the proteins TMIGD1, SLC9A3R1, and SLC9A3R2, was discovered at the base of microvilli (Hartmann *et al*, 2022). Deletion of TMIGD1 resulted in similar brush border defects as deletion of CDHR2 (Pinette *et al*, 2019; Hartmann *et al*, 2022). Whether these structural and biochemical aberrations have pathologic consequences is not known.

Crohn's disease (CD) and ulcerative colitis (UC) are chronic inflammatory conditions of the gut commonly referred to as inflammatory bowel disease (IBD). IBD is particularly common in developed countries such as Europe and North America where > 0.3% of residents are affected. However, the incidence rate and the corresponding healthcare costs are also increasing in developing countries (Ng *et al*, 2018). It is believed that IBD results from a complex interplay of environmental and genetic factors, dysregulated immunity, dysbiosis, and epithelial defects. This opinion is supported by the previous identification of multiple IBD risk genes involved in stress pathways, microbial recognition, cytokine signaling, inflammation, and mucosal barrier function (Graham & Xavier, 2020). However, it is not clear which of these factors or combinations of factors are the main cause of IBD or just a consequence of it. Previous studies have mainly focused on immunological mechanisms of IBD development, but more recently, evidence for a significant contribution of the intestinal epithelium was provided (Martini *et al*, 2017). A brush border‐related gene expression signature was identified in almost all tissue samples from patients with CD. The disease score of the affected areas as well as the endoscopy score of patients with CD in the UNITI‐2 study correlated with reduced expression of this signature. Furthermore, diseased gut areas of patients with CD showed microvillus fanning while brush border organization was preserved in non‐diseased areas (VanDussen *et al*, 2018). Here, we provide experimental evidence that microvillus crosslinking defects sensitize to colitis when the mucus layer is leaky and bacteria reach the apical surface of intestinal epithelial cells.

## Results

### Brush border defects in CDHR5 ^∆/∆^ mice

**Figure 1:**
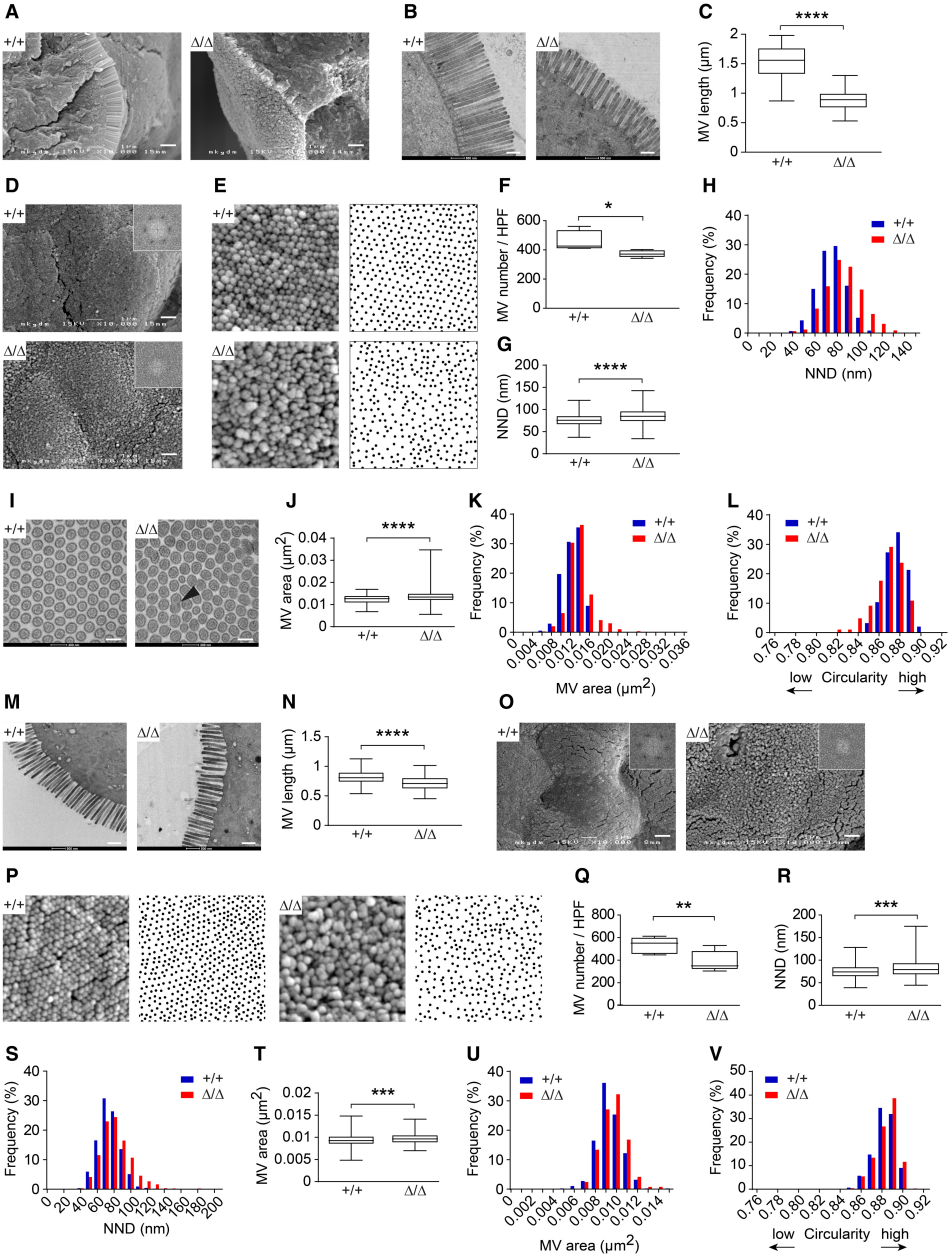
Microvillus length and packing density is decreased in CDHR5^∆/∆^ mice ARepresentative SEM images (side view) of microvilli in the duodenum of CDHR5^+/+^ and CDHR5^∆/∆^ mice. Scale bar = 1 μm.BRepresentative TEM images showing longitudinal sections through microvilli. Scale bar = 500 nm.CBox plots with quantitative values of microvillus length.DRepresentative SEM images (top view) showing the brush border in the duodenum (insets represent Fast Fourier Transformation FFT frequency domain images of a representative image section). Scale bar = 1 μm.EHigh‐magnification SEM images (top view) of the duodenal brush border (left) and corresponding graphs with black spots (right) representing positions of microvillus tips in SEM images.F–HGraphs with black spots (as shown in E) were used for quantitation of microvillus numbers (F), nearest neighbor distances of microvilli (G) and frequency distributions of the nearest neighbor distances (H). HPF, high‐power field.ITEM images showing microvillus cross‐sections. The arrowhead indicates fused microvilli. Scale bar = 200 nm.J–LImages of microvillus cross‐sections (as shown in I) were used for quantitation of microvillus areas (J), frequency distributions of the microvillus areas (K) and calculation of microvillus circularity (L).MTEM images showing longitudinal sections through microvilli of CDHR5^+/+^ and CDHR5^∆/∆^ colonocytes. Scale bar = 500 nm.NBox plots with quantitative values of microvillus length.OSEM images (top view) showing the brush border in the colon (insets represent Fast Fourier Transformation FFT frequency domain images of a representative image section). Scale bar = 1 μm.PHigh‐magnification SEM images (top view) of the colonic brush border (left) and corresponding graphs with black spots (right) representing positions of microvillus tips in SEM images.Q–SGraphs with black spots (as shown in P) were used for quantitation of colonic microvillus numbers (Q), nearest neighbor distances of microvilli (R) and frequency distributions of the nearest neighbor distances (S). HPF, high‐power field.T–VTEM images of colonic microvillus cross‐sections were used for quantitation of microvillus areas (T), frequency distributions of the microvillus areas (U) and for calculation of microvillus circularity (V). Data information: For (C) three mice per genotype and 45 microvilli per mouse were analyzed. For (F) five mice per genotype and one representative SEM image per mouse were analyzed. For (G and H) SEM images of three mice per genotype with > 340 microvilli per image were analyzed. For (J–L) TEM images of three mice per genotype with > 80 microvilli per image were analyzed. For (N) five mice per genotype and 50 microvilli per mouse were analyzed. For (Q) three mice per genotype and two representative SEM images per mouse were analyzed. For (R and S) SEM images of three mice per genotype with > 320 microvilli per image were analyzed. For (T–V) TEM images of three mice per genotype with > 80 microvilli per image were analyzed. Statistical analysis of all box plots was performed using unpaired Student's *t*‐test. *P < 0.05, **P < 0.01, ***P < 0.001 or ****P < 0.0001. Source data are available online for this figure.

**Figure EV1:**
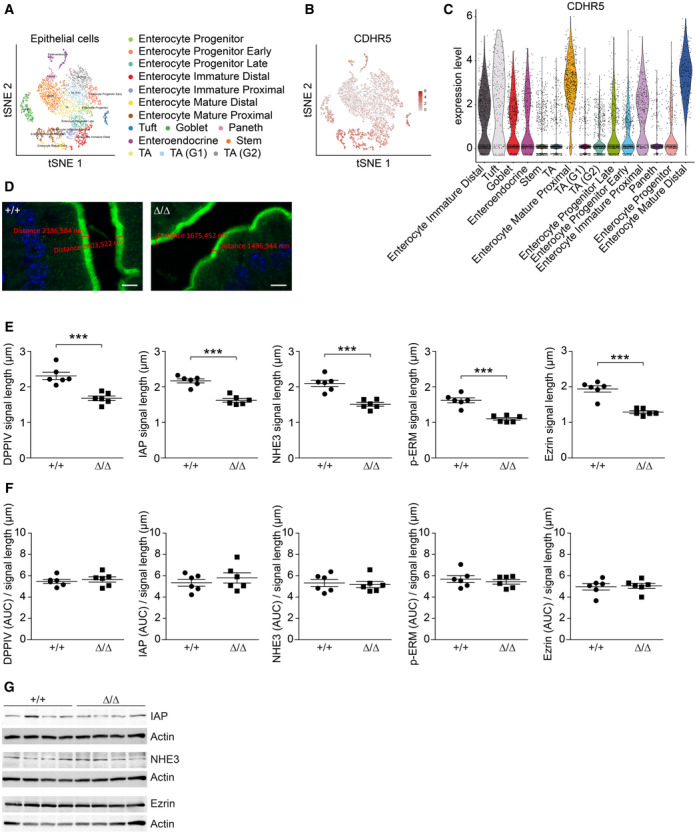
CDHR5 is mainly expressed by differentiated intestinal epithelial cells tSNE plot of adult mouse intestinal epithelial cells.tSNE plot of adult mouse intestinal epithelial cells indicating the expression of CDHR5.Violin plots for expression of CDHR5 in individual adult mouse intestinal epithelial cell lineages. For (A–C) the scRNA‐seq dataset of Haber was used (Haber *et al*, 2017).High‐magnification immunofluorescence images of duodenal epithelial cells stained for IAP. The length of the fluorescence signal (green) in the brush border was measured with the ZEN software. Two measurements per genotype are indicated. Note the reduced length of the fluorescence signal in CDHR5^∆/∆^ mice. Scale bar = 5 μm.Scatter plots showing the length of fluorescence signals for DPPIV, IAP, NHE3, p‐ERM and ezrin on fluorescent images of duodenal epithelial cells from CDHR5^+/+^ and CDHR5^Δ/Δ^ mice (see Fig 2E). Length measurements were performed with ZEN software as shown in (D).Normalization of the area under the curve values (see Fig 2F) to the reduced length of the fluorescence signals eliminated the differences in relative protein levels of DPPIV, IAP, NHE3, p‐ERM, and ezrin between CDHR5^+/+^ and CDHR5^Δ/Δ^ mice.Western blot for apical markers IAP, NHE3, and ezrin. Protein was isolated from purified intestinal epithelial cells of CDHR5^+/+^ and CDHR5^∆/∆^ mice. Actin was used as loading control. Data information: Scatter plots in (E and F) represent mean ± SEM (3 mice per genotype and 2 duodenal regions per mouse were analyzed). Each data point in (E) represents the mean of 5 length measurements per region. Statistical analyses were performed using unpaired Student's *t*‐test. Differences in (F) are not significant. ***P < 0.001.

We generated CDHR5 knock‐out (CDHR5^∆/∆^) mice (Appendix Fig S1A and B) to investigate CDHR5 functions in the intestinal brush border. CDHR5^∆/∆^ mice were born at Mendelian ratios, developed normally, and were fertile. The body weight was not changed in adult mice (Appendix Fig S1C). Immunofluorescence revealed pronounced CDHR5 protein expression in the brush border of intestinal epithelial cells in CDHR5^+/+^ mice, reduced expression in heterozygous CDHR5^+/∆^ mice and no expression in CDHR5^∆/∆^ mice (Appendix Fig S1D). qPCR from purified intestinal epithelial cells confirmed these data at the mRNA level (Appendix Fig S1E). We used a published single‐cell RNA sequencing (scRNA‐seq) dataset of murine intestinal epithelial cells to examine intestinal cell types expressing CDHR5. Expression was low in stem cells, transit‐amplifying cells, and Paneth cells whereas other cell types show higher expression levels (Fig EV1A–C). Loss of CDHR5 did not affect proliferation, apoptosis, and differentiation of intestinal cell types (Appendix Fig S2A–L). Gut regions with voids lacking villi were found in the small intestine of CDHR2‐deficient mice (Pinette *et al*, 2019). In CDHR5^∆/∆^ mice villus morphology was not affected (Appendix Fig S3A–E) and scanning electron microscopy (SEM) revealed no gut regions with voids (Appendix Fig S3F). However, a significant disorganization of the brush border was observed. SEM and transmission electron microscopy (TEM) showed microvillus disorganization and shortening in the small intestine (Fig 1A–C). SEM micrographs and Fourier transformation of images revealed loss of hexagonal organization (Fig 1D and Appendix Fig S3G) as well as reduced microvilli numbers and a corresponding increase of nearest neighbor distances (Fig 1E–H). TEM images of microvillus cross‐sections revealed thicker microvilli with reduced circularity (Fig 1I–L) and some of the microvilli appeared to be fused (Fig 1I). A similar brush border disorganization with microvillus shortening was observed in the colon, except for microvillus circularity, which was not affected (Fig 1M–V). In summary, our results show that CDHR5 is essential for a uniform microvillus length and formation of tightly packed hexagonal arrangements of microvilli in the brush border of enterocytes.

### Loss of microvillus IMAC tip localization in CDHR5 ^∆/∆^ mice

**Figure 2:**
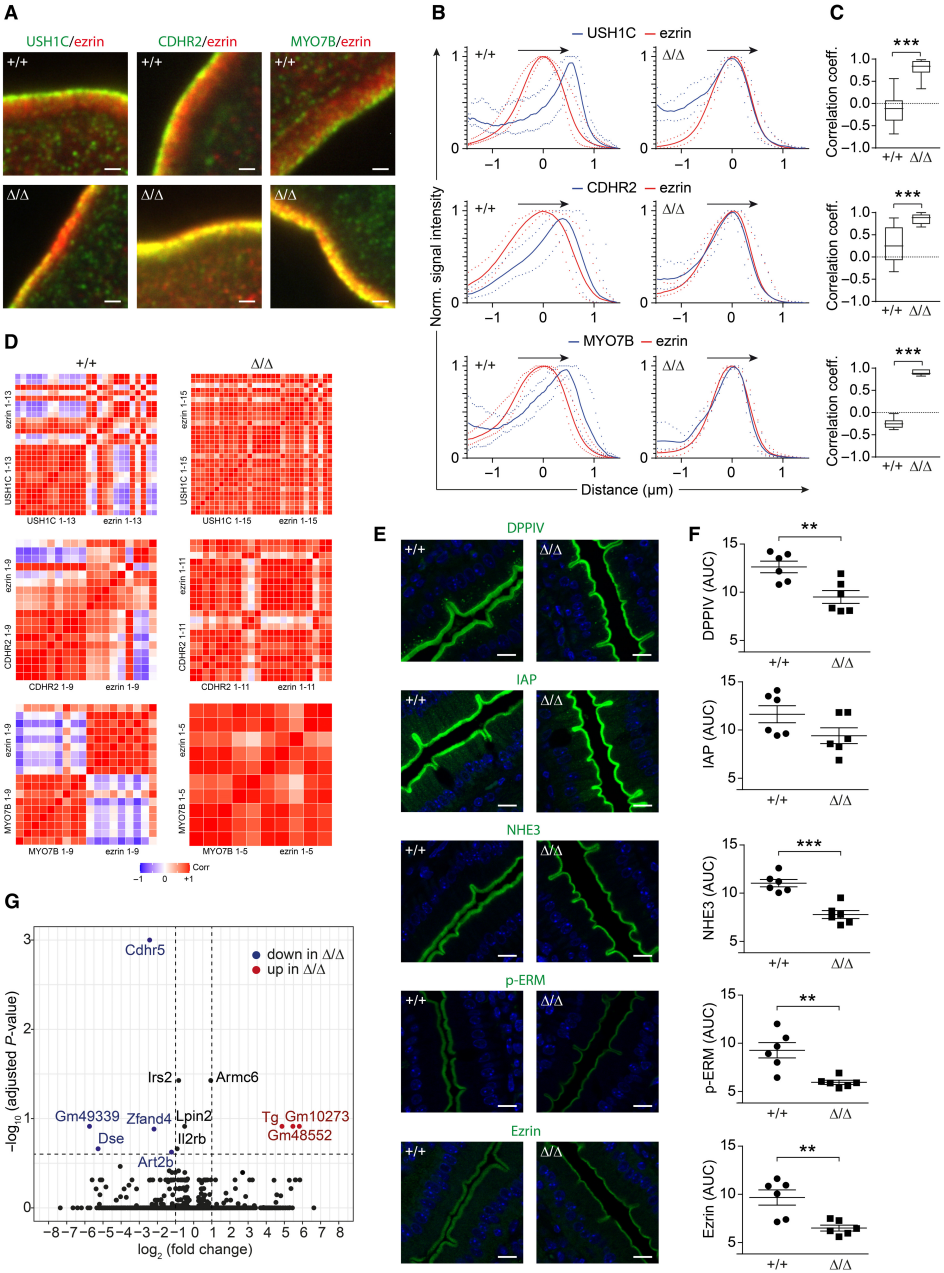
Loss of IMAC tip localization in CDHR5^∆/∆^ mice ASpinning disc immunofluorescence images of duodenal brush borders of CDHR5^+/+^ and CDHR5^∆/∆^ mice stained for USH1C/ezrin, CDHR2/ezrin or MYO7B/ezrin. USH1C, CDHR2 and MYO7B in green, ezrin in red, nuclei in blue. Scale bars = 1 μm. The images represent details of the images shown in Appendix Fig S4 at a lower magnification.BProfile plots showing the distribution of USH1C, CDHR2 and MYO7B relative to ezrin (which is distributed across whole microvilli) in the brush border (3 mice per genotype). The plots show normalized fluorescence intensities over the indicated line scans and have been centered on the maximum ezrin signal. Solid lines displaying mean signals and dotted lines represent the signal range, minimal and maximal signals.CColocalization of IMAC components with ezrin was assessed by a non‐parametric Spearmen test. The Spearman correlation coefficients are indicated by box plots.DCorrelation matrices showing colocalization of ezrin with IMAC components in CDHR5^∆/∆^ mice. The number of line scans used for the matrices are indicated.ELaser scanning immunofluorescence images of duodenal epithelial cells stained for DPPIV, IAP, NHE3, P‐Ezrin, and Ezrin. Scale bars = 10 μm.FScatter plots for quantitation of relative protein amounts using immunofluorescence images as shown in (E) (ImageJ software was used to calculate the area under the curve of corresponding profile plots).GVulcano plot of RNA sequencing data showing differentially expressed genes in purified intestinal epithelial cells of CDHR5^+/+^ and CDHR5^∆/∆^ mice (4 mice per genotype). Significantly downregulated and upregulated genes in intestinal epithelial cells of CDHR5^∆/∆^ mice are shown in blue and red colors, respectively. Data information: For (C) Spearman correlation coefficients of three mice per genotype were determined (USH1C: 65 for CDHR5^+/+^ and 33 for CDHR5^∆/∆^ mice, CDHR2: 17 for CDHR5^+/+^ and 24 for CDHR5^∆/∆^ mice, MYO7B: 9 for CDHR5^+/+^ and 5 for CDHR5^∆/∆^ mice). The Mann–Whitney test was used for assessment of significance. Scatter plots in (F) represent mean ± SEM (three mice per genotype and two duodenal regions per mouse were analyzed). Each data point in (E) represents the mean of 5 AUC measurements per region. Statistical analysis was performed using unpaired Student's *t*‐test. **P < 0.01, ***P < 0.001. Source data are available online for this figure.

It was shown in CDHR2‐deficient mice that tip localization of IMAC components was lost and that the amount of apical markers DPPIV, IAP, NHE3, p‐ERM, and ezrin was reduced (Pinette *et al*, 2019). Likewise, IMAC components were not properly localized in microvillus tips of CDHR5^∆/∆^ mice (Fig 2A–D, lower magnification immunofluorescence images in Appendix Fig S4) and the amount of apical markers, measured as area under the curve (AUC), was reduced (Fig 2E and F). However, the length of the fluorescence signals for apical markers was also shortened in CDHR5^Δ/Δ^ mice (Fig EV1D and E). The extent of the signal shortening correlated with the microvillus shortening determined by TEM (Fig 1C) and is therefore most likely due to the reduced microvillus length. When the AUC values were normalized to the reduced length of the fluorescence signals, the differences in relative protein levels for DPPIV, IAP, NHE3, p‐ERM, and ezrin between CDHR5^+/+^ and CDHR5^Δ/Δ^ mice were eliminated (Fig EV1F). This indicates that overall protein expression of DPPIV, IAP, NHE3, P‐ezrin, and ezrin is not altered in CDHR5^Δ/Δ^ mice, but the shortened microvilli provide less space for these proteins, resulting in decreased AUC values. To support this argument, we performed Western blot experiments for three apical markers IAP, NHE3, and ezrin, which showed comparable protein levels in purified epithelial cells of CDHR5^+/+^ and CDHR5^∆/∆^ mice (Fig EV1G). Several protocadherins interact with intracellular signaling molecules and may influence gene expression (Kim *et al*, 2011). Therefore, we performed RNA sequencing with intestinal epithelial cells of CDHR5^+/+^ and CDHR5^∆/∆^ mice. The RNA integrity number (RIN) of RNA isolated from epithelial preparations was very high, indicating preserved cell viability (Appendix Fig S5A). The purity of isolated epithelial cells was confirmed by RNA expression analysis for epithelial (CDH1, CDHR5) and lamina propria (FN1, PDGFRA, ACTA2) markers (Appendix Fig S5B and C). The RNA‐seq data revealed no major change in gene expression of epithelial cells upon loss of CDHR5 with only a few candidates found deregulated (Fig 2G). These data suggest that under physiological conditions CDHR5 has mainly structural functions in microvillus organization.

**Figure EV2:**
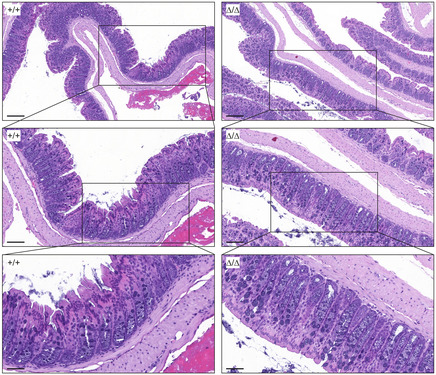
CDHR5^∆/∆^ mice do not develop spontaneous colitis Swiss rolls of colon tissue from CDHR5^+/+^ and CDHR5^Δ/Δ^ mice were stained with H&E. Images with increasing magnification (top to bottom) showed no evidence of spontaneous colitis or immune infiltration in CDHR5^Δ/Δ^ mice. Scale bars = 200, 100, and 50 μm from top to bottom images, respectively.

### Increased sensitivity of CDHR5 ^∆/∆^ mice to DSS‐induced colitis

**Figure 3:**
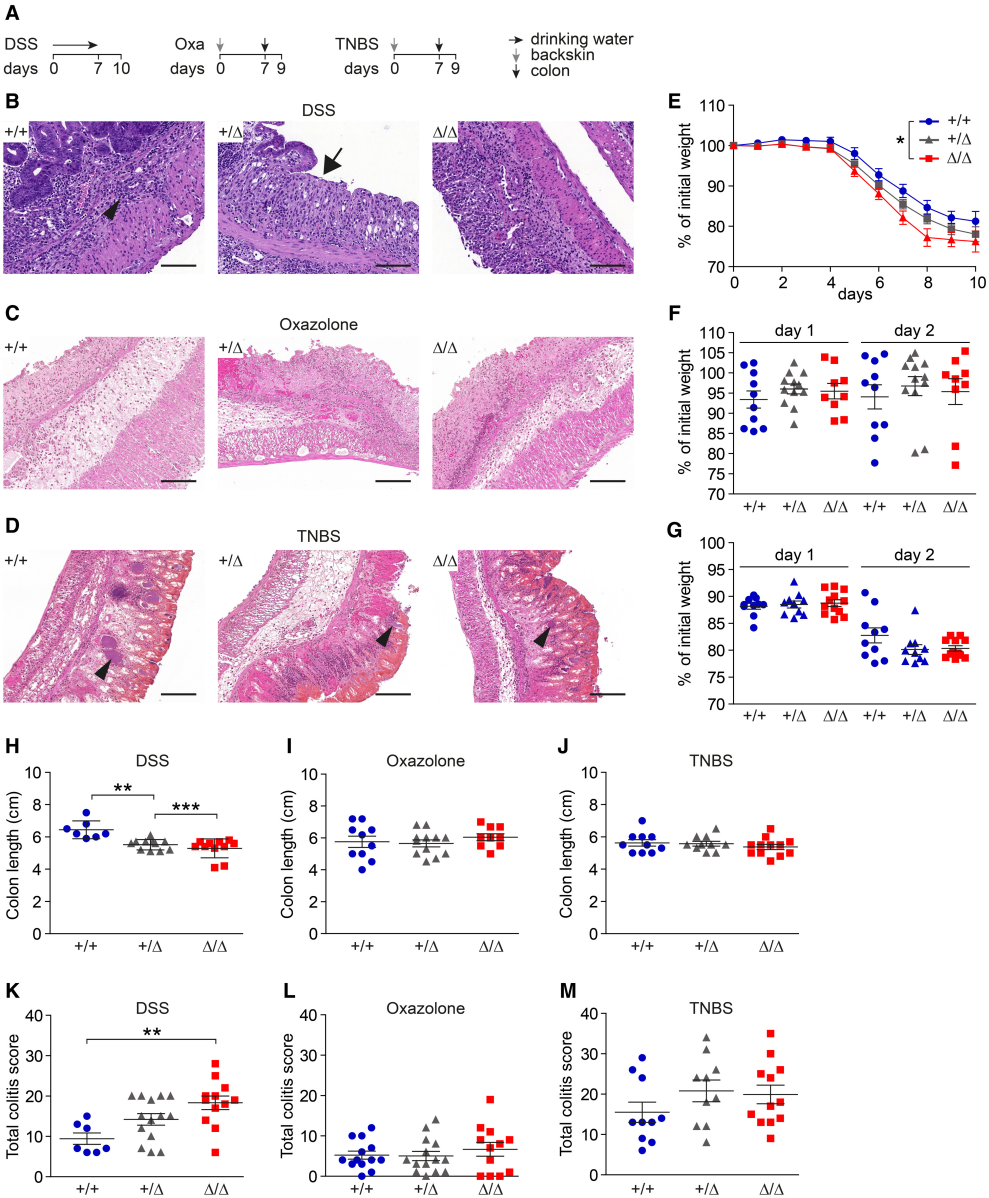
CDHR5^∆/∆^ mice are sensitive to DSS‐induced colitis ASchemes of DSS‐, oxazolone‐ and TNBS protocols for induction of acute colitis in CDHR5^+/+^, CDHR5^+/∆^ and CDHR5^∆/∆^ mice.BH&E‐stained images of DSS‐induced colitis showing inflammatory cells in the submucosa of CDHR5^+/+^ mice (arrowhead), loss of the epithelium in CDHR5^+/∆^ (arrow) and complete epithelial erosion in CDHR5^∆/∆^ mice. Scale bar = 100 μm.CH&E‐stained images of oxazolone‐induced colitis showing a gut area with significant inflammation and epithelial erosion. Scale bar = 100 μm.DH&E‐stained images of TNBS‐induced colitis showing severe epithelial necrosis with invading bacteria (arrowhead). Scale bar = 100 μm.EWeight loss of CDHR5^+/+^, CDHR5^+/∆^, and CDHR5^∆/∆^ mice during DSS‐induced colitis.FWeight loss of CDHR5^+/+^, CDHR5^+/∆^, and CDHR5^∆/∆^ mice after intracolonic application of oxazolone.GWeight loss of CDHR5^+/+^, CDHR5^+/∆^, and CDHR5^∆/∆^ mice after intracolonic application of TNBS.H–JQuantitation of colon shortening (indicator for colitis severity) in CDHR5^+/+^, CDHR5^+/∆^ and CDHR5^∆/∆^ mice with DSS (H), oxazolone (I) or TNBS (J) colitis.K–MTotal colitis score of CDHR5^+/+^, CDHR5^+/∆^, and CDHR5^∆/∆^ mice with DSS (K), oxazolone (L) or TNBS (M) colitis. Data information: Weight curves and scatter plots in (E–M) represent mean ± SEM. For (E) 11 CDHR5^+/+^, 14 CDHR5^+/∆^, and 14 CDHR5^∆/∆^ mice were analyzed. The AUC was calculated for each mouse and statistical analysis was performed with one‐way ANOVA and Tukey's multiple comparison test. For (F and G), each data point represents a mouse. Statistical analyses were performed separately for days 1 and 2 using one‐way ANOVA and Tukey's multiple comparison test. Differences are not significant. For (H–M) each data point represents a mouse. Statistical analyses of colon shortening (H–J) and colitis scores (K–M) were performed using one‐way ANOVA and Tukey's, Bonferroni's and Dunn's multiple comparison tests. All *post‐hoc* tests gave the same results. *P < 0.05, **P < 0.01, ***P < 0.001. Source data are available online for this figure.

**Figure EV3:**
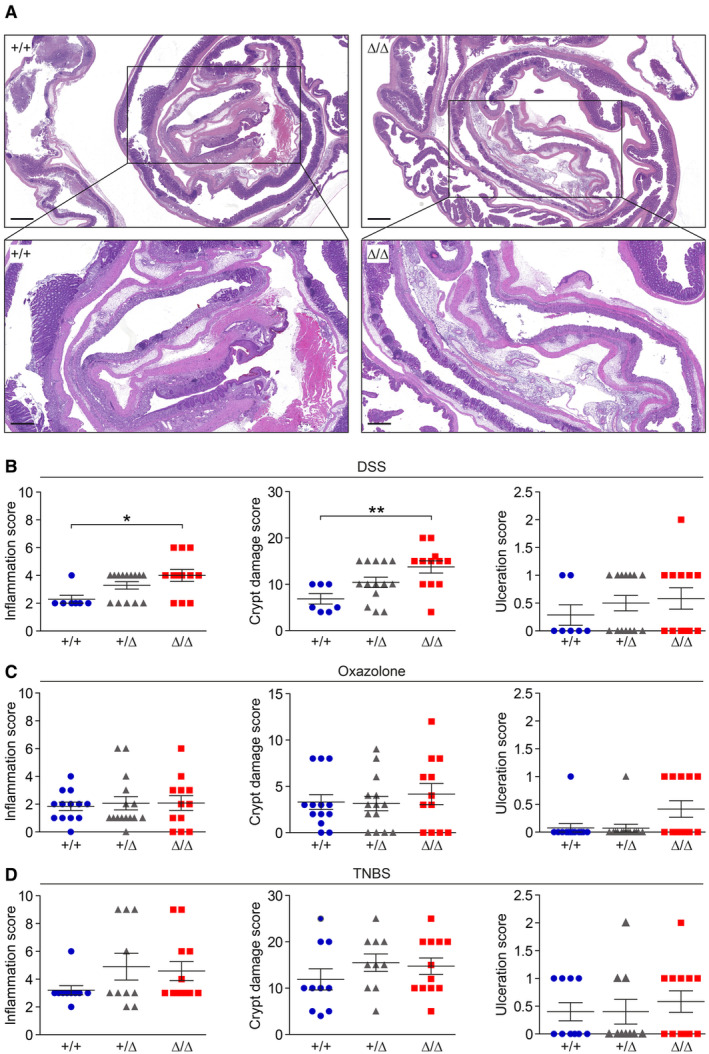
Inflammation and crypt damages scores are increased in CDHR5^∆/∆^ mice with DSS‐induced colitis ALow magnification H&E‐stained Swiss rolls of CDHR5^+/+^ and CDHR5^Δ/Δ^ mice suffering from acute DSS‐induced colitis. Scale bars = 1 mm of upper images and 500 μm of lower images. The positions of the lower images are indicated by the rectangles.B–DInflammation, crypt damage, and ulcerations scores of CDHR5^+/+^, CDHR5^+/∆^ and CDHR5^∆/∆^ mice with DSS (B), oxazolone (C) or TNBS (D) colitis. Scatter plots represent mean ± SEM. Each data point represents a mouse. All statistical analyses were performed using one‐way ANOVA and Tukey's, Bonferroni's and Dunn's multiple comparison tests. All *post‐hoc* tests gave the same results. *P < 0.05, **P < 0.01.

We used DSS‐, oxazolone, and TNBS acute colitis models (Fig 3A) to investigate the role of the IMAC in intestinal inflammation. Heterozygous CDHR5^+/∆^ mice were included in the *in vivo* studies to assess effects of reduced CDHR5 expression (Appendix Fig S1D and E). Spontaneous colitis was not observed in CDHR5^∆/∆^ mice (Fig EV2) but DSS and oxazolone induced significant inflammation and epithelial erosion in CDHR5^+/+^, CDHR5^+/∆^, and CDHR5^∆/∆^ mice (Figs 3B and C, and EV3A). Mice treated with TNBS showed severe epithelial necrosis with invading bacteria (Fig 3D). CDHR5^∆/∆^ and CDHR5^+/∆^ mice were more sensitive to DSS‐induced colitis as indicated by a pronounced weight loss (Fig 3E). In contrast, oxazolone did not induce a significant weight loss (Fig 3F) whereas TNBS induced a significant but similar weight loss in all genotypes (Fig 3G). Consistently, shortening of the colon length as indicator for colitis severity was increased in DSS‐treated CDHR5^∆/∆^ and CDHR5^+/∆^ mice but not oxazolone‐ or TNBS‐treated mice (Fig 3H–J). These data demonstrate that CDHR5 protects from DSS‐induced colitis but not from oxazolone or TNBS‐induced colitis. Histopathological evaluation of inflammation, crypt damage, and ulceration using H&E‐stained Swiss rolls confirmed these results and showed a significantly higher total colitis score in DSS‐treated but not oxazolone‐ or TNBS‐treated CDHR5^∆/∆^ mice (Fig 3K–M). The increased total DSS‐induced colitis score was mainly due to increased severity of inflammation and crypt damage whereas ulceration was only slightly affected (Fig EV3B). As expected, these histopathologic parameters were unchanged in CDHR5^∆/∆^ mice treated with oxazolone or TNBS (Fig EV3C and D). Our data demonstrate that CDHR5 protects against DSS‐induced colitis, which is based on induction of epithelial damage, but not against immune‐based oxazolone‐ or TNBS‐induced colitis.

### Intact intestinal barrier function in CDHR5 ^∆/∆^ mice

Possible causes for increased colitis in DSS‐treated CDHR5^∆/∆^ mice were investigated. The epithelial barrier prevents bacterial transmission and protects from colitis. Luminal bacteria penetrate the epithelium mainly through endocytosis (transcellular pathway; Yu, 2015; Hollander & Kaunitz, 2020) but rare reports have also shown penetration between epithelial cells (paracellular pathway; Necchi *et al*, 2007; Hollander & Kaunitz, 2020). Therefore, we investigated if IMAC ablation in CDHR5^∆/∆^ mice affects the epithelial barrier function through paracellular mechanisms. Immunofluorescence and 3D reconstruction of ZO‐1, claudin‐2, and claudin‐5 proteins revealed no obvious tight junction defects (Appendix Fig S6A and B) and glucose was resorbed with similar kinetics in CDHR5^+/+^ and CDHR5^∆/∆^ mice after oral gavage (Appendix Fig S6C). However, uptake of glucose occurs mainly through active transport, while a contribution of the paracellular pathway is unclear (Gromova *et al*, 2021). Therefore, we performed oral gavage experiments with a mixture of creatinine, 4 kDa FITC‐dextran and 70 kDa Rhodamine‐dextran to discriminate between paracellular pore (allows only creatinine passage), leak (allows creatinine and 4 kDa FITC‐dextran passage) and unrestricted (allows creatinine, 4 kDa FITC‐dextran and 70 kDa Rhodamine‐dextran passage) pathways (Chanez‐Paredes *et al*, 2021). We could not detect increased serum concentrations of creatinine after oral gavage in mice irrespective of the genotype (Appendix Fig S6D). 4 kDa FITC‐dextran and 70 kDa Rhodamine‐dextran were readily detected in gavaged mice but serum levels were similar in CDHR5^+/+^ and CDHR5^∆/∆^ mice (Appendix Fig S6E and F). These data demonstrate that loss of CDHR5 does not result in tight junction defects and paracellular leakage.

### Unchanged fecal microbiome in CDHR5 ^∆/∆^ mice

**Figure EV4:**
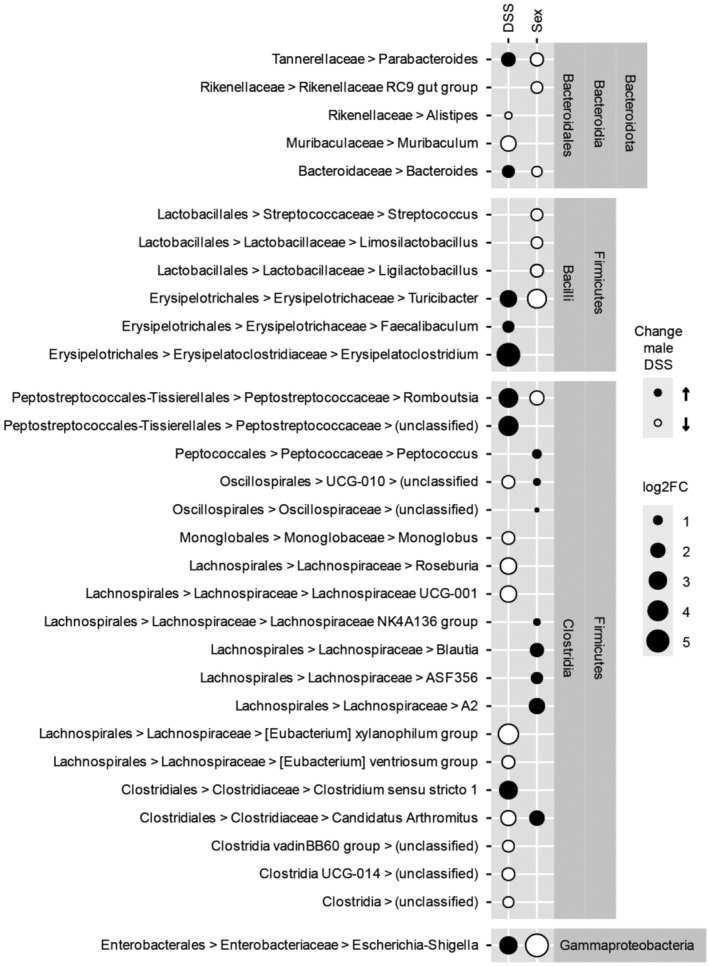
Differences in the microbiome composition of female versus male and untreated versus DSS‐treated mice A DESeq2‐derived bubble plot with sex‐specific and DSS‐specific log‐fold changes in microbiome composition on genus level is shown. The changes comprised several genera of the classes clostridia, bacilli and gammaproteobacteria as well as the order bacteroidales. “Unclassified” includes all taxa not classified at genus rank for given family or class.

Another cause for intestinal inflammation is dysbiosis of the gut microbiome. We therefore investigated whether the brush border defects in CDHR5^∆/∆^ mice affect the microbial growth environment and alter the composition of the gut microbiome. We distinguished between genotypes and sex and included fecal pellets from DSS‐treated mice in 16S rRNA gene amplicon‐based fecal microbiome analysis. Amplicon sequence variant (ASV) data were classified and further compared at the phylum, class, order, family, and genus levels (Appendix Fig S7). Principal component analyses of the ASV data revealed an overall sex‐specific difference in the microbiome composition of male and female mice (Appendix Fig S8A and B, and Fig EV4), which was maintained in stratified CDHR5^+/+^ and CDHR5^∆/∆^ mice (Appendix Fig S8C and D). However, there was no difference in the microbiomes of CDHR5^+/+^ and CDHR5^∆/∆^ mice before (Appendix Fig S8E) and after sex stratification (Appendix Fig S8F and G). DSS treatment significantly altered the fecal microbiome (Appendix Fig S8H and I, and Fig EV4) in both, female and male mice without genotype stratification (Appendix Fig S8J and K) and in CDHR5^+/+^ and CDHR5^∆/∆^ mice without sex stratification (Appendix Fig S8L and M). Interestingly, DSS treatment reduced sex differences (Appendix Fig S8N) while no genotype‐specific effect on the microbiome composition was identified (Appendix Fig S8O). Thus, our data revealed differences in the microbiome composition of male and female as well as untreated and DSS‐treated mice. However, no genotype‐specific effects were found, showing that microvillus disorganization in CDHR5^∆/∆^ mice does not affect the overall composition of the fecal microbiome.

### Increased susceptibility of CDHR5 ^∆/∆^ mice to DSS‐induced apoptosis

**Figure 4:**
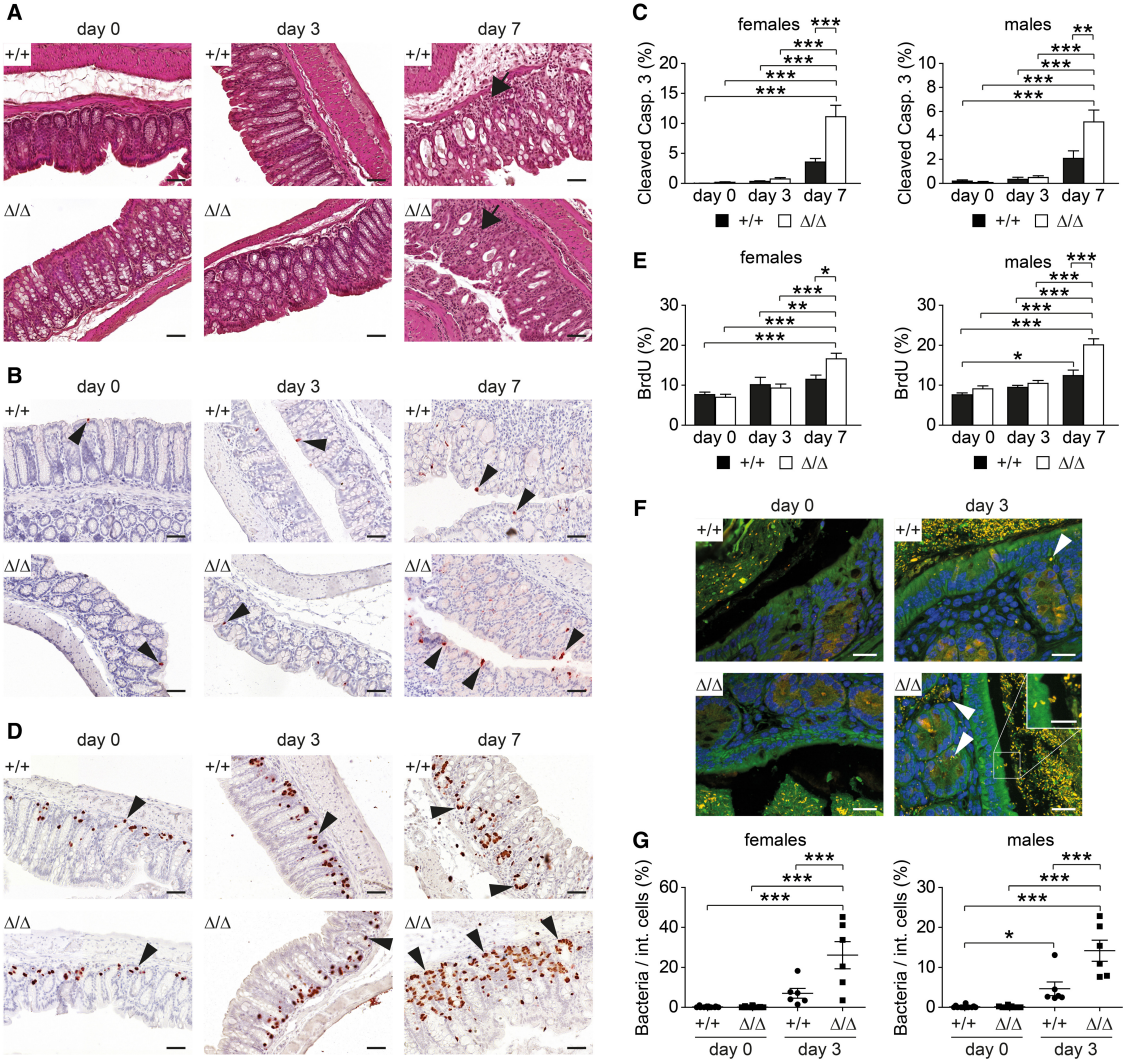
CDHR5 protects from bacterial invasion and epithelial apoptosis during DSS‐induced colitis AH&E‐stained colon sections of day 0, day 3, and day 7 DSS‐treated CDHR5^+/+^ and CDHR5^∆/∆^ mice. Infiltrating inflammatory cells are indicated by arrows (day 7). Images were taken from Swiss rolls. Scale bar = 50 μm.BColon sections of day 0, day 3, and day 7 DSS‐treated mice, stained for the apoptotic marker cleaved caspase‐3. Positive cells are indicated by arrowheads. Images were taken from Swiss rolls. Scale bar = 50 μm.CQuantitation of apoptosis in female and male mice (3 mice per sex and genotype, 10 consecutive regions per Swiss roll were used for quantitation of cleaved caspase‐3‐positive cells per total cells).DColon sections of day 0, day 3, and day 7 DSS‐treated mice, stained for the proliferation marker BrdU. Positive cells are indicated by arrowheads. Images were taken from Swiss rolls. Scale bar = 50 μm.EQuantitation of proliferation in female and male mice (3 mice per sex and genotype, 5 consecutive regions per Swiss roll were used for quantitation of cleaved caspase‐3‐positive cells per total cells).FSpinning disc fluorescence images of colon tissue from day 0 and day 3 DSS‐treated mice stained with the Cy3‐labeled FISH probe EUB338 for bacteria (orange) and DAPI for nuclei (blue). The channel for green autofluorescence was included to delineate cell boundaries. Bacterial FISH signals in the mucosa are indicated by arrowheads. The inset shows a high magnification of bacterial invasion into the epithelium of CDHR5^∆/∆^ mice. Images were taken from stained Swiss rolls. Scale bar = 20 μm. Scale bar in inset = 10 μm.GQuantitation of bacterial FISH signals in the mucosa of female and male mice. Bacteria invading the mucosa were counted and related to the total number of intestinal cells (epithelial and lamina propria cells), quantified by counting blue nuclei with ImageJ (≥ 3 mice per sex and genotype, the two most affected gut regions per Swiss roll were used for quantitation of day 3 DSS‐treated mice). Data information: Bar diagrams and scatter plots in (C, E and G) represent mean ± SEM. All statistical analyses were performed using one‐way ANOVA and Tukey's multiple comparison test. *P < 0.05, **P < 0.01, ***P < 0.001. Source data are available online for this figure.

The DSS‐induced colitis model is mainly based on the induction of epithelial damage. Therefore, we characterized early events of DSS treatment in female and male CDHR5^+/+^ and CDHR5^∆/∆^ mice. On day 3 of DSS treatment, no major histopathology was observed except for a slight infiltration of immune cells into the mucosa. By day 7, the inflammation was much more severe and epithelial erosion became evident (Fig 4A). Staining for apoptotic cells showed few apoptotic cells on day 0 and day 3 of DSS treatment, but the number increased significantly on day 7 (Fig 4B). Importantly, male and female CDHR5^∆/∆^ mice showed significantly more apoptotic cells than corresponding CDHR5^+/+^ control mice (Fig 4C). Epithelial damage in the gut is associated with a regenerative response that leads to proliferation of crypt stem cells. Staining for BrdU incorporation showed a slight increase of regenerative proliferation on day 3 of DSS treatment compared to day 0 in female and male CDHR5^+/+^ and CDHR5^Δ/Δ^ mice (Fig 4D and E). By day 7, regenerative proliferation was evident and induced to a greater extent in CDHR5^∆/∆^ mice than in CDHR5^+/+^ mice (Fig 4D and E). These data demonstrate that DSS‐induced epithelial apoptosis is exacerbated in CDHR5^∆/∆^ mice, but regenerative proliferation of crypt stem cells is functional.

### Enhanced bacterial invasion in DSS‐treated CDHR5 ^∆/∆^ mice

**Figure EV5:**
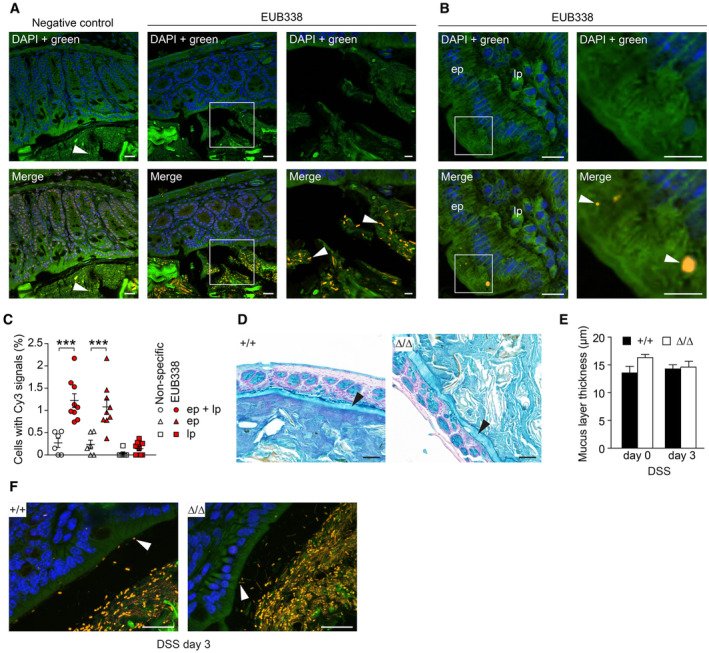
Evaluation of bacterial FISH analysis and mucus layer permeability after DSS treatment in CDHR5^+/+^ and CDHR5^∆/∆^ mice Spinning disc fluorescence images of mouse colon for Cy3‐labeled bacteria stained with the pan‐bacterial FISH probe EUB338 (orange), nuclear DAPI (blue) and green autofluorescence (upper images without Cy3 and lower images merged). For the negative control, a Cy3‐labeled non‐specific probe was used. Non‐specific signals were observed in cell nuclei and in feces (left images). Non‐specific fecal signals were double‐positive and showed also green autofluorescence (arrowhead). Accordingly, nuclear signals and double‐positive signals were interpreted as false‐positives and excluded from the analysis. Many fecal bacteria could be detected with the FISH probe (middle images with low magnification and right images with high magnification, the rectangle in the middle images marks the position of the right images). The bacterial signals (arrowheads) showed no green autofluorescence. Scale bar = 20 μm (low magnification) or 5 μm (high magnification).Example for epithelial Cy3‐positive signals (arrowheads) that represent bacteria (low magnification left images and high magnification right images, the rectangle in the middle images marks the position of the right images). Upper images are without Cy3 and lower images are merged. Scale bar = 10 μm (low magnification) or 5 μm (high magnification).Scatter plots with quantitation of Cy3‐positive signals (without green autofluorescence) in the mucosa on slides with negative control probe (non‐specific background) and with FISH probe EUB338. About five times more signals were detected with EUB338 in the mucosa (epithelium plus lamina propria) and in the epithelium only than with the non‐specific probe. All counts were performed on multiple images obtained with intestinal sections from ≥ 3 mice. Scatter plots represent mean ± SEM. Statistical analysis was performed using one‐way ANOVA and Tukey's multiple comparison test. ***P < 0.001. ep, epithelium; lp, lamina propria.Alcian blue staining of the colonic mucus layer (arrowhead) of untreated CDHR5^+/+^ and CDHR5^∆/∆^ mice. Scale bar = 100 μm.Quantitation of the colonic mucus layer thickness in untreated (day 0) and DSS‐treated (day 3) mice. Bars represent data ± SEM (3 CDHR5^+/+^ and ≥ 3 CDHR5^∆/∆^ mice, ≥ 11 thickness measurements and calculation of mean per mouse). Statistical analyses were performed using unpaired Student's *t*‐test for day 0 and day 3 separately. Differences are not significant.Spinning disc fluorescence images showing invasion of bacteria into the colonic mucus layer (arrowheads) of CDHR5^+/+^ and CDHR5^∆/∆^ mice treated with DSS for 3 days. Colon sections were stained by FISH for bacteria with Cy3‐labeled EUB338 (orange). Nuclei were stained with DAPI (blue) and the channel for green autofluorescence was included to delineate cell boundaries. Scale bar = 20 μm.

DSS increases the permeability of the mucus layer to bacteria as early as 12 h after the start of treatment (Johansson *et al*, 2014). This would put bacteria in direct contact with the disorganized brush border of CDHR5^∆/∆^ mice. Therefore, bacterial invasion was monitored in DSS‐treated mice at day 0 and day 3 by spinning disc fluorescence microscopy of FISH‐stained Swiss rolls using the common Cy3‐labeled pan‐bacterial probe EUB338 (Canny *et al*, 2006; Wu *et al*, 2014). Control staining was performed to assess background signals. A Cy3‐labeled negative control probe showed false‐positive signals in cell nuclei as well as fecal double‐positive signals with green autofluorescence and simultaneous emission in the Cy3 channel (Fig EV5A). However, fecal EUB338‐derived signals were clearly of bacterial origin and lacked green autofluorescence (Fig EV5A). We therefore excluded nuclear signals and green/Cy3 double‐positive signals from the analysis (Fig EV5B). A comparison of results with Cy3‐labeled negative control and EUB338 probes revealed the impact of false‐positives not yet excluded. The results showed that although additional false‐positive signals were present, these could be neglected (Fig EV5C). Counting of bacterial signals in mucosal cells (epithelium and lamina propria) of untreated day 0 mice showed little bacterial invasion (Fig 4F and G). The mucus layer lacked bacterial signals (Fig 4F) and the thickness of the mucus layer was not reduced in CDHR5^∆/∆^ mice (Fig EV5D and E). These results indicate that bacteria rarely contact and invade the disorganized brush border of untreated CDHR5^+/+^ and CDHR5^∆/∆^ mice. Next, we examined bacterial invasion in mice on day 3 of DSS treatment. The thickness of the mucus layer was not affected by DSS treatment (Fig EV5E). However, bacterial signals contacting the epithelial surface were readily detected in the colon of CDHR5^+/+^ and CDHR5^Δ/Δ^ mice indicating that the mucus layer became permeable (Fig EV5F). Importantly, DSS‐treated male and female CDHR5^∆/∆^ mice showed significantly more bacterial invasion than corresponding CDHR5^+/+^ control mice (Fig 4F and G). These data demonstrate that densely packed microvilli prevent bacterial invasion in the case of increased mucus layer permeability.

### Reduced expression of CDHR5 and TMIGD1 in enterocytes of diseased gut areas in patients with UC

**Figure 5:**
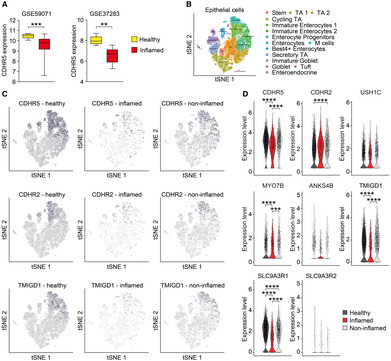
CDHR5 and TMIGD1 are downregulated in enterocytes of diseased gut areas of patients with UC ADownregulation of CDHR5 in mucosal samples of patients with UC in the GSE59071 (11 healthy and 97 diseased individuals) and GSE37283 (5 healthy and 11 diseased individuals) microarray datasets when compared to healthy tissue. **P < 0.01, ***P < 0.001.BtSNE plot of the total epithelial cell population of healthy individuals and UC patients.CtSNE plots of stratified epithelial cells from healthy individuals as well as epithelial cells from inflamed or non‐inflamed gut areas of UC patients indicating the expression of CDHR5, CDHR2, and TMIGD1 in enterocytes.DViolin plots for expression of IMAC components in enterocytes from healthy individuals as well as enterocytes from inflamed or non‐inflamed gut areas of UC patients. Statistics was performed with the Wilcoxon Rank Sum test. *P*‐value adjustment for multiple testing was corrected using Bonferroni correction. ***P < 0.001 or ****P < 0.0001. Data information: For (B–D) the scRNA‐seq dataset of Smillie was used to assess expression of IMAC components in the healthy colonic mucosa as well as inflamed and non‐inflamed gut areas of patients with UC (Smillie *et al*, 2019). Source data are available online for this figure.

We used published microarray and scRNA‐seq datasets from human UC patients to examine the expression of IMAC components in diseased and healthy gut areas. Microarray data sets (GSE37283, GSE59071) showed downregulation of CDHR5 in the mucosa of UC patients when compared to healthy donors (Fig 5A), which is consistent with recent data (Han *et al*, 2021). scRNA‐seq of healthy donors and patients with UC containing the intestinal epithelial cell populations have been previously described (Smillie *et al*, 2019). Analysis of total epithelial cells (healthy, inflamed, and non‐inflamed) in this dataset revealed CDHR5 expression mainly in enterocytes and to some extent in goblet cells, but not in stem cells or transit amplifying cells (Appendix Fig S9A–C). A similar expression pattern was found for CDHR2 (Appendix Fig S9D and E), MYO7B (Appendix Fig S9F), TMIGD1 (Appendix Fig S9G) and SLC9A3R1 (Appendix Fig S9H). The other IMAC components showed low expression that could not clearly be attributed to enterocytes (Appendix Fig S9I–K). Almost no CDHR5‐expressing cells were present in stromal or immune cell populations from UC patients (Appendix Fig S9L–O). We stratified healthy and diseased epithelial cell populations of the scRNA‐seq dataset to assess whether inflammation affects the pattern of protocadherin and TMIGD1 expression. Similar epithelial cell populations expressed CDHR5, CDHR2, and TMIGD1 in the healthy mucosa and in the inflamed and non‐inflamed mucosa of patients with UC (Fig 5B and C). However, the expression of CDHR5 and TMIGD1 was significantly downregulated in enterocytes of inflamed areas in patients with UC, while expression of CDHR2 was not markedly decreased (Fig 5D). These data suggest that downregulation of CDHR5 and TMIGD1 contributes to the development of colitis in patients with UC.

## Discussion

To date, no pathological consequences of microvillus crosslinking defects have been reported. However, patients with homozygous deletion of USH1C, which links intracellular protocadherin domains via MYO7B with the central microvillus actin bundle, developed inflammatory enteropathy (Bitner‐Glindzicz *et al*, 2000). In addition, mice deficient for the actin‐bundling protein Plastin 1, which anchors the rootlets of central actin bundles in microvilli to the terminal web, displayed brush border defects and were more sensitive to DSS‐induced colitis (Grimm‐Gunter *et al*, 2009). Our study shows that CDHR5‐dependent microvillus crosslinking in the intestinal brush border prevents DSS‐induced bacterial invasion and colitis. The *in silico* analyses of scRNA‐seq data from patients with UC suggest that CDHR5 has a similar function in humans and protects from development of IBD.

Intermicrovillar adhesion is mediated by heterophilic interaction of CDHR2 and CDHR5 but species‐specific homophilic protocadherin interactions have also recently been identified by structural and bead aggregation assays. The studies showed homophilic CDHR5 but no homophilic CDHR2 interactions in mice, which was reversed in humans (Gray *et al*, 2021). This indicates that a residual microvillus crosslinking function is still present in CDHR2‐deficient mice due to homophilic CDHR5 interactions. In contrast, CDHR5‐deficient mice should lack any protocadherin‐mediated microvillus crosslinking. However, the brush border phenotypes of CDHR2‐deficient mice (Pinette *et al*, 2019) and CDHR5‐deficient mice were quite similar, suggesting that a possibly weak homophilic interaction between CDHR5 is not sufficient to significantly improve brush border organization in CDHR2‐deficient mice. Recently, the transmembrane protein TMIGD1 was identified as key component of a second IMAC at the base of microvilli. TMIGD1‐deficient mice also showed brush border defects (Hartmann *et al*, 2022). These data suggest that CDHR2, CDHR5, and TMIGD1 have non‐redundant functions and that both IMAC complexes are required for proper brush border organization.

Cell culture experiments have shown that CDHR5 prevents nuclear translocation of ß‐catenin through direct protein–protein interaction (Hinkel *et al*, 2012). However, CDHR5‐deficient mice developed normally and had no apparent pathology, suggesting that under physiological conditions CDHR5 has no major *in vivo* function apart from microvillus crosslinking. This is supported by the fairly restricted expression of CDHR5 in intestinal and renal epithelial cells and the exclusive localization of the protein to the brush border (Moulton *et al*, 2004). Furthermore, our RNA sequencing data from epithelial cells of CDHR5‐deficient mice revealed only few deregulated genes, all of which were not ß‐catenin targets. However, it is conceivable that CDHR5‐mediated ß‐catenin regulation is important in pathological conditions such as colon cancer development or mucosal regeneration after injury.

The exact trigger of IBD is unknown. Western dietary habits play a big role, but it has become clear that they can aggravate, but are not the trigger of IBD (Ban *et al*, 2022). Maintaining the intestinal epithelial barrier may be a key requirement for IBD prevention (Martini *et al*, 2017). We have not observed spontaneous colitis in CDHR5‐deficient mice nor has it been reported in CDHR2‐ and TMIGD1‐deficient mice (Pinette *et al*, 2019; Hartmann *et al*, 2022). Our data suggest that this is most likely due to the protective effect of the intestinal mucus layer but this has yet to be proven. The mucus layer is the first line of defense against bacterial invasion and colitis. Several genetic mouse models with profound mucus layer permeability, such as MUC2, NHE3, and C1GALT1 knock‐out mice, developed colitis (Johansson *et al*, 2014). In humans, such severe mucus layer defects might be incompatible with life. However, there are lifestyle conditions that lead to a moderate increase in mucus layer permeability, such as low‐fiber Western diet (Alemao *et al*, 2021). Short‐term treatment with DSS may recapitulate this condition in experimental mice since the mucus layer becomes permeable to bacteria 12 h after DSS exposure (Johansson *et al*, 2014). A thinner mucus layer was also found in patients with active UC compared to patients in remission, and epithelial attachment and penetration of bacteria was frequently documented in IBD (Kleessen *et al*, 2002; Swidsinski *et al*, 2002; Johansson *et al*, 2014). These data indicate that increased permeability of the colonic mucus layer with subsequent exposure of the epithelial surface to bacteria is generally associated with development of colitis in mice and humans (Johansson *et al*, 2014). A well‐organized brush border may still prevent bacterial penetration but a combination of increased mucus layer permeability and microvillus fanning might allow bacteria to gain access to cholesterol‐rich lipid rafts and caveolae at the base of the intermicrovillus cleft. Under this condition, even non‐invasive mucosal bacteria, which do not have a specialized molecular machinery for cell invasion, could potentially hijack lipid raft/caveolae‐mediated endocytosis for cell penetration (Wu *et al*, 2014). In addition, apoptosis of epithelial cells was observed in patients with IBD, which may decrease intestinal barrier function (Blander, 2016). Epithelial apoptosis was also increased in DSS‐treated CDHR5‐deficient mice, but our data suggest that it may be a secondary event, due to prior bacterial invasion.

Several studies demonstrated important functions of T cells in IBD. We examined the protective effect of CDHR5 in oxazolone and TNBS models (Neurath, 2014; Zundler *et al*, 2019). Oxazolone and TNBS induce a hapten‐specific T cell‐mediated immune response and colitis with histopathological features of human UC and CD, respectively (Wirtz *et al*, 2007, 2017; Weigmann & Neurath, 2016). CDHR5‐deficient mice were not more sensitive to these colitis models, which result in rapid (within 1–2 days) immunological destruction of intestinal epithelial cells (Wirtz *et al*, 2007, 2017). Protective effects of the mucus layer or brush border may not be effective in these colitis models because epithelial cells are immunologically destroyed and bacteria can enter via the unrestricted route (i.e., no barrier because the epithelium is lost). Rather, the lack of difference in these models reinforces the importance of brush border crosslinking in the DSS model, which is based on luminal effects on epithelial cells. However, it is conceivable that brush border defects and dysregulated adaptive T cell responses synergistically aggravate colitis in patients with IBD. Furthermore, inflammatory events could act as a positive feed‐forward loop in IBD in two ways. First, IL‐10‐, and TLR5 knock‐out mice developed immune‐based colitis associated with increased mucus layer permeability, suggesting a link between mucus properties and the immune system (Johansson *et al*, 2014). Second, pro‐inflammatory IFN‐γ induced myosin light chain kinase (MLCK) and the corresponding phosphorylation of MLC in the terminal web of intestinal epithelial cells. This resulted in terminal web contraction and apical membrane protrusion. A corresponding arc model has been proposed, in which terminal web contraction and apical protrusion exert a fanning force on the microvilli (Wu *et al*, 2014; Yu, 2015). The IMAC may withstand the fanning force and maintain brush border organization. However, without the IMAC, the cytokine‐induced arc formation would actually enlarge gaps for bacterial entry (Mödl *et al*, 2022). Interestingly, increased phosphorylation of MLC was also observed in the inflamed mucosa of IBD patients, suggesting that arcing also occurs in humans (Blair *et al*, 2006).

**Figure 6:**
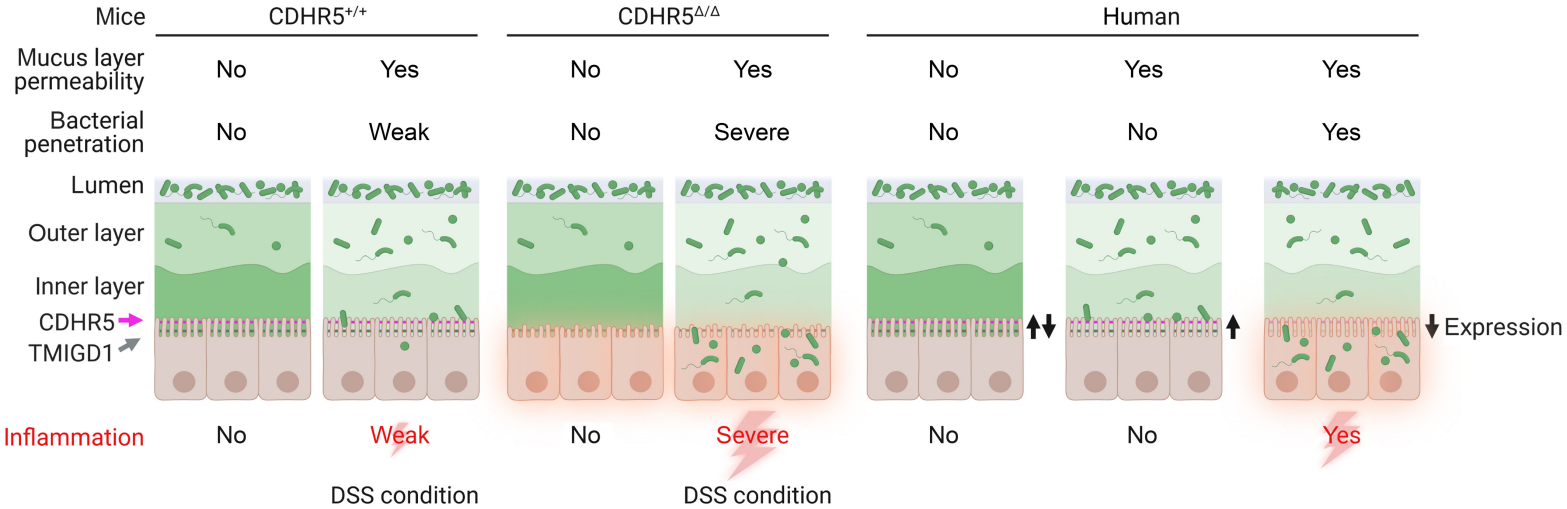
Model how CDHR5 protect against colitis and IBD development Under physiological conditions, the mucus layer is impermeable to bacteria and they cannot reach the brush border. Consequently, microvillus crosslinking defects in CDHR5^∆/∆^ mice do not lead to spontaneous colitis. Treatment with DSS increases the permeability of the mucus layer. Our data suggest that the densely packed brush border in CDHR5^+/+^ mice acts as a barrier against bacteria that reach the surface of epithelial cells, thereby limiting colitogenic effects of DSS. The disorganized brush border in CDHR5^∆/∆^ mice might lack this barrier capacity. Bacteria can enter and cause severe inflammation. In humans, low expression of CDHR5 or TMIGD1 (e.g., due to familial low expression or epigenetic silencing) could also impair microvillus crosslinking ability. We speculate that most of these people remain unaffected, but they may be at risk of developing inflammatory bowel disease, for example when they consume Western diets that lead to increased mucus layer permeability. Image created with BioRender.

Taken together, our results demonstrate that microvillus crosslinking in the brush border of gut epithelial cells protects against colitis. The protective effect would be particularly important for people on a low‐fiber Western diet, as these eating habits increase the permeability of the colonic mucus layer and allow bacteria to reach the brush border (Alemao *et al*, 2021). Accordingly, local epithelial or general familial low expression of IMAC components combined with consumption of a Western diet could be an important risk factor for IBD. The disease may begin from patches of epithelial cells with reduced expression of CDHR5 and TMIGD1, which represent entry points for bacterial invasion and immune cell activation (Fig 6). In this case, drugs that increase the expression of IMAC components could be beneficial for patients with IBD as they would increase the number of microvillus crosslinks and make the brush border more rigid and impermeable. Mesalazine treatment is commonly used for IBD therapy, and the beneficial effects have been attributed primarily to its anti‐inflammatory effects (Ye & van Langenberg, 2015). However, mesalazine also induces CDHR5 expression in cell cultures (Parenti *et al*, 2010, 2018; Bersuder *et al*, 2022). Thus, mesalazine is a potential drug candidate to strengthen the brush border, but it remains to be shown whether it can induce CDHR5 expression *in vivo*.

## Materials and Methods

### Study design

The aim of this study was to investigate the impact of microvillus crosslinking defects on gut barrier function and the development of colitis. A knockout mouse model with microvillus crosslinking defects containing a deletion of the IMAC component CDHR5 was generated and used for mechanistic and colitis studies.

### Generation of CDHR5 ^∆/∆^ mice

A targeting vector for floxed fra1 alleles was used as backbone for the CDHR5 targeting vector (Eferl *et al*, 2004). The long homology arm of fra1 was replaced by PCR cloning for an 8.8 kb long homology arm of CDHR5 comprising the complete gene and the polyadenylation signal. The upper loxP site (followed by an artificial EcoRI site for Southern blot analysis) was cloned into the SphI site of intron 3. The short homology arm of fra1 was replaced by PCR cloning for a 1.7 kb short homology arm of CDHR5 comprising a genomic fragment immediately downstream of the polyadenylation signal. Long and short homology arms were separated by a pGK‐Neo selection cassette, flanked by FRT sites, followed by the lower loxP site, a 0.2 kb splice acceptor sequence and an EGFP reporter gene. The gene for diphtheria toxin was used for negative selection of random insertions. The CDHR5 targeting vector was electroporated into HM1 embryonic stem cells for homologous recombination. Positive clones were identified by nested PCR and Southern blot analysis and used for blastocyst injection. After germline transmission, the pGK‐Neo selection cassette was removed *in vivo* with ß‐Actin‐Flpe deleter mice. Resulting CDHR5^flox/+^ mice were crossed with ß‐Actin‐Cre deleter mice to generate heterozygous CDHR5^+/∆^ mice, which were intercrossed to generate CDHR5^∆/∆^ mice.

### Housing, experimentation, and genotyping of mice

All the mouse experiments were performed in accordance with Austrian and European laws and with the general regulations specified by the Good Scientific Practice guidelines of the Medical University of Vienna (BMWFW‐66.009/0189‐WF/V/3b/2015 & 2020‐0.448.823). Mice were housed in the animal facility of the Medical University of Vienna (https://biomed‐forschung.meduniwien.ac.at) at constant room temperature, with 12 h light/12 h dark cycles and under specific pathogen free (SPF) conditions. They received a standard diet (LASQC diet, Rod16, Auto from Altromin) and drinking water at libitum and were housed in ventilated GM500 cages (Tecniplast) with standard wood chips. Sex‐ and age‐matched littermates (6–9 weeks old) of CDHR5^+/+^, CDHR5^+/∆^, and CDHR5^∆/∆^ mice in the C57BL/6 genetic background were used for experiments. Genotyping was performed with toe DNA using primers P1 5′‐CCAGACAGCCTCACACAGAA‐3′, P2 5′‐GTTGCTCATGGTGAAGCAGA‐3′ and P3 5′‐GACACGCTGAACTTGTGGCCGTTTA‐3′ resulting in 261 bp amplicons of CDHR5 wildtype alleles and 528 bp amplicons of deleted alleles.

### Colitis induction

DSS‐mediated colitis was induced with 2.0% or 1.5% (female or male mice) DSS (w/v) in the drinking water for 7 days, followed by a 3‐day recovery period (Crncec *et al*, 2018). Oxazolone‐ and TNBS‐mediated colitis were induced as described previously (Wirtz *et al*, 2007, 2017; Weigmann & Neurath, 2016). Briefly, mice were anesthetized and a 1.5 × 1.5 cm area was shaved on the back skin between the shoulders. Presensitization solution (150 μl 3% oxazolone or 1% TNBS) was applied on the shaved area. Seven days after presensitization, 100 μl of 1% oxazolone or 2.5% TNBS solution was injected 4 cm deep into the colon with a silicone catheter for induction of colitis. Mice were sacrificed 2 days after colon injection and Swiss rolls were prepared.

### Colitis scoring

Male mice were used for colitis scoring. Colitogenic mice were routinely monitored for weight loss, diarrhea, and fecal blood loss. They were sacrificed in case of severe disease and weight loss greater than 30%. BrdU was injected into experimental mice before killing. For colitis scoring, scanned H&E‐stained Swiss rolls of colons were examined under the microscope in a blinded fashion according to the following criteria: inflammation score 0 = rare or no inflammatory cells in lamina propria, 1 = increased numbers of granulocytes in lamina propria, 2 = confluence of inflammatory cells extending to submucosa, 3 = transmural extension of inflammatory infiltrate; crypt damage 0 = none, 1 = loss of basal 1/3 of the crypt, 2 = loss of basal 2/3 of the crypt, 3 = entire crypt loss, 4 = change of epithelial surface with erosion, 5 = confluent erosion; ulceration 0 = none, 1 = 1–2 ulcers focally, 2 = 3–4 ulcers focally, 3 = confluent ulceration. The individual scores were added up to give a maximum of 11. This score was multiplied by a multiplication factor on the basis of the area affected: 1 = 0–25% of the colon, 2 = 25–50% of the colon, 3 = 50–75% of the colon, 4 = 75–100% of the colon to give a final maximum score of 44.

### Immunohistochemistry

IHC was performed as described previously (Crncec *et al*, 2015). The QuPath freeware was used for detection of synaptophysin‐positive, lysozyme‐positive, BrdU‐positive, and cleaved Caspase 3‐positive cells. The following antibodies were used: anti‐synaptophysin (GeneTex GTX100865), anti‐lysozyme (Dako A0099), anti‐BrdU (abcam, #ab6326), and anti‐cleaved Caspase 3 (CST, #9661L).

### Immunofluorescence

Immunofluorescence of IMAC components, tight junction proteins and apical markers (DPPIV, IAP, NHE3, P‐Ezrin, and Ezrin) were performed as described previously (Gonzalez‐Mariscal *et al*, 2011; Pinette *et al*, 2019). Expression profiles from brush border LSM images were analyzed with Fiji ImageJ. The line tool was used with a constant length of 4 μm and a line width of 12 pixel. The end of the line pointed in the luminal direction and the center of the line was at the center of the maximal ezrin signal of the brush border. Several profiles were collected from each individual staining and further processed in R 4.2.2. Expression profile signals were normalized to 1, gene of interest profiles were aligned relative to the spatially normalized maxima of the ezrin profiles to center all measurements. Profile signals above 0.7 were subjected to the cor function of the stats package (3.6.2) of R to estimate the Pearson correlation coefficient for each genotype and gene of interest versus the appropriate ezrin profile. Correlation coefficients were compared by the Wilcoxon‐Mann–Whitney test, a *P*‐value below 0.05 was considered to be significant. The ZEN software (Zeiss) was used for 3D reconstruction of tight junctions and for measurement of fluorescent signal lengths after IF staining for apical markers. The relative amount of DPPIV, IAP, NHE3, P‐Ezrin, and Ezrin proteins was quantified with ImageJ using the profile intensity of the fluorescent signals and calculation of the areas under the curve. The following antibodies were used: anti‐IAP (Thermo Fisher Scientific #PA5‐22210), anti‐SI (Santa Cruz #sc‐393424), anti‐DPPIV (R&D Systems #AF954), anti‐NHE3 (Novus Biologicals #NBP1‐82574), anti‐p‐ERM (Cell Signaling Technology #3726), anti‐ezrin (Cell Signaling Technology #3145, Abcam ab4069), anti‐CDHR5 (Sigma HPA009173), anti‐USH1C (Sigma HPA027398), anti‐CDHR2 (Sigma HPA012569), anti‐MYO7B (Sigma HPA039131), anti‐claudin‐2 (Fisher Scientific #15377254 and Cell Signaling Technology #48120S), anti‐claudin‐5 (Fisher Scientific #10777223), and anti‐ZO‐1 (Fisher Scientific #10129012).

### Fluorescent *in situ* hybridization

FISH was performed as described previously with Cy3 end‐labeled probes for universal bacteria (EUB338 5′‐GCTGCCTCCCGTAGGAGT‐3′) and negative control (non‐EUB338 5′‐ACATCCTACGGGAGGC‐3′; Wu *et al*, 2014). Intestinal tissues were fixed in Carnoy's solution without flushing of the gut and embedded in paraffin. 4 μm sections were mounted on glass slides, deparaffinized, treated with 1% Triton X‐100 for 90 s and then incubated in PBS containing 5 mg/ml lysozyme for 20 min at 37°C. Slides were rinsed with water and air‐dried. Sections were preincubated at 46°C for 60 min in a hybridization buffer containing 0.9 M NaCl, 20 mM Tris–HCl, 15% formamide, and 0.01% SDS (pH 7.4). Prewarmed hybridization buffer containing 0.1 mM oligonucleotide probe was added to the tissue sections. The slides were incubated overnight in a dark humid chamber at 46°C, rinsed with double distilled water and stained with Hoechst dye for cell nuclei. Images were captured under a spinning disc fluorescence microscope (Olympus IXplore SpinSR).

### Villus morphometry

Villus morphometric analysis was performed with scanned H&E‐stained Swiss rolls using a Pannoramic Midi slidescanner and the Pannoramic Viewer annotation software (3DHistech) and villus parameters as described previously (Gulbinowicz *et al*, 2004).

### Measurement of intestinal barrier integrity

The method to differentiate between tight junction‐dependent or independent intestinal barrier loss has been previously described (Chanez‐Paredes *et al*, 2021). Briefly, mice were gavaged with 100 μl of a mixture of 80 mg/ml 4 kDa FITC‐dextran, 40 mg/ml 70 kDa rhodamine‐dextran, and 100 mg/ml creatinine. Mice were bled 3 h after gavage and plasma analyzed using a spectrophotometer at 495/525 nm and 555/585 nm for FITC and rhodamine intensity, respectively. To analyze the creatinine concentration, plasma was filtered using 10 kDa spin columns and the assay was performed using a creatine assay kit (Sigma) according to the manuals. The samples were analyzed using the spectrophotometer at 538/587 nm.

### Electron microscopy

Samples for electron microscopy were prepared with segments of colon and small intestine. For SEM, they were washed with cold PBS supplemented with 1.2 mM CaCl_2_ and 1 mM MgCl_2_ (pH 7.2), fixed overnight at 4°C with 3% glutaraldehyde in SEM buffer (0.1 M sucrose in 0.1 M Na–phosphate, pH 7.4) and then washed with SEM buffer. Samples were dehydrated in a graded ethanol series, dried using hexamethyldisilazane, sputter coated with gold and visualized with a JSM 6310 Scanning Electron Microscope at an acceleration voltage of 15 kV. Samples for TEM were fixed in 2% paraformaldehyde, 2.5% glutaraldehyde in 0.1 M cacodylate buffer over night at 4°C. After washing in cacodylate, specimen were postfixed in 1% OsO_4_ for 2 h on a shaker followed by washing steps and dehydration in a graded ethanol series. Aided by propylene oxide, samples were infiltrated in Epon812 and cured at 60°C for 2 days. 60 nm sections were cut with a Reichert Ultracut microtome and imaged with an FEI Tecnai20 transmission electron microscope. Micrographs were processed with Adobe Photoshop and analyzed with ImageJ freeware. For SEM, microvilli tips were automatically detected by the “find maxima” function of ImageJ and then corrected manually. Dots were assigned to each microvillus tip and the distances were calculated by the program using the “Nearest Neighbor Distance NND” plugin. For TEM images, microvilli length was measured manually using ImageJ. To quantify the area and circularity of microvilli cross‐sections, the “Gaussian Blur Filter” and “AutoThreshold” plugin were applied and corrected manually. The microvilli area and circularity were then calculated by ImageJ.

### 16S rRNA gene amplicon sequencing and data analysis

Timed matings were set up to ensure that all mice were of the same age. The offspring were genotyped and weaned at 3 weeks of age. At weaning, the offspring from different litters were mixed and 3 CDHR5^+/+^ and 3 CDHR5^∆/∆^ mice from different litters were caged together until they were 8 weeks old. Fecal pellets were collected from 8 week old adult mice. After pellet collection, adult females were remixed and 3 CDHR5^+/+^ and 3 CDHR5^∆/∆^ mice were housed together per cage. Adult male mice could not be mixed but were split to have 3 mice per cage (either 2 CDHR5^+/+^ and 1 CDHR5^∆/∆^ mice or 2 CDHR5^∆/∆^ and 1 CDHR5^+/+^ mice) to minimize cage effects. Mice were treated with DSS in sterile autoclaved drinking water (female mice 2.0%, male mice 1.5% DSS) for 7 days, followed by a 7‐day recovery period without DSS. Control mice received sterile autoclaved drinking water throughout the experiment. The weight and condition of the mice were monitored daily to ensure colitis. A second collection of fecal pellets was performed on day 14 after the recovery period and the pellets were immediately frozen in a preweighed Eppendorf tube on dry ice. Analysis of the gut microbiome was performed as described previously (Pereira *et al*, 2020). DNA extraction was performed with the QIAamp Fast Stool DNA Mini Kit (Qiagen) from fecal samples. 16S rRNA gene amplification PCR was performed using the 16S rRNA gene primers 515F and 806R that target most bacteria and archaea (Caporaso *et al*, 2011). Amplicons were amplified, barcoded, quantified, pooled, and sequenced at the Joint Microbiome Facility of the Medical University of Vienna and the University of Vienna (project ID JMF‐2112‐06) as described previously (Pjevac *et al*, 2021). Amplicon sequence variants (ASVs) were inferred using the DADA2 R package applying the recommended workflow (Callahan *et al*, 2016a, 2016b). FASTQ reads 1 and 2 were trimmed at 220 nucleotides and 150 nucleotides with allowed expected errors of 2, respectively. ASV sequences were subsequently classified using SINA version 1.6.1 and the SILVA database SSU Ref NR 99 release 138.1 using default parameters (Pruesse *et al*, 2012; Quast *et al*, 2013). Downstream analyses were performed using R v4.2 and Bioconductor v3.15 packages TreeSummarizedExperiment v2.4, mia v1.4 (https://github.com/microbiome/mia), vegan v2.6.2 (https://CRAN.R‐project.org/package=vegan), phyloseq v1.40, microbiome v1.18 (http://microbiome.github.io), microViz v0.9.2, DESeq2 v1.34 (McMurdie & Holmes, 2013; Love *et al*, 2014; Huang *et al*, 2020). Beta diversity was calculated by performing a PCoA with Aitchison distance using microViz. The difference in per‐group centroids was tested with a PERMANOVA on Aitchison distance using vegan and microViz. Pairwise differential abundance testing was performed using DESeq2 with alpha = 0.05 and otherwise default parameters after adding a pseudocount of 1 to the data.

### Quantitative PCR

RNA from tissues and cells was isolated using TRIzol Reagent (Thermo Fisher Scientific) and reverse transcribed using the QuantiTect Reverse Transcription Kit (Quiagen). For qPCR analysis the GoTaq qPCR Master Mix (Promega) and CFX96 Real‐Time System (Bio‐Rad) were used with the following mouse primers: 5′‐ACCATCCGTGTAGAGGTAGAA‐3′ and 5′‐GTCAAGGGGGCTTCCATAGT‐3′ for CDHR5 (product size 173 bp) and 5′‐GTCATCCCAGAGCTGAACGG‐3′ and 5′‐TACTTGGCAGGTTTCTCCAGG‐3′ for Gapdh (produce size 107 bp). Results were calculated using the ΔCt method. Relative quantification was achieved by normalizing to the expression values of the Gapdh gene.

### Glucose uptake assay

Mice were fasted for 6 h with water ad libitum. The baseline blood glucose levels were measured with 1 drop of blood, collected from the facial vein with a sterile lancet (VWR), using a CardioChek PLUS Analyzer and corresponding CardioCheck Glucose test strips (Exel Medical). A 10% glucose solution in sterile H_2_O (2 mg or 4 mg/g body weight) was administered orally with a 27‐gauge sterile needle. Blood glucose levels were monitored over 120 in 30 min intervals as described previously (Zhang *et al*, 2002).

### Alcian blue staining

The mucus layer and goblet cells in the intestine were stained with Alcian blue. Intestinal tissues were fixed in Carnoy's solution without flushing of the gut and embedded in paraffin. 4 μm sections were deparaffinized and stained with a 0.1% Alcian solution in 1% acetic acid and subsequently stained with nuclear fast red aluminum sulfate solution (N069.1, Roth).

### Isolation of intestinal epithelial cells

Small intestines were flushed with ice‐cold PBS and then inverted with a bent paper clip. Residual feces were removed by several washes with ice‐cold PBS and the cleaned inverted intestines were incubated in 5 ml of cell recovery solution (Corning) on ice in Petri dishes. After 45 min, intestines were transferred to Petri dishes containing 5 ml of ice‐cold PBS and the epithelial layers were gently scraped from the intestinal tissue with a microscope slide. The PBS containing the epithelial layers was transferred to a centrifugation tube, centrifuged at 500 *g* for 5 min and the pellets used for RNA isolation.

### Bulk RNA‐seq analysis

Total RNA from purified epithelial cells were extracted using TRIzol Reagent (Thermo Fisher Scientific) and processed for sequencing using the TruSeq RNA Sample Preparation Kit (Illumina) according to the manufacturer's protocol. mRNA was purified using poly(T)‐oligo‐attached magnetic beads, fragmented, and applied to first‐strand complementary DNA (cDNA) synthesis. Second‐strand cDNA synthesis was performed using DNA polymerase I and RNase H. cDNA was end‐repaired, A‐tailed, ligated to adapters, and amplified to create the final cDNA library for sequencing (HiSeq2000, Illumina). TopHat2 algorithm was used to align raw RNA‐seq data to mm10 (Kim *et al*, 2013). Differentially expressed genes were identified using DeSeq2 algorithm (Love *et al*, 2014). An adjusted *P* < 0.005 and a fold change > 2 or < −2 was defined as cut‐off for differentially expressed genes. Gene Ontology enrichment analyses were performed using GOrilla software (Eden *et al*, 2007; Love *et al*, 2014).

### Microarray data availability and analysis

Microarray data were analyzed using the freely available online tool GEO2R (https://www.ncbi.nlm.nih.gov/geo/info/geo2r.html) to identify differentially expressed genes in different conditions. Differential gene expression levels were identified by Bioconductor package DESeq2 using a Wald test that shrinks estimates of LFC divided by its standard error resulting in a z‐statistic which is compared to a standard normal distribution. In DESeq2, the *P*‐values attained by the Wald test were corrected for multiple testing using the Benjamini and Hochberg method by default.

### scRNA‐seq data availability and differential gene expression analysis

scRNA sequencing data as reported by Haber *et al* (2017) and Smillie *et al* (2019) were accessed from the Single Cell Portal (https://portals.broadinstitute.org/single_cell) under the accession numbers GSE92332 and SCP259, respectively (Haber *et al*, 2017; Smillie *et al*, 2019). Provided Seurat object included raw counts, normalized data and pca of the discovery cohort of 17 patients (10 healthy and seven with ulcerative colitis), with matched inflamed and uninflamed samples. scRNA‐sequencing analysis was carried out using the most recent version of Seurat functions (Seurat V4.1.1; https://satijalab.org/seurat/; Hao *et al*, 2021). Differential gene expression analysis between healthy controls and inflamed tissue or inflamed tissue and uninflamed tissue were estimated through the default Wilcoxon Rank Sum test of the Seurat “FindMarkers” function. *P* value adjustment for multiple testing was corrected using Bonferroni correction based on total number of genes in the respective comparison. All processing was performed in R version 4.1.3 (https://www.r‐project.org).

### Western blot

Western blot was performed as previously described (Svinka *et al*, 2017) with the same antibodies used for immunofluorescence staining.

### Statistics

The normality of the data distribution was tested by Kolmogorov–Smirnov or D'Agostino‐Pearson and statistical tests were performed accordingly. Comparisons of two groups were calculated with unpaired Student's *t*‐test or Mann–Whitney *U* test. For more than two groups one‐way Analysis of Variance (ANOVA) and Tukey's multiple comparison test, Bonferroni's *post‐hoc* test or the Kruskal–Wallis test and Dunn's *post‐hoc* test were used. Significance of differences in weight loss and protein expression of apical markers were calculated by performing an area under the curve (AUC) calculation and testing the AUC by independent *t*‐test. All the analyses were performed using GraphPad Prism 5 software. Significant differences between experimental groups were stated as: *P < 0.05, **P < 0.01, ***P < 0.001 or ****P < 0.0001.

## Author contributions

**Bernadette Mödl:** Methodology. **Monira Awad:** Methodology. **Daniela Zwolanek:** Methodology. **Irene Scharf:** Methodology. **Katharina Schwertner:** Methodology. **Danijela Milovanovic:** Methodology. **Doris Moser:** Methodology. **Katy Schmidt:** Methodology. **Petra Pjevac:** Methodology. **Bela Hausmann:** Methodology. **Dana Krauß:** Investigation. **Thomas Mohr:** Investigation. **Jasmin Svinka:** Methodology. **Lukas Kenner:** Investigation. **Emilio Casanova:** Investigation. **Gerald Timelthaler:** Methodology. **Maria Sibilia:** Investigation. **Sigurd Krieger:** Investigation. **Robert Eferl:** Conceptualization.

## Disclosure and competing interests statement

The authors declare that they have no conflict of interest.

## Supporting information



Appendix

Expanded View Figures PDF

PDF+

Source Data for Figure 1

Source Data for Figure 2

Source Data for Figure 3

Source Data for Figure 4

Source Data for Figure 5

## Data Availability

16S rRNA gene amplicon sequencing data are available in the BioProject database under BioProject ID PRJNA887949 (https://www.ncbi.nlm.nih.gov/bioproject/PRJNA887949). The RNA sequencing data are available under the accession number E‐MTAB‐12525 in ArrayExpress. Source data regarding microscopical images are available in the Biostudies database under the accession number S‐BIAD832 (https://www.ebi.ac.uk/biostudies/bioimages/studies/S‐BIAD832).
