# Supplementary material for: Defects in microvillus crosslinking sensitize to colitis and inflammatory bowel disease
Source: EMBO Rep. 2023 Sep 11;24(10):e57084. doi: 10.15252/embr.202357084 (PMC10561180; doi:10.15252/embr.202357084)
Supplement: Supplementary file 1 — Appendix [file EMBR-24-e57084-s003.pdf]

APPENDIX

TABLE OF CONTENTS

APPENDIX FIGURE S1	2
APPENDIX FIGURE S2	4
APPENDIX FIGURE S3	6
APPENDIX FIGURE S4	8
APPENDIX FIGURE S5	9
APPENDIX FIGURE S6	10
APPENDIX FIGURE S7	11
APPENDIX FIGURE S8	12
APPENDIX FIGURE S9	14

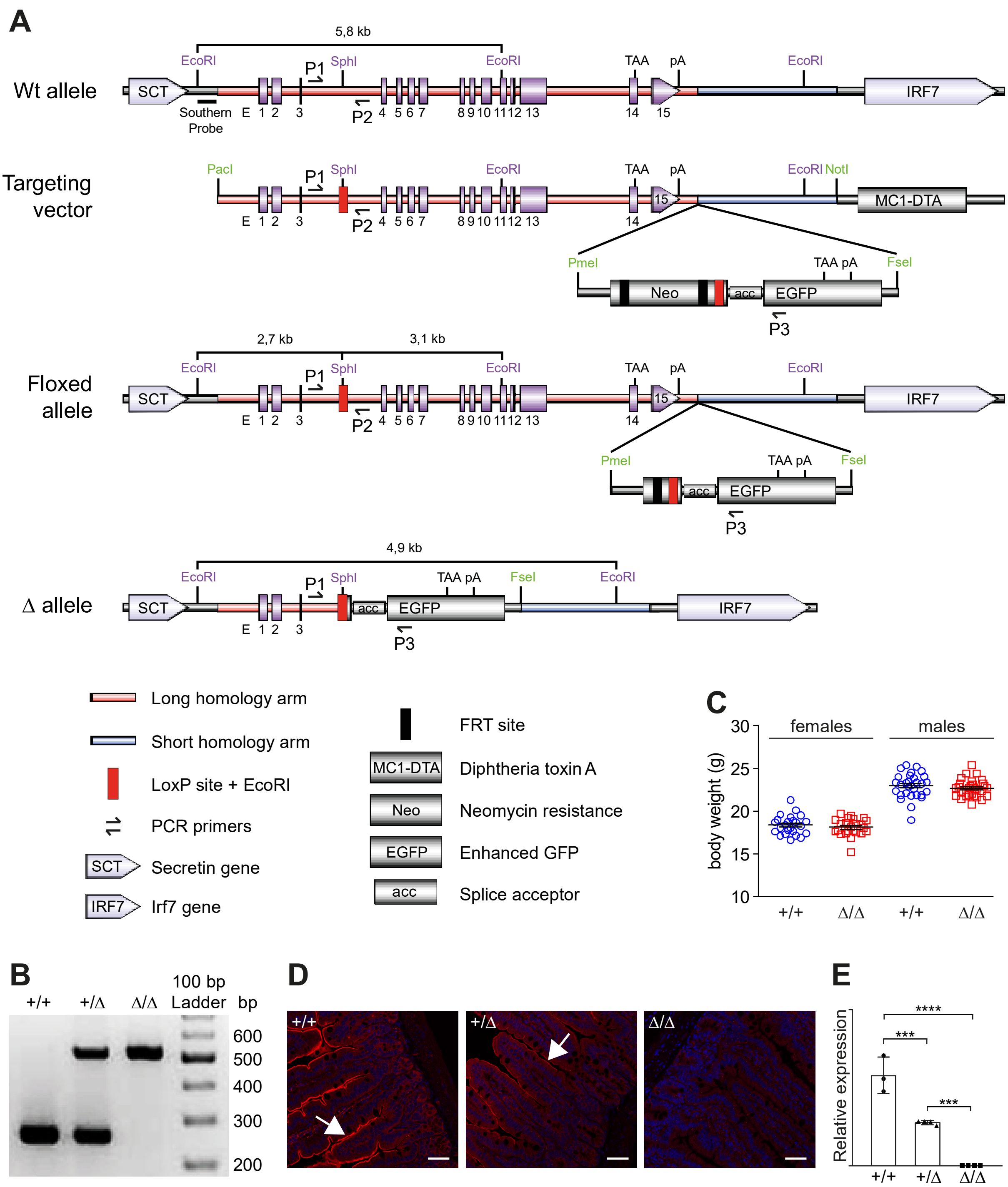

Appendix Figure S1: Generation of CDHR5$^{\Delta/\Delta}$ mice. (**A**) Two loxP sites, flanking exons 4-15 of CDHR5 and the polyadenylation signal, were introduced into the genomic CDHR5 locus (wt allele) of ES cells by homologous recombination with a targeting vector. The floxed locus was deleted with ß-actin-Cre germline deleter mice resulting in a null (Δ) allele with an in-frame fusion of truncated CDHR5 and EGFP. (**B**) Genotyping PCR using primers P1, P2, and P3 (location shown in A) with tail DNA from CDHR5$^{+/+}$, CDHR5$^{+/\Delta}$ and CDHR5$^{\Delta/\Delta}$ mice. (**C**) Scatter plot for body weight of 8-week-old adult female and male CDHR5$^{+/+}$ and CDHR5$^{\Delta/\Delta}$ mice. (**D**) Immunofluorescence for CDHR5 in the duodenum of mice (CDHR5 in red and indicated by arrows, nuclei in blue). Scale bar = 50 µm. (**E**) qPCR for CDHR5 mRNA expression in intestinal epithelial cells of indicated genotypes. Data information: For C, statistical analysis was performed using unpaired Student's t-test for each gender. Differences

are not significant. For E, data were normalized for expression of GAPDH. Bars represent average of data +/- SEM (≥ 3 mice per genotype). Statistical analysis was performed using one-way ANOVA and Tukey’s multiple comparison test. ***$p < 0.001$ or ****$p < 0.0001$.

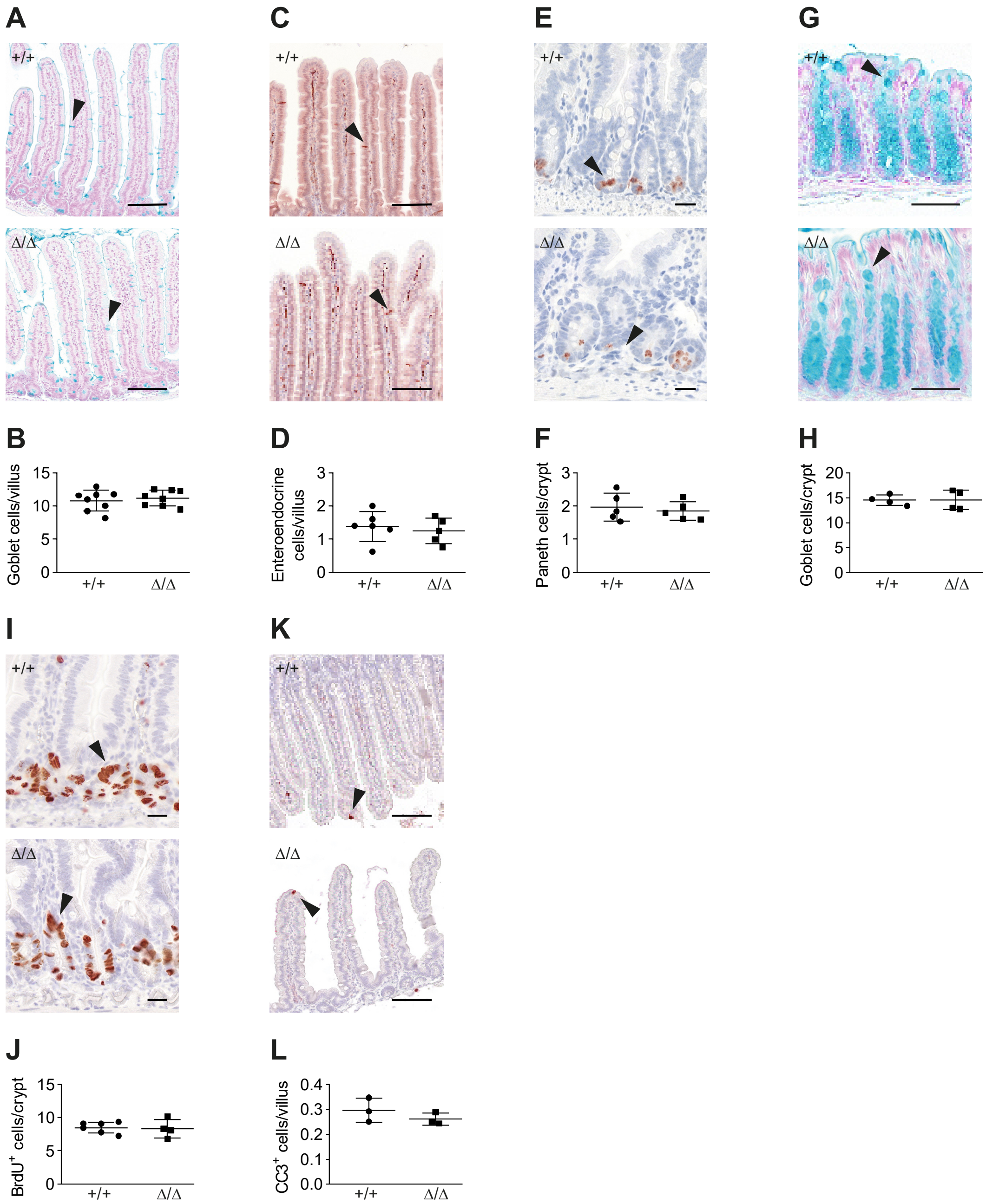

Appendix Figure S2: Characterization of intestinal cell types in CDHR5$^{\Delta/\Delta}$ mice. (**A**) Immunohistochemical synaptophysin staining of duodenal villi from CDHR5$^{+/+}$ (upper panel) and CDHR5$^{\Delta/\Delta}$ (lower panel) mice showing enteroendocrine cells (arrowheads). Scale bar = 100 µm. (**B**) Scatter plot showing the number of enteroendocrine cells per villus. (**C**) Immunohistochemical lysozyme staining of duodenal crypts from CDHR5$^{+/+}$ (upper panel) and CDHR5$^{\Delta/\Delta}$ (lower panel) mice showing Paneth cells (arrowheads). Scale bar = 20 µm. (**D**) Scatter plot showing the number of Paneth cells per crypt. (**E**) Immunohistochemical BrdU staining of duodenal crypts from CDHR5$^{+/+}$ (upper panel) and CDHR5$^{\Delta/\Delta}$ (lower panel) mice showing proliferating cells (arrowheads). Scale bar = 20 µm. (**F**) Scatter plot showing the number of proliferating cells per crypt. (**G**) Alcian blue staining of duodenal villi from CDHR5$^{+/+}$ (upper panel) and CDHR5$^{\Delta/\Delta}$ (lower panel) mice showing goblet cells (arrowheads). Scale bar = 100 µm. (**H**) Scatter plot showing the number of goblet cells per villus. (**I**) Alcian blue staining of colon sections from CDHR5$^{+/+}$ (upper panel) and CDHR5$^{\Delta/\Delta}$ (lower panel) mice showing goblet cells (arrowheads). Scale bar = 50 µm. (**J**) Scatter plot showing the

number of positive cells per crypt. (**K**) Immunohistochemical staining for cleaved caspase 3 of duodenal villi from CDHR5$^{+/+}$ (upper panel) and CDHR5$^{\Delta/\Delta}$ (lower panel) mice showing apoptotic cells (arrowheads). Scale bar = 20 µm. (**L**) Scatter plot showing the number of apoptotic cells per villus. Data information: Scatter plots represent average of data +/- SEM. Each data point represents average of ≥10 villi per mouse. All statistical analyses were performed using unpaired Student's t-test. Differences are not significant.

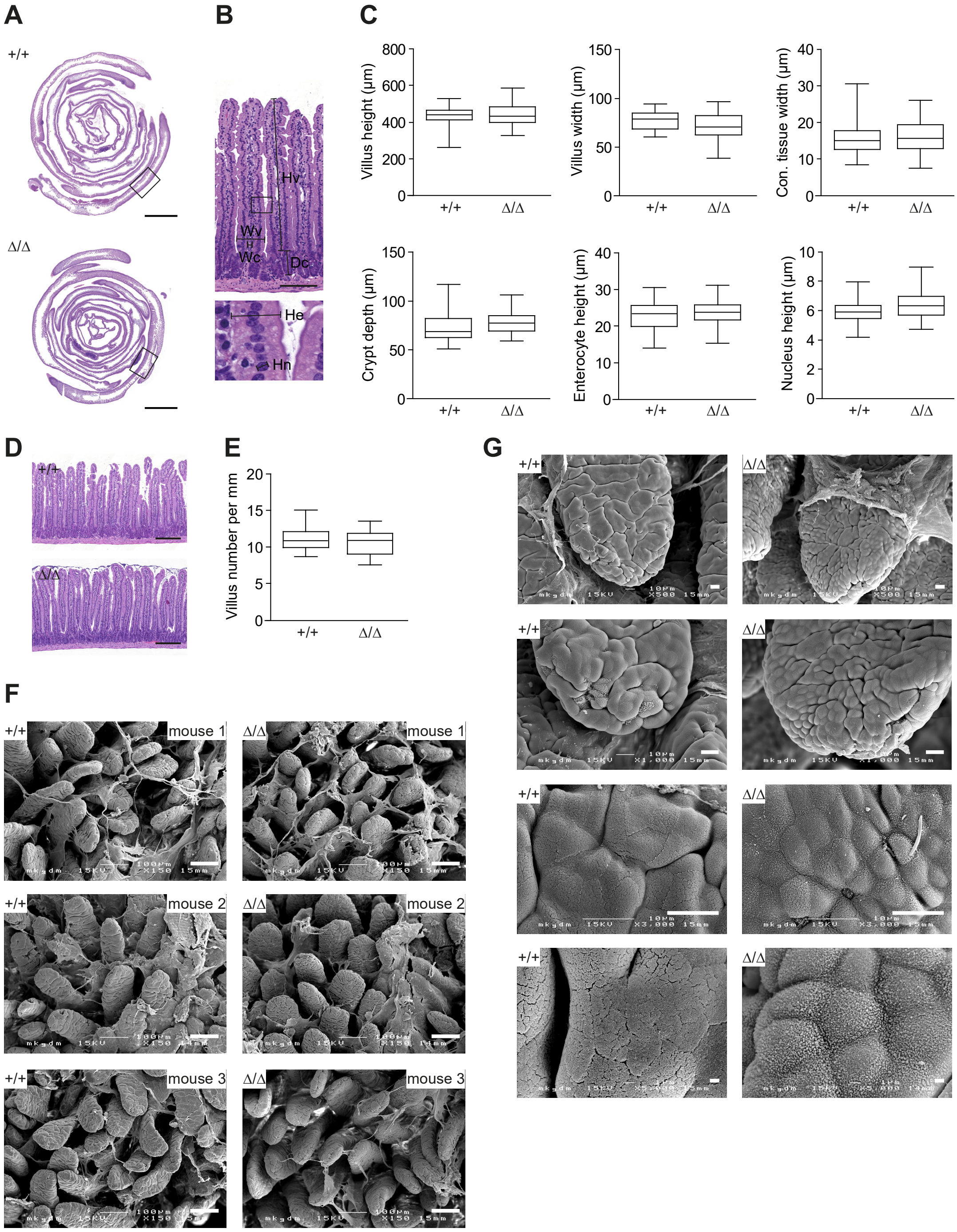

Appendix Figure S3: Villus morphology and number are not affected in CDHR5$^{\Delta/\Delta}$ mice. (**A**) H&E-stained Swiss rolls of CDHR5$^{+/+}$ and CDHR5$^{\Delta/\Delta}$ mice. Rectangles indicate regions for analysis of villus morphology and number. Scale bar = 5 mm. (**B**) Higher magnifications of villi in the duodenum with indicated parameters for histomorphometric analysis. The position of the lower image (close up) is indicated by the rectangle in the upper image. Scale bar = 100 µm. Hv: villus height, Wv: villus width, Wc: connective tissue width, Dc: crypt depth, He: enterocyte height, Hn: height of enterocyte nuclei. (**C**) Box plots with quantitative values of villus parameters in the duodenum. (**D**) H&E staining of duodenal villi in CDHR5$^{+/+}$ and CDHR5$^{\Delta/\Delta}$ mice. The same duodenal region as in B is shown for CDHR5$^{+/+}$ mice. Scale bar = 200 µm. (**E**) Box plots with quantitative values of villus numbers. (**F**) Scanning electron microscopy of villi in the duodenum of three CDHR5$^{+/+}$ and CDHR5$^{\Delta/\Delta}$ mice showed no gaps

between villi. Scale bar = 100 µm. (**G**) SEM images of duodenal villi in CDHR5$^{+/+}$ and CDHR5$^{\Delta/\Delta}$ mice with increasing magnification (top to bottom) revealed disorganization of the brush border in CDHR5$^{\Delta/\Delta}$ mice. Scale bar = 10 µm, 10 µm, 10 µm and 1 µm from top to bottom. Data information: All box plots were generated with data from 4 mice per genotype and 10 villi per mouse. Statistical analyses were performed using unpaired Student's t-test. Differences are not significant.

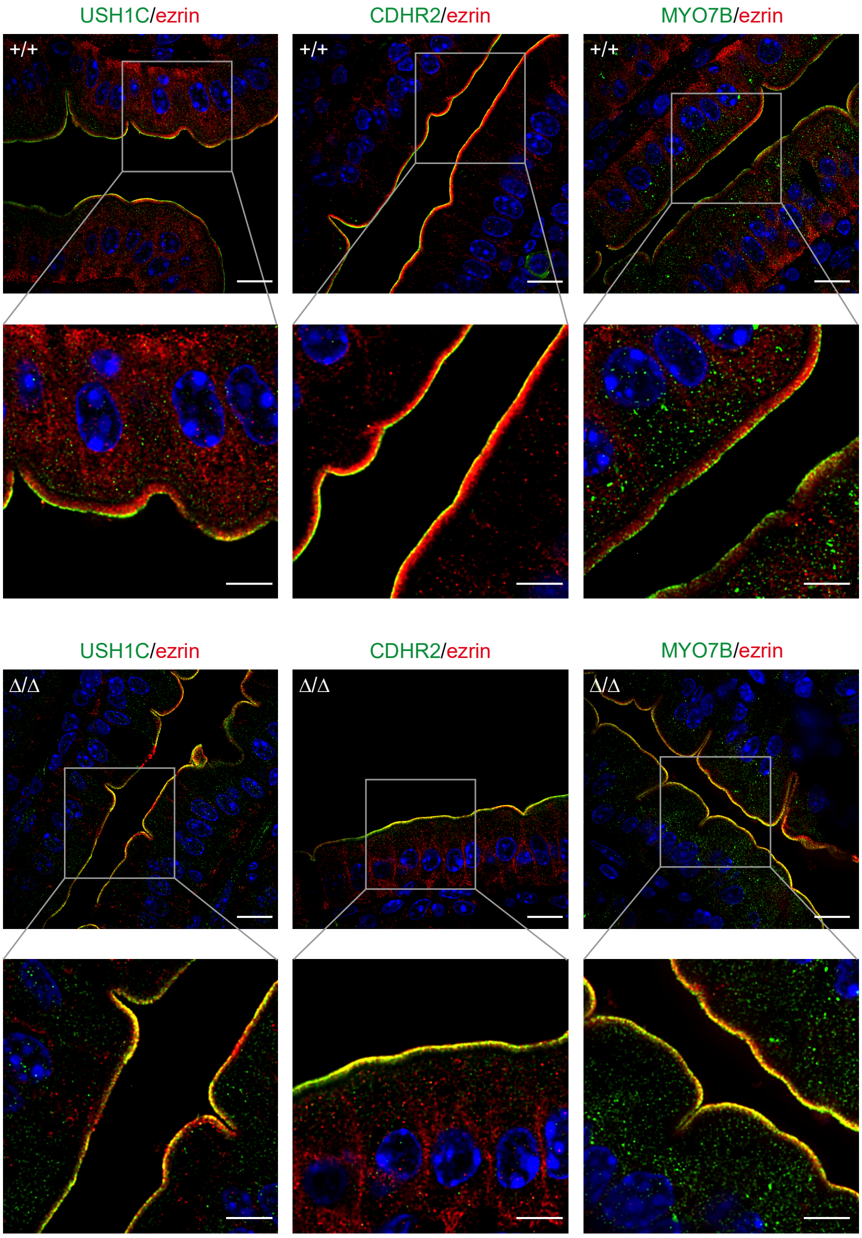

Appendix Figure S4: Loss of microvillus IMAC tip localization in CDHR5$^{\Delta/\Delta}$ mice. Spinning disc immunofluorescence images of duodenal brush borders of CDHR5$^{+/+}$ and CDHR5$^{\Delta/\Delta}$ mice stained for USH1C/ezrin, CDHR2/ezrin or MYO7B/ezrin. USH1C, CDHR2 and MYO7B in green, ezrin in red, nuclei in blue. Scale bars = 10 µm of low magnification images and 5 µm of high magnification images. The positions of the high magnification images are indicated by the rectangles.

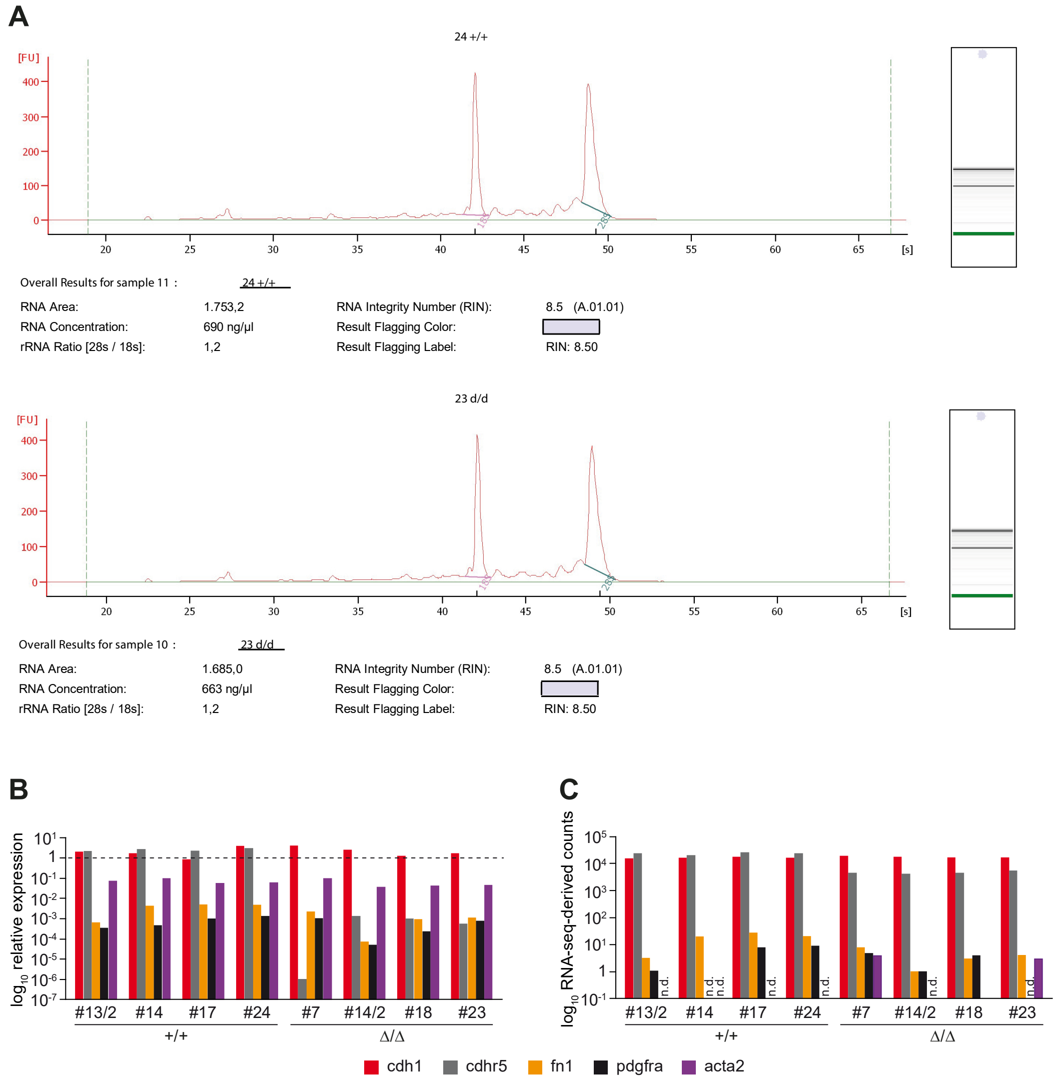

Appendix Figure S5: Quality analysis of intestinal epithelial cells. Intestinal epithelial cells were isolated from CDHR5$^{+/+}$ and CDHR5$^{\Delta/\Delta}$ mice (4 mice per genotype) for RNA-seq. (**A**) An Agilent 2100 Bioanalyzer was used to assess the RIN values for RNA integrity in total RNA extracts from epithelial cell preparations. Two representative examples from CDHR5$^{+/+}$ mice (top electropherogram) and CDHR5$^{\Delta/\Delta}$ mice (bottom electropherogram) are shown. (**B**) The purity of isolated epithelial cells was confirmed by TaqMan qPCR for epithelial (CDH1, CDHR5) and lamina propria (FN1, PDGFRA, ACTA2) markers. From each mouse, RNA was isolated from purified intestinal epithelial cells and a separate piece of whole intestinal tissue. Marker expression in epithelial preparations was normalized for expression in the corresponding whole gut, meaning that expression values of all markers in the whole gut were 1 (indicated by the dotted line). (**C**) Expression of epithelial and lamina propria markers were extracted from RNA-seq data of epithelial cells. Note that hardly any RNA-seq-derived counts were obtained for lamina propria markers. Sample identifications in B and C are identical with sample identifications of RNA-seq data deposited at ArrayExpress (E-MTAB-12525). Data information: statistical analysis was not performed for data in B and C.

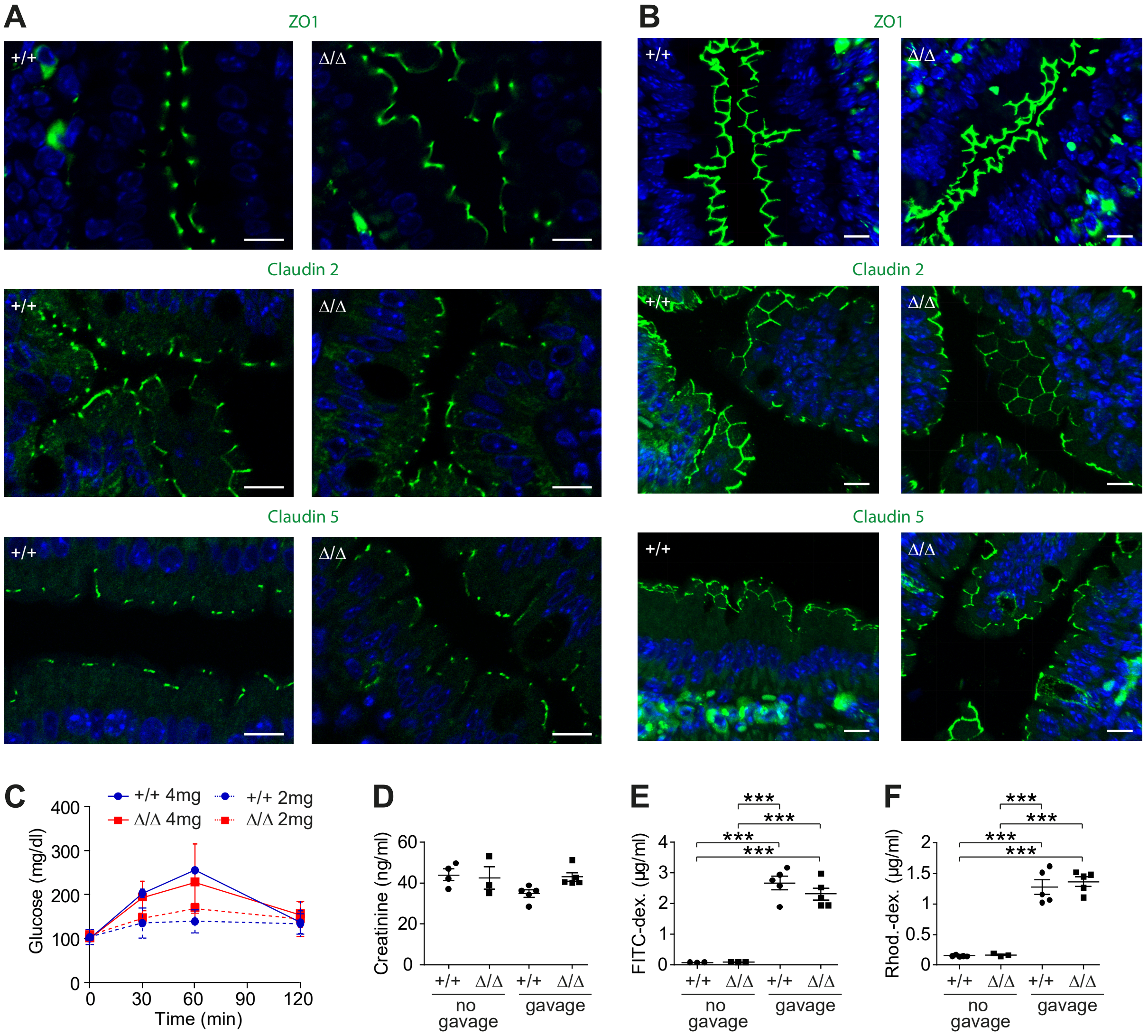

Appendix Figure S6: The paracellular pathway is not affected in CDHR5$^{\Delta/\Delta}$ mice. (**A**) High magnification confocal immunofluorescence images for ZO-1, claudin-2 and claudin-5 expression in the small intestine of CDHR5$^{+/+}$ and CDHR5$^{\Delta/\Delta}$ mice. Green dots indicate cross-sectioned tight junctions. Nuclei in blue. Scale bar = 10 µm. (**B**) High magnification 3D reconstruction of ZO-1-positive, claudin-2-positive and claudin-5-positive ring-like tight junctions using serial confocal immunofluorescence images. Scale bar = 10 µm. (**C**) Kinetics of serum glucose levels after oral gavage (n ≥ 5 mice per genotype and experiment). (**D-F**) Serum creatinine (D), 4 kDa FITC-dextran (E) and 70 kDa Rhodamine-dextran levels (F) without and after oral gavage. Data information: For C, the AUC was calculated for 2mg and 4mg gavage experiments separately. Statistical analyses were performed using unpaired Student's t-test. Differences are not significant. Scatter plots D-F represent average of data +/- SEM (≥ 3 mice per genotype). Statistical analyses were performed using one-way ANOVA and Tukey's multiple comparison test.

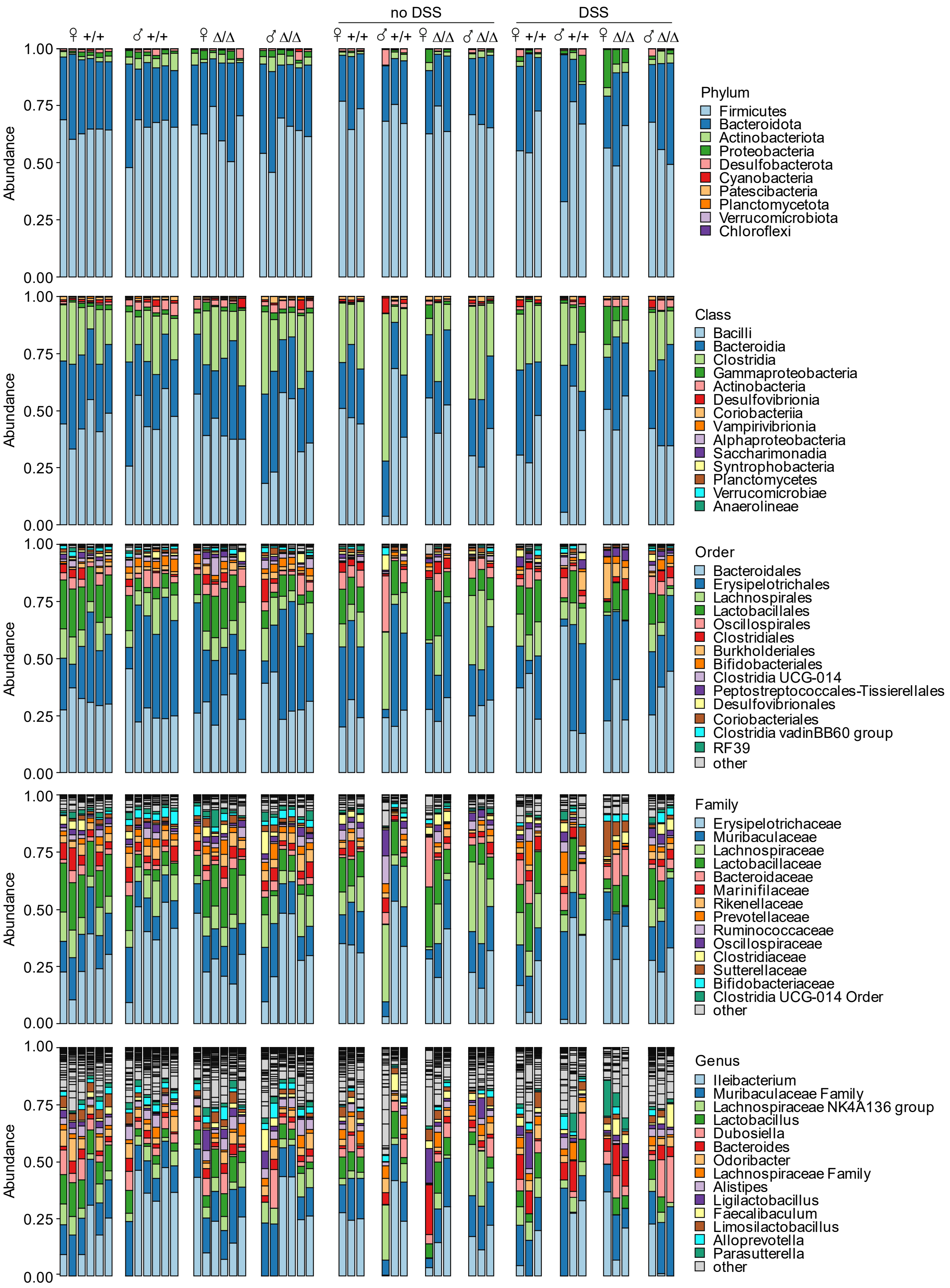

Appendix Figure S7: Microbiome composition in untreated and DSS-treated CDHR5$^{+/+}$ and CDHR5$^{\Delta/\Delta}$ mice. Stacked bar plots for taxonomic ranks phylum, class, order, family, and genus are shown.

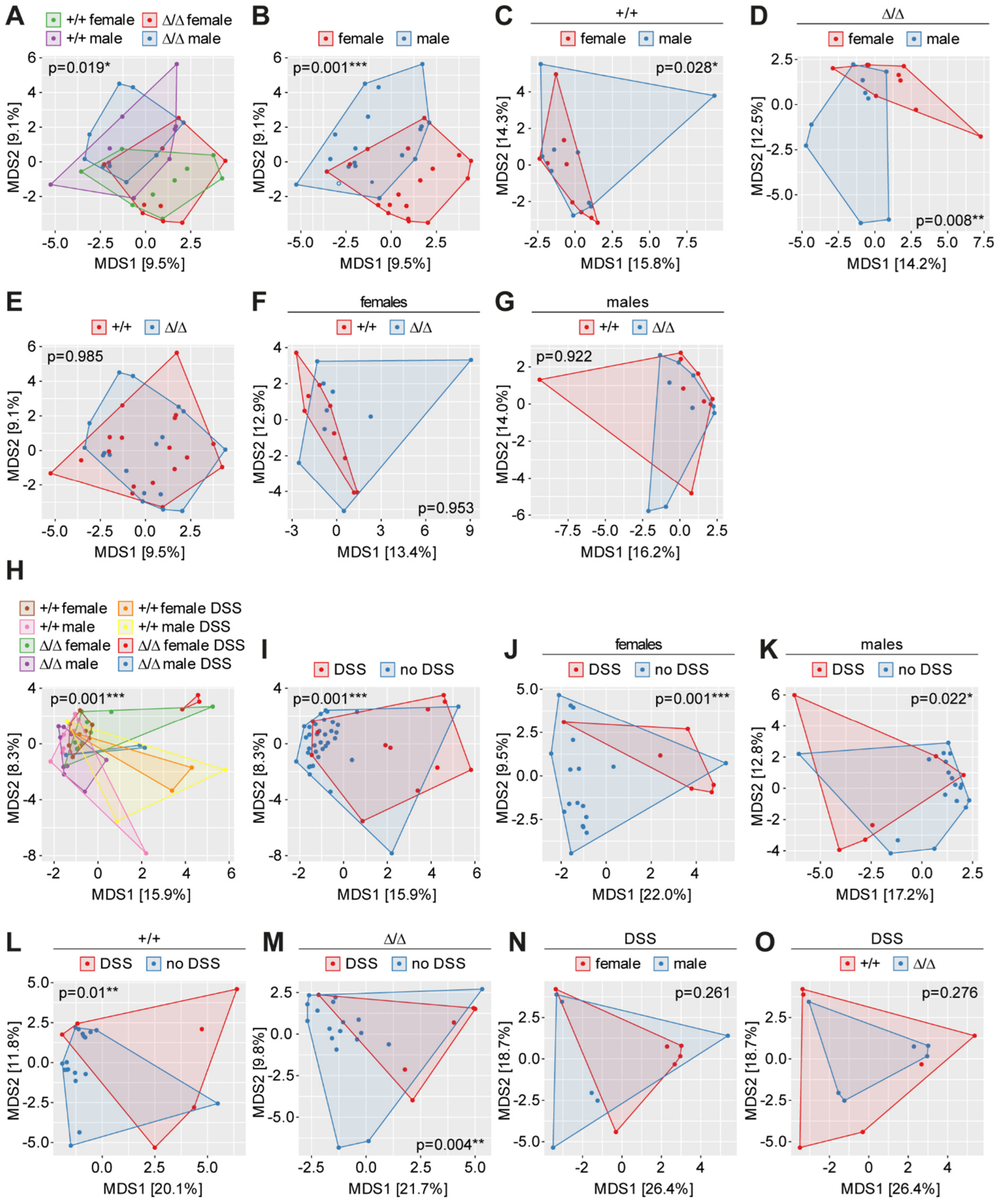

Appendix Figure S8: Principal component analysis (PCoA) of the microbiome composition in untreated and DSS-treated mice with or without sex and/or genotype stratification. (**A**) Principal component analysis (PCoA) of the microbiome composition of female and male CDHR5$^{+/+}$ and CDHR5$^{\Delta/\Delta}$ mice. (**B**) PCoA of female and male mice without genotype stratification. (**C**) PCoA of female and male CDHR5$^{+/+}$ mice. (**D**) PCoA of female and male CDHR5$^{\Delta/\Delta}$ mice. (**E**) PCoA of CDHR5$^{+/+}$ and CDHR5$^{\Delta/\Delta}$ mice without sex stratification. (**F**) PCoA of female CDHR5$^{+/+}$ and CDHR5$^{\Delta/\Delta}$ mice. (**G**) PCoA of male CDHR5$^{+/+}$ and CDHR5$^{\Delta/\Delta}$ mice. (**H**) PCoA of female and male CDHR5$^{+/+}$ and CDHR5$^{\Delta/\Delta}$ mice with and without DSS treatment. (**I**) PCoA of untreated and DSS-treated mice without sex and

genotype stratification. (**J**) PCoA of untreated and DSS-treated female mice without genotype stratification. (**K**) PCoA of untreated and DSS-treated male mice without genotype stratification. (**L**) PCoA of untreated and DSS-treated CDHR5$^{+/+}$ mice without sex stratification. (**M**) PCoA of untreated and DSS-treated CDHR5$^{\Delta/\Delta}$ mice without sex stratification. (**N**) PCoA of DSS-treated male and female mice without genotype stratification. (**O**) PCoA of DSS-treated CDHR5$^{+/+}$ and CDHR5$^{\Delta/\Delta}$ mice without sex stratification. Data information: microbiome data of fecal samples from 9 untreated and 3 DSS-treated mice per sex and genotype (total 48 mice) were used for PCoA. PERMANOVA p values are indicated.

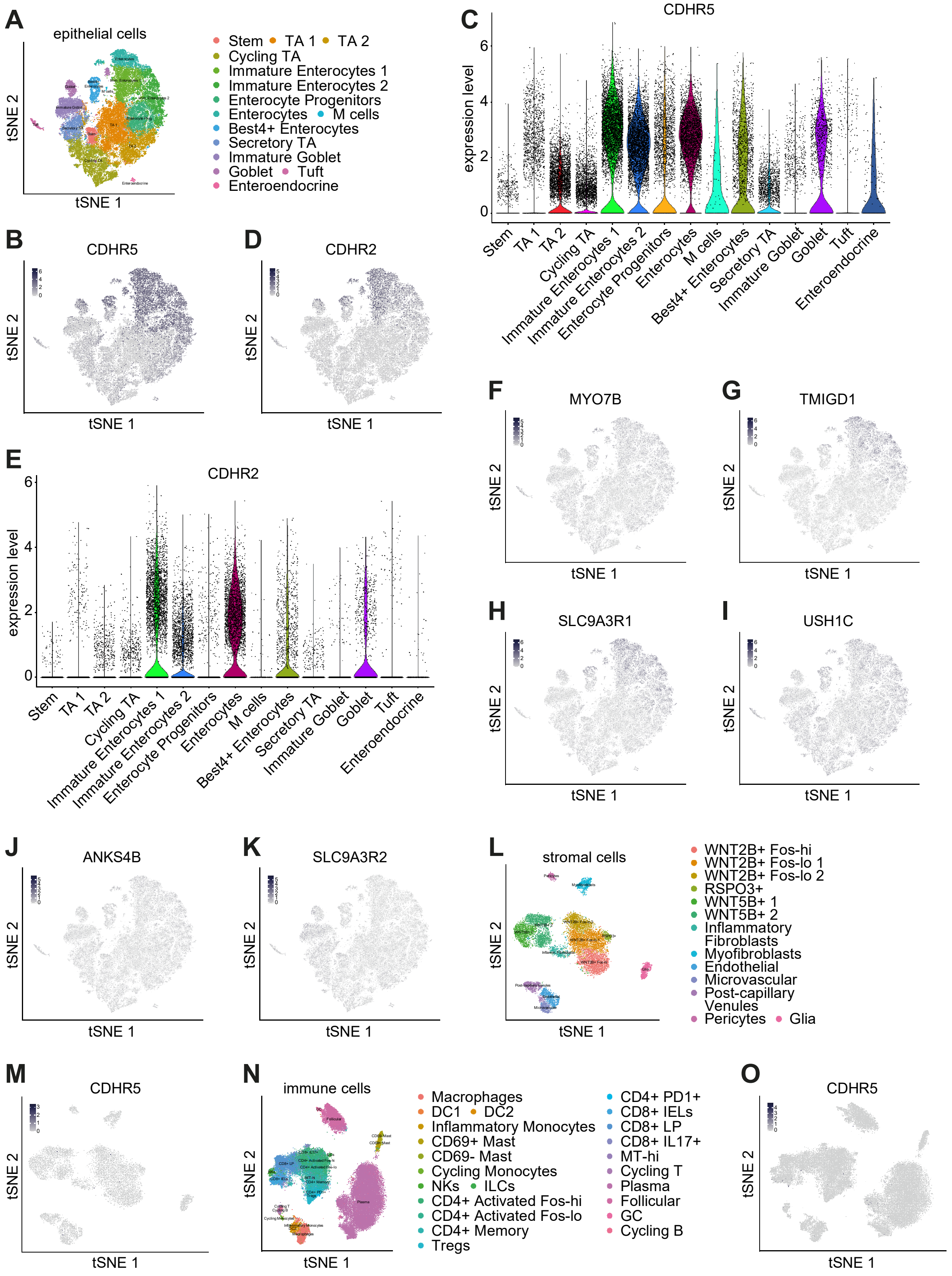

Appendix Figure S9: CDHR5 is expressed in enterocytes of diseased gut areas of UC patients but not in immune cells or stromal cells. (**A**) tSNE plot of the total epithelial cell population of healthy individuals and UC patients. (**B**) tSNE plots of the total epithelial cell population indicating the expression of CDHR5. (**C**) Violin plot for expression of CDHR5 in

epithelial cells from healthy individuals as well as epithelial cells from inflamed or non-inflamed gut areas of UC patients. (**D**) tSNE plot of the total epithelial cell population indicating the expression of CDHR2. (**E**) Violin plot for expression of CDHR2 in epithelial cells from healthy individuals as well as epithelial cells from inflamed or non-inflamed gut areas of UC patients. (**F-K**) tSNE plots of the total epithelial cell population indicating the expression of IMAC components. (**L**) tSNE plot of the total stromal cell population of healthy individuals and UC patients. (**M**) tSNE plot of the total stromal cell population indicating the expression of CDHR5. **(N**) tSNE plot of the total immune cell population of healthy individuals and UC patients. (**O**) tSNE plot of the total immune cell population indicating the expression of CDHR5. For all data, the scRNA-seq dataset of Smillie was used (for reference see main manuscript).
