# Supplementary material for: Defects in microvillus crosslinking sensitize to colitis and inflammatory bowel disease
Source: EMBO Rep. 2023 Sep 11;24(10):e57084. doi: 10.15252/embr.202357084 (PMC10561180; doi:10.15252/embr.202357084)
Supplement: Supplementary file 2 — Expanded View Figures PDF [file EMBR-24-e57084-s009.pdf]

Expanded View Figures

Figure EV1. CDHR5 is mainly expressed by differentiated intestinal epithelial cells.

A tSNE plot of adult mouse intestinal epithelial cells.
B tSNE plot of adult mouse intestinal epithelial cells indicating the expression of CDHR5.
C Violin plots for expression of CDHR5 in individual adult mouse intestinal epithelial cell lineages. For (A–C) the scRNA-seq dataset of Haber was used (Haber *et al*, 2017).
D High-magnification immunofluorescence images of duodenal epithelial cells stained for IAP. The length of the fluorescence signal (green) in the brush border was measured with the ZEN software. Two measurements per genotype are indicated. Note the reduced length of the fluorescence signal in CDHR5$^{\Delta/\Delta}$ mice. Scale bar = 5 µm.
E Scatter plots showing the length of fluorescence signals for DPPIV, IAP, NHE3, p-ERM and ezrin on fluorescent images of duodenal epithelial cells from CDHR5$^{+/+}$ and CDHR5$^{\Delta/\Delta}$ mice (see Fig 2E). Length measurements were performed with ZEN software as shown in (D).
F Normalization of the area under the curve values (see Fig 2F) to the reduced length of the fluorescence signals eliminated the differences in relative protein levels of DPPIV, IAP, NHE3, p-ERM, and ezrin between CDHR5$^{+/+}$ and CDHR5$^{\Delta/\Delta}$ mice.
G Western blot for apical markers IAP, NHE3, and ezrin. Protein was isolated from purified intestinal epithelial cells of CDHR5$^{+/+}$ and CDHR5$^{\Delta/\Delta}$ mice. Actin was used as loading control.

Data information: Scatter plots in (E and F) represent mean ± SEM (3 mice per genotype and 2 duodenal regions per mouse were analyzed). Each data point in (E) represents the mean of 5 length measurements per region. Statistical analyses were performed using unpaired Student's *t*-test. Differences in (F) are not significant. ***$P < 0.001$.

© 2023 The Authors

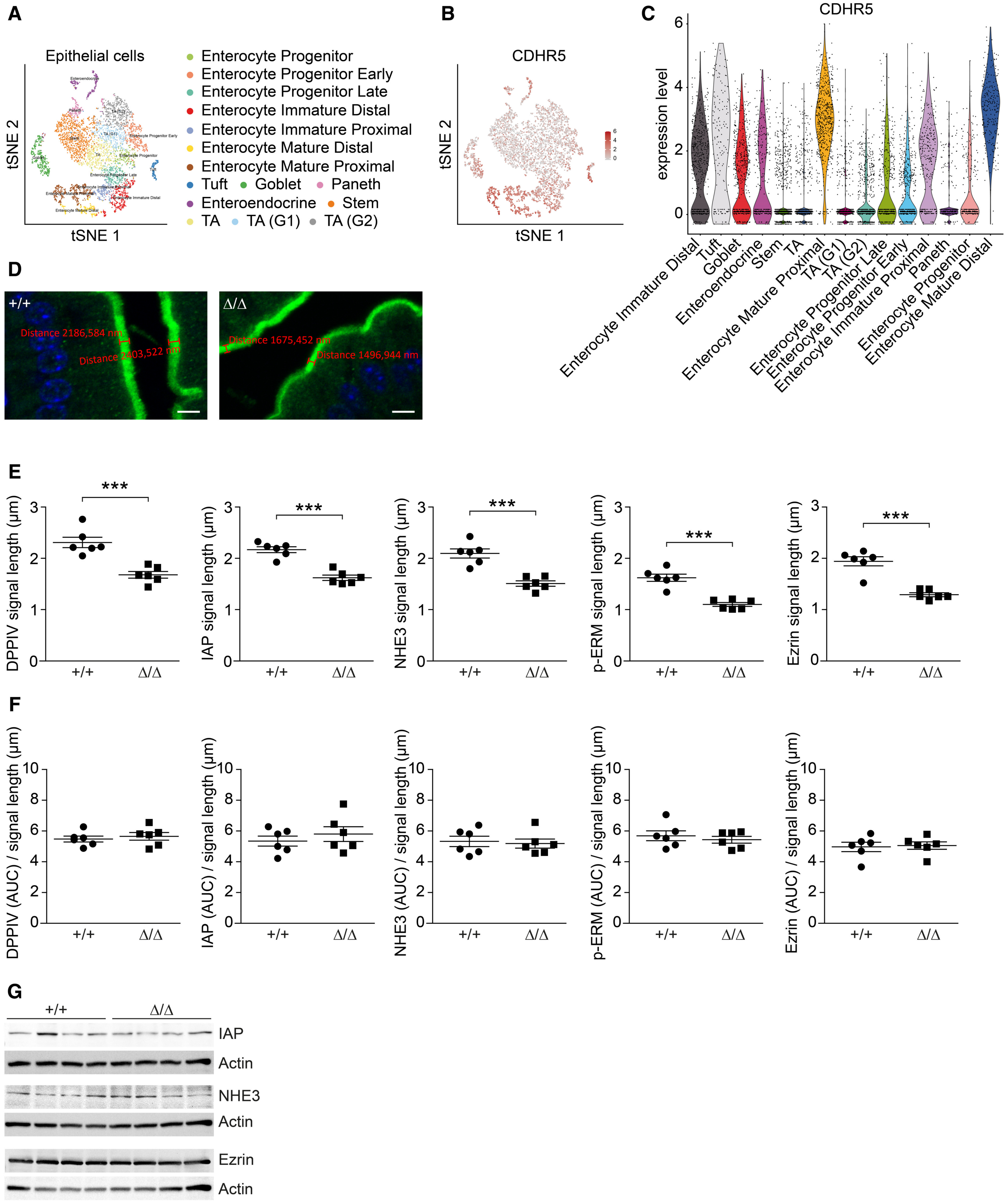

Figure EV1.

 © 2023 The Authors

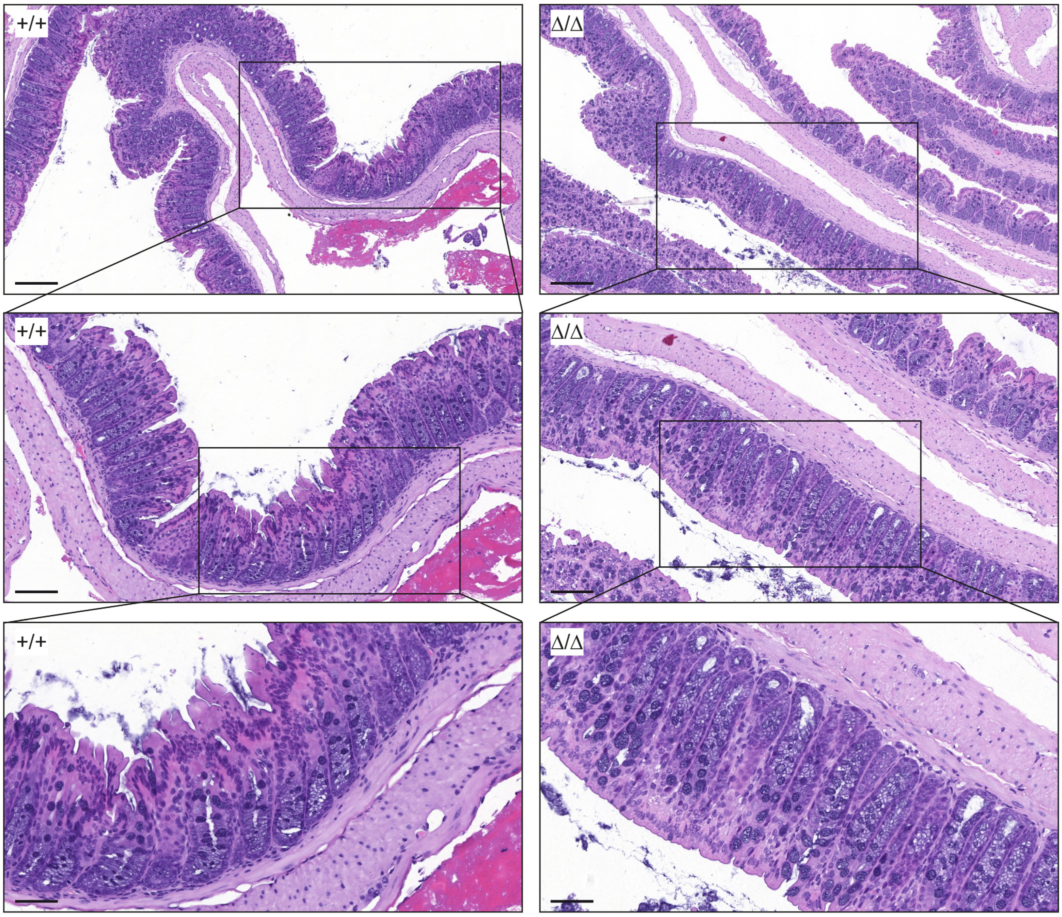

Figure EV2. CDHR5$^{\Delta/\Delta}$ mice do not develop spontaneous colitis.

Swiss rolls of colon tissue from CDHR5$^{+/+}$ and CDHR5$^{\Delta/\Delta}$ mice were stained with H&E. Images with increasing magnification (top to bottom) showed no evidence of spontaneous colitis or immune infiltration in CDHR5$^{\Delta/\Delta}$ mice. Scale bars = 200, 100, and 50 μm from top to bottom images, respectively.

© 2023 The Authors

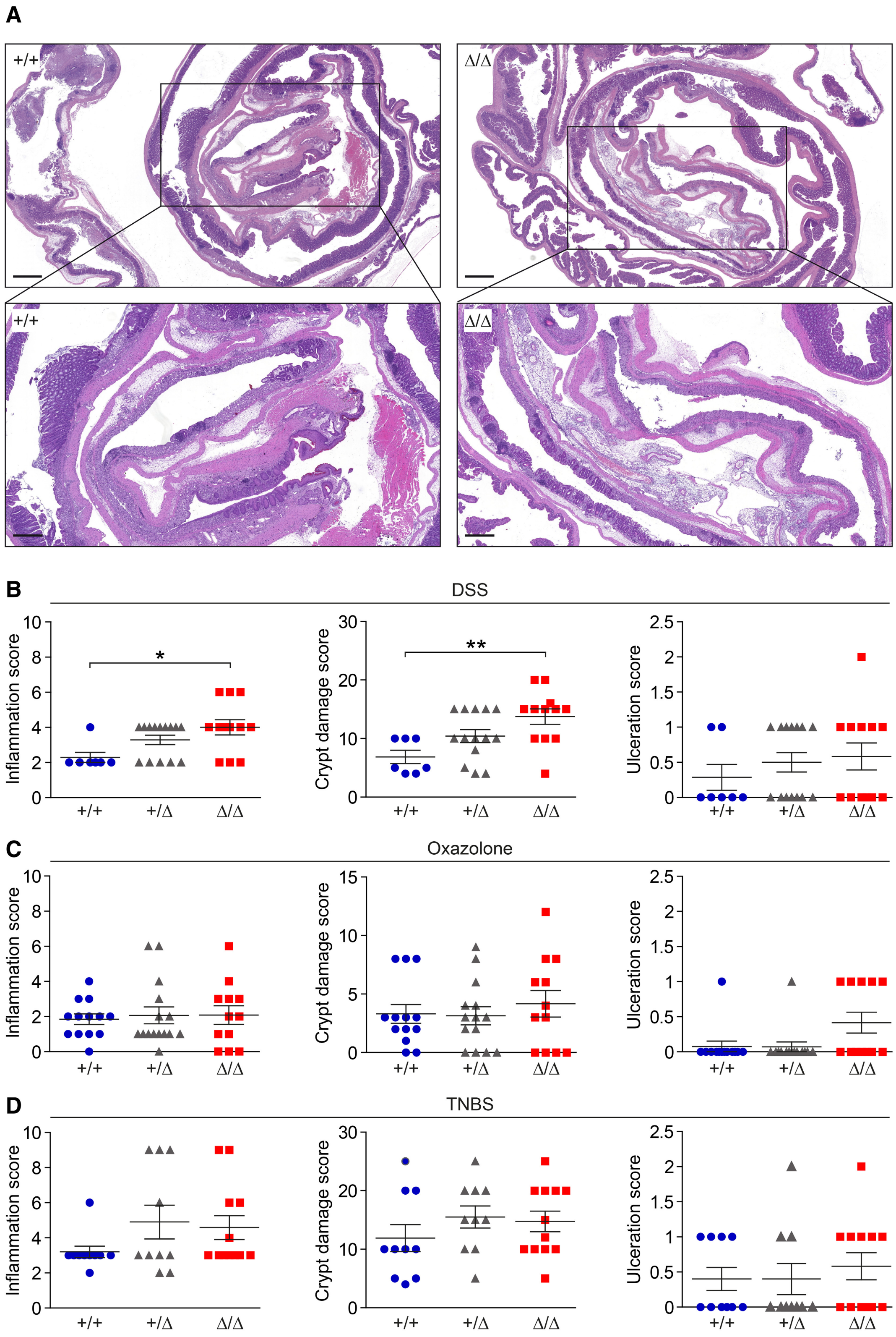

Figure EV3.

 © 2023 The Authors

◀ **Figure EV3. Inflammation and crypt damages scores are increased in CDHR5$^{\Delta/\Delta}$ mice with DSS-induced colitis.**

A Low magnification H&E-stained Swiss rolls of CDHR5$^{+/+}$ and CDHR5$^{\Delta/\Delta}$ mice suffering from acute DSS-induced colitis. Scale bars = 1 mm of upper images and 500 μm of lower images. The positions of the lower images are indicated by the rectangles.

B–D Inflammation, crypt damage, and ulcerations scores of CDHR5$^{+/+}$, CDHR5$^{+/\Delta}$ and CDHR5$^{\Delta/\Delta}$ mice with DSS (B), oxazolone (C) or TNBS (D) colitis. Scatter plots represent mean ± SEM. Each data point represents a mouse. All statistical analyses were performed using one-way ANOVA and Tukey's, Bonferroni's and Dunn's multiple comparison tests. All *post-hoc* tests gave the same results. $^{*}P < 0.05$, $^{**}P < 0.01$.

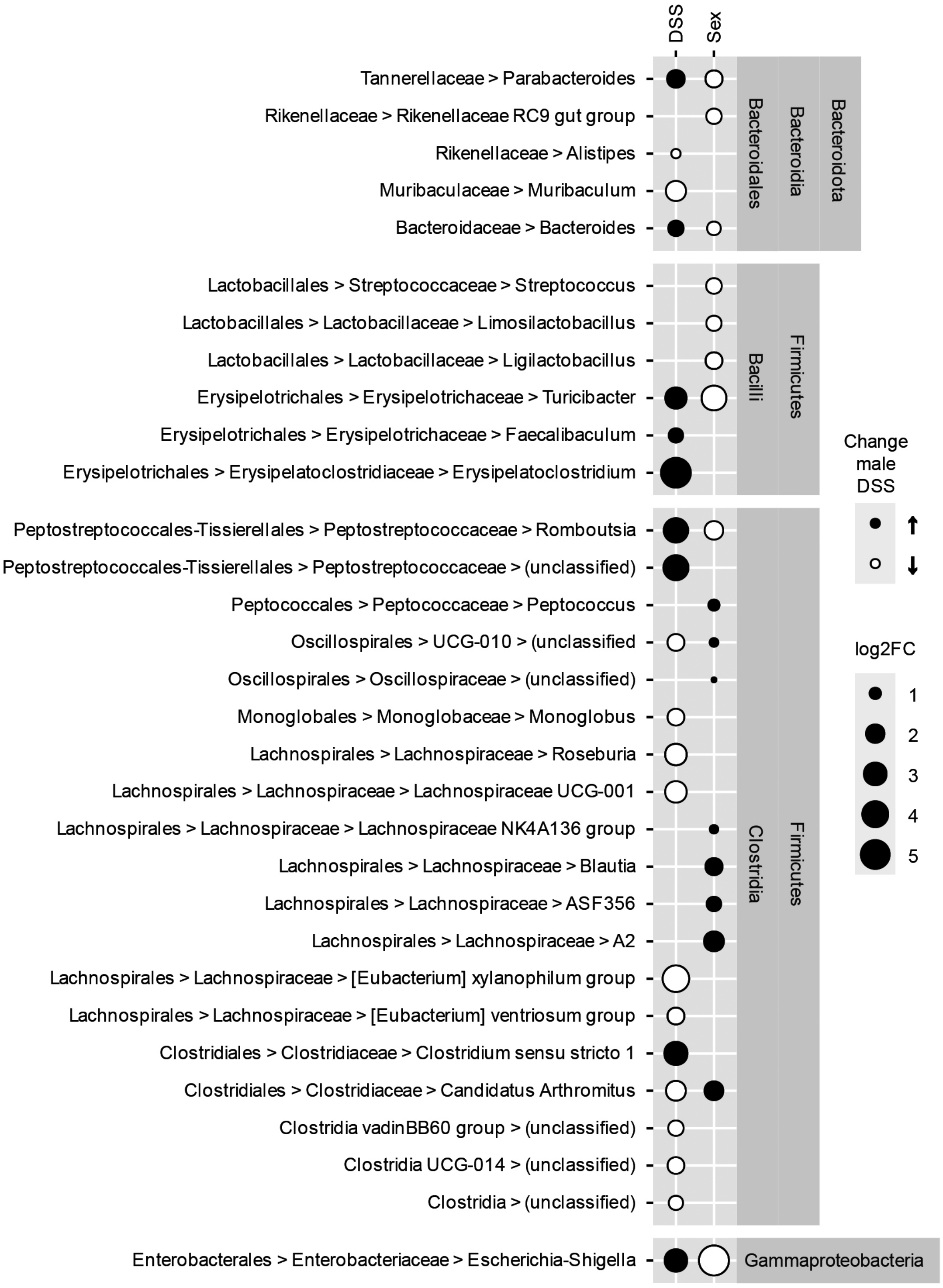

Figure EV4. Differences in the microbiome composition of female versus male and untreated versus DSS-treated mice.

A DESeq2-derived bubble plot with sex-specific and DSS-specific log-fold changes in microbiome composition on genus level is shown. The changes comprised several genera of the classes clostridia, bacilli and gammaproteobacteria as well as the order bacteroidales. "Unclassified" includes all taxa not classified at genus rank for given family or class.

 © 2023 The Authors

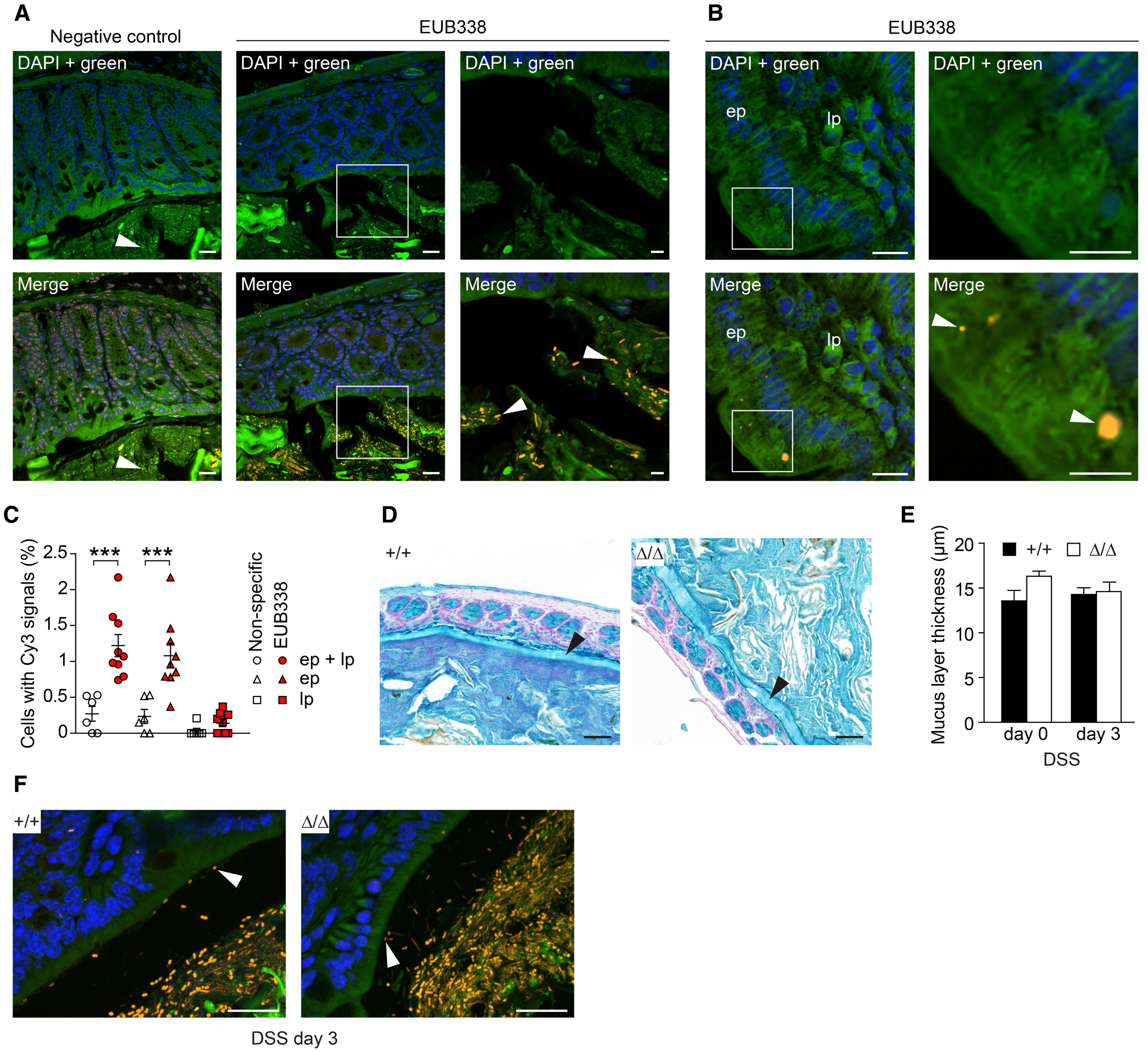
A
Negative control
EUB338
DAPI + green
Merge
B
EUB338
DAPI + green
ep
lp
Merge
C
Cells with Cy3 signals (%)
2.5
2
1.5
1
0.5
0
***
***
Non-specific
EUB338
ep + lp
ep
lp
D
+/+
Δ/Δ
E
Mucus layer thickness (µm)
20
15
10
5
0
+/+
Δ/Δ
day 0
day 3
DSS
F
+/+
Δ/Δ
DSS day 3

Figure EV5.

◀ **Figure EV5. Evaluation of bacterial FISH analysis and mucus layer permeability after DSS treatment in CDHR5$^{+/+}$ and CDHR5$^{\Delta/\Delta}$ mice.**

A Spinning disc fluorescence images of mouse colon for Cy3-labeled bacteria stained with the pan-bacterial FISH probe EUB338 (orange), nuclear DAPI (blue) and green autofluorescence (upper images without Cy3 and lower images merged). For the negative control, a Cy3-labeled non-specific probe was used. Non-specific signals were observed in cell nuclei and in feces (left images). Non-specific fecal signals were double-positive and showed also green autofluorescence (arrowhead). Accordingly, nuclear signals and double-positive signals were interpreted as false-positives and excluded from the analysis. Many fecal bacteria could be detected with the FISH probe (middle images with low magnification and right images with high magnification, the rectangle in the middle images marks the position of the right images). The bacterial signals (arrowheads) showed no green autofluorescence. Scale bar = 20 μm (low magnification) or 5 μm (high magnification).

B Example for epithelial Cy3-positive signals (arrowheads) that represent bacteria (low magnification left images and high magnification right images, the rectangle in the middle images marks the position of the right images). Upper images are without Cy3 and lower images are merged. Scale bar = 10 μm (low magnification) or 5 μm (high magnification).

C Scatter plots with quantitation of Cy3-positive signals (without green autofluorescence) in the mucosa on slides with negative control probe (non-specific background) and with FISH probe EUB338. About five times more signals were detected with EUB338 in the mucosa (epithelium plus lamina propria) and in the epithelium only than with the non-specific probe. All counts were performed on multiple images obtained with intestinal sections from ≥ 3 mice. Scatter plots represent mean ± SEM. Statistical analysis was performed using one-way ANOVA and Tukey's multiple comparison test. ***$P < 0.001$. ep, epithelium; lp, lamina propria.

D Alcian blue staining of the colonic mucus layer (arrowhead) of untreated CDHR5$^{+/+}$ and CDHR5$^{\Delta/\Delta}$ mice. Scale bar = 100 μm.

E Quantitation of the colonic mucus layer thickness in untreated (day 0) and DSS-treated (day 3) mice. Bars represent data ± SEM (3 CDHR5$^{+/+}$ and ≥ 3 CDHR5$^{\Delta/\Delta}$ mice, ≥ 11 thickness measurements and calculation of mean per mouse). Statistical analyses were performed using unpaired Student's *t*-test for day 0 and day 3 separately. Differences are not significant.

F Spinning disc fluorescence images showing invasion of bacteria into the colonic mucus layer (arrowheads) of CDHR5$^{+/+}$ and CDHR5$^{\Delta/\Delta}$ mice treated with DSS for 3 days. Colon sections were stained by FISH for bacteria with Cy3-labeled EUB338 (orange). Nuclei were stained with DAPI (blue) and the channel for green autofluorescence was included to delineate cell boundaries. Scale bar = 20 μm.

© 2023 The Authors
