# Supplementary material for: Defects in microvillus crosslinking sensitize to colitis and inflammatory bowel disease
Source: EMBO Rep. 2023 Sep 11;24(10):e57084. doi: 10.15252/embr.202357084 (PMC10561180; doi:10.15252/embr.202357084)
Supplement: Supplementary file 9 [file EMBR-24-e57084-s001.pdf]

EMBO reports

Defects in microvillus crosslinking sensitize to colitis and inflammatory bowel disease

Bernadette Mödl, Monira Awad, Daniela Zwolanek, Irene Scharf, Katharina Schwertner, Danijela Milovanovic, Doris Moser, Katy Schmidt, Petra Pjevac, Bela Hausmann, Dana Krauss, Thomas Mohr, Jasmin Svinka, Lukas Kenner, Emilio Casanova, Gerald Timelthaler, Maria Sibilia, Sigurd Krieger, and Robert Eferl
DOI: 10.15252/embr.202357084

Corresponding author(s): Robert Eferl (robert.eferl@meduniwien.ac.at)

Review Timeline:

Submission Date:	28th Feb 23
Editorial Decision:	17th Mar 23
Appeal Received:	7th Apr 23
Revision Received:	16th Jun 23
Editorial Decision:	20th Jul 23
Revision Received:	4th Aug 23
Accepted:	22nd Aug 23

Editor: Achim Breiling

Transaction Report:

(Note: With the exception of the correction of typographical or spelling errors that could be a source of ambiguity, letters and reports are not edited. Depending on transfer agreements, referee reports obtained elsewhere may or may not be included in this compilation. Referee reports are anonymous unless the Referee chooses to sign their reports.)

1st Editorial Decision **17th Mar 2023**

Dear Dr. Eferl

Thank you for the transfer of your research manuscript to EMBO reports. I have now received the full set of referee reports that is copied below.

I am sorry to say that the decision on your manuscript is not a positive one. As you will see, referees #21and #3 indicate that the conclusions are not convincingly supported by the data. Moreover, they mention technical shortcomings and insufficient data presentation and interpretation. Referee #2 is more positive, but also has concerns.

Given these comments, considering the amount of work required to address them, and the fact that EMBO reports can only invite revision of papers that receive strong support from the referees upon initial assessment, I cannot offer to publish your manuscript.

I am sorry to have to disappoint you this time. I nevertheless hope that the referee comments will be helpful in your continued work in this area, and I thank you once more for your interest in our journal.

Yours sincerely

Achim Breiling
Senior Editor
EMBO Reports

Referee #1:

Many structural aspects / protein components of the intestinal brush border have been elucidated, but how defects in brush border structure might mechanistically contribute to intestinal disease has not yet been investigated in detail. This is significant because brush border defects have recently been associated with worse clinical disease metrics in Crohn's disease. In this study, a novel mouse model of Cdhr5-deficiency was generated, characterized, and analyzed in three commonly used colitis models. From the Abstract and Introduction, the authors' intent was to use this mouse model to examine how brush border defects affect intestinal barrier function and colitis outcomes. Barrier function at homeostasis did not appear to be affected in these mice, and genotype-based differences in colitis outcomes are observed with only one (DSS) of the three colitis models. The authors speculate that DSS-induced disruptions of the mucus layer promote increased bacterial access to the epithelium and subsequent translocation in the Cdhr5-deficient vs. control mice, leading to the more severe colitis injury. However, it appears that goblet cells are also lost in the other 2 colitis models, which one would assume would also lead to diminished mucus layers. Mucus layers and bacterial translocation are not examined in these other 2 colitis models. Barrier function differences in the context of colitis (other than bacterial translocation) are not examined in the Cdhr5-deficient mice. There are also no experiments directly testing whether disruption of the mucus layer is the key mechanism promoting worse injury in the DSS model. As mice with brush border defects have been shown to be more sensitive to DSS in published reports (for example, Pls1-deficient mice), the main novel aspect of this manuscript would have been the mechanism of the more severe colitis injury. More mechanistic experiments are needed to support the conclusions and add to the novelty of this work.

Major comments:
1. The current title of this manuscript, "Brush border defects trigger colitis and inflammatory bowel disease", does not accurately describe what is tested or demonstrated by the experiments. If brush border defects "trigger" inflammation, then spontaneous inflammation should occur in the Cdhr5-deficient mice. This was not observed (data were not shown; this was simply stated in the Discussion section). The experiments were instead designed to test whether a brush border defect due to loss of Cdhr5 altered outcomes in 3 models of murine colitis, two of which did not show a genotype-based difference; one model (DSS) did suggest that the Cdhr5-deficient mice exhibited more histologic injury than control genotype mice. With regards to inflammatory bowel disease, the observation that CDHR5 mRNA expression is lower in the epithelial cells of UC patients does not lead to the conclusion that brush border defects trigger IBD; the Smillie et al. 2019 study dataset re-analyzed in this manuscript included UC patients with an established disease diagnosis and active disease, i.e., disease was already "triggered". These points should also be considered to revise statements and conclusions throughout the manuscript text.

2. The majority of the intestinal and brush border characterization data for the Cdhr5-deficient was performed in the duodenum, yet the intestinal inflammation / injury models used to challenge these mice target the colon. Also, the human data in Fig. 5 are from the colon. There is colonic baseline phenotype characterization data provided for the Cdhr5-deficient mice, but it is currently in a supplement. The authors should reconcile the organization of data vs. design of experiments.

3. This paper has generated a new mouse model to examine intestinal phenotypes. Yet, baseline quantification of the major epithelial cell type proportions, proliferation, or apoptosis was not performed for the small intestine and most of these metrics were also not examined in the colon.

4. The Fig. 2 data demonstrating IMAC disruption is not very convincing. The representative IF images do not appear to match their associated quantification (and the same goes for the enterocyte enzymes/transporter IF images). The representative images look fuzzy - what type of microscopy is this? The methods do not report whether confocal imaging was used for fluorescence quantification. Higher resolution microscopy may be needed for this analysis.

5. There is no validation data provided to support that epithelial cells were properly sorted, viable, and confirmed to have equal representation of enterocytes prior to RNA-seq analysis. Due to the linkage issue between the supplementary methods and cited works mentioned below, it is not clear how the epithelial purification was performed. This validation data is very important to include because previous publications of mouse models with brush border defects have reported increased fragility of epithelial cells. It is therefore possible that applying an epithelial cell purification technique could result in differing viability of the epithelial cell populations based on genotype.

6. Some quantifications (e.g. Fig. 1 J-L, Fig EV1) were performed with only 2 biological replicates (data from 2 mice). This is a major concern. The frequency distribution data and number of technical measurements are great. However, biological variation is typically greater than technical variation. Thus, analyses from a small number of mice could lower the rigor and reproducibility of this work and be more likely to produce false-positive statistical results.

7. The conclusions for genotype-based effects in the 3 colitis models are mostly based on histology index scores. A secondary, objective approach (for example, analysis of cytokine levels, immune cell infiltration, fecal lipocalin, etc.) should be used to support histologic scoring index results. It would also be helpful to provide low-power microscopy images of the DSS histology, as the current views are difficult to compare between genotypes.

8. Of the 3 colitis models examined, genotype-based differences in outcomes were only observed in 1 (DSS) of the 3 models. The authors suggest in their Discussion that this has to do with DSS reducing the mucus layer, thereby allowing bacteria to access the epithelium. However, if this is the mechanism of increased injury in DSS-treated Cdhr5-deficient mice vs. controls, then the Oxazolone and TNBS colitis models would need to not exhibit differences in goblet cell number, mucus layer thickness, or bacterial translocation. Based on the H&E images in Fig. 3, it does appear that goblet cells are lost in the Oxazolone and TNBS colitis models. In addition, the authors analysis of the mucus layer thickness (presumably from DSS-treated mice - the legend is unclear) showed no difference between the Cdhr5-deficient mice vs. controls. The barrier permeability assays should be repeated in the DSS model. The authors need to provide more data supporting their conclusions or need to consider whether their conclusions are aligned with their data.

9. The model presented in Fig. 6 contains speculative conclusions and should be reworked to focus on the observations made in this manuscript. For example, the effect of diet was not tested in this manuscript. The differences in bacterial translocation based on genotype are not conveyed in the model figure. It is not clear why Muc2 loss is included in the Cdhr5 +/+ portion of the model, as Muc2-deficiency was not examined in this manuscript.

10. In the Discussion, it is stated that "We have not observed spontaneous colitis in CDHR5-deficient mice [...] which is most likely due to the protective effect of the intestinal mucus layer". First, no data was shown to support the statement that the CDHR5-deficient mice do not develop spontaneous colitis. Second, it is speculation that the protective effect of the mucus layer is the likely reason that spontaneous colitis did not occur in this strain. No experiments directly tested whether removal of the mucus layer was sufficient for colitis induction in these mice.

Minor comments:
1. The Methods report that mice were age-matched, but the age of the mice is not reported for many of the experiments.

2. How many independent experiments were performed for the colitis models? These models typically yield variable degrees of inflammation and injury between experiments even within a single strain. How many mice were included in each colitis experiment? For the weight curves and histological scoring, were sex-based differences observed?

3. Because there is microbiome analysis in this study, it would be helpful if the authors reported the exact diet fed to the mice in the Methods.

4. References in the Appendix are cited with numbers, but the reference list is organized by first author last name. Thus, it is not possible to link these two sources to follow up on referenced works.

5. There is a typo in Fig. 5A -- should be "inflamed".

6. Regarding the statement "CDHR5-deficient mice did not develop adenomas up to the age of two years" in the Discussion: is this statement reporting "data not shown" by this group? There is no citation provided to support this statement.

Referee #2:

The intestinal brush border is an organized collection of specialized microvilli found on the surface of intestinal enterocyte epithelial cells. Brush border microvilli mediate nutrient absorption in the gut and also play an important role in host tissue defense. Genetic conditions that result in perturbations to the brush border can be life threatening, as seen with microvillus inclusion disease. More recently, it has become appreciated that key proteins involved in brush border assembly are markedly reduced in inflammatory bowel disease also. These include the components of an adhesion complex, known as the IMAC, previously discovered to mediate the proper formation of the brush border. The IMAC forms cadherin-based linkages that join neighboring microvilli together in order to control their organization and proper assembly into a cohesive brush border. These linkages are made of two protocadherins: CDHR2 and CDHR5. While loss of CDHR2 in mice is known to result in significant brush border defects, this manuscript by Modl et al attempts to investigate whether loss of CDHR5 results in intestinal defects.

This manuscript addresses an important question of the role of CDHR5 in intestinal biology, and will be of great interest to a relatively broad audience. The experiments are well-done and include the appropriate controls/ analysis. The manuscript, overall, was easy to read; the figures are well laid-out and easy to interpret with only minor issues. I have a few suggestions/questions for the authors.

Major Points:

1) It was recently shown by the Sotomayor group (Gray et al. PLoS Biology 2021) that CDHR2 and CDHR5 exhibit species-dependent properties. In humans, CDHR2 exhibits weak homophilic adhesion, while CDHR5 has no homophilic adhesion. In mice, it is reversed, with mouse CDHR5 exhibiting weak homophilic adhesion and mouse CDHR2 exhibiting no homophilic adhesion. This would suggest that loss of CDHR5 in mice would completely abolish the appearance of intermicrovillar adhesion links (no homophilic or heterophilic interactions). Can the authors confirm this happens? Do they see the loss of linkages in their CDHR5 KO mice?

2) It would also be interesting for the authors to test infection of the CDHR5 KO mice with Citrobacter as a model for enteropathogenic Escherichia coli (EPEC) and enterohaemorrhagic E. coli (EHEC) human infections. In et al Cell Mol Gastroenterol Hepatol. 2016 showed that EHEC secrete the serine protease, EspP, to initiates brush border damage through degradation of intermicrovillar adhesion links. Would the CDHR5 mice be more susceptible to infection?

Minor points:
1) Page 3 "In the IMAC, the extracellular domains of CDHR2 and CDHR5 mediate microvillus crosslinking while their intracellular domains are connected to the cytoplasmic proteins USH1C, MYO7B and ANKEV1B, which are anchored in the core actin bundle (Crawley et al., 2014a; Crawley et al., 2014b; Crawley et al, 2016)"
ANKEV1B is incorrect. The scaffold name is ANKS4B. Also note that another IMAC component was recently discovered, named calmodulin-like protein 4 (CALML4) (Choi et al. JBC 2020).

2) Fig 1F and Fig EV1E. It is unclear what "HPF" means in the Y-axis.
3) Fig 1I. You should point to an example of a fused microvillus for clarity.
4) Page 9. "To date, no pathological consequences of IMAC dysfunction have been reported". Not sure if this is true. Patients with a deletion of their USH1C gene were described very early on to have severe enteropathy (Bitner-Glindzicz et al Nat Gen 2000). This correlates with the severe ultrastructural defects seen in the gut of USH1C KO mice (Crawley et al., Cell 2014).

Referee #3:

The manuscript by Mödl and colleagues describes the phenotype of mice lacking DHR5, a member of the protocadherin family that is involved in the formation of an adhesive complex at the tips of microvilli (intermicrovillar adhesive complex, IMAC). The authors find that CDHR5 depletion results in an altered organization and morphology (including reduced length, increased thickness and lower density) of microvilli, as well as in an altered localization of microvillar tip-associated proteins. The authors also observe an increased susceptibility to DSS-induced colitis. They find that this increase is not due to a breakdown on the tight junctions or due to an altered microbiome but is associated with increased apoptotic cell death and increased crypt cell proliferation. Consistent with enhanced susceptibility to DSS-induced colitis, bacterial infiltration of the mucosa is increased in CDHR5 knockout mice. Finally, using published data sets from human patients with ulcerative colitis the authors observe a downregulation of several IMAC components in enterocytes of inflamed tissues. In summary, the study provides evidence that CDHR5 is required for the ordered assembly of the brush border in intestinal epithelial cells and that its absence enhances colitis after DSS-induced epithelial damage. The study is largely well performed and includes the proper controls.

As a weak point of the study, a more detailed analysis of the localization of microvillar components is missing. The study shows only stainings of ezrin and Ush1C (Fig. 2A), which, however, are of low quality with low resolution. Ezrin seems to be localized beneath Ush1C (Fig. 2A). Why? Shouldn't ezrin be localized along the entire length of microvilli? And shouldn't P-Ezrin be

localized at the tips to regulate the dynamic turnover of microvilli? Other microvillar tip markers such as CDHR2 and Myo7B are only displayed as line scans. Without immunofluorescence pictures at higher resolution and magnification it is not possible to judge the quality of the stainings and specificity of the antibodies. The same applies for DPPIV stainings (Fig. 2E) and in additon for the IF analyses of IAP, NHE3, P-Ezrin (Fig. EV2). The authors do not provide any information on the selection of these molecules. Also, where is CDHR5 localized? Is it expressed (and localized in microvilli) only by cells lining the intestinal villi or is it also expressed (and localized in microvilli) by cells present in crypts where it could regulate proliferation? The authors provide some information from published databases which suggest a predominant expression in enterocytes and goblet cells but no expression in stem cells or TA cells. The authors should provide IF stainings for CDHR5 and the other microvillar components at high resolution to provide a better insight into the laterations observed after loss of CDHR5. As a further notion, in contrast to the increased bacterial invasion observed after depletion of CDHR5, the cell biological aspects of CDHR5 depletion are poorly discussed.

Specific points:
1. The visualization of CDHR5 in WT, heterozygous and KO mice is very poor (Fig. S1D). The red fluorescence signals supposedly reflecting CDHR5 is still prominently present in Δ/Δ mice, albeit not at the apical membrane in the region depicted in the inset. The antibody does not seem to be specific. Also, the authors should provide images at higher magnification and resolution.
2. The authos claim that colonic microvilli are disorganized and shortened and refer to Appendix Fig. S3B. This figure shows intestinal villi and does not provide information on microvilli organization.
3. The notion "normalization for brush border thickness abolished differences of apical marker expression in CDHR5+/+ and CDHR5-/- mice" (Results, paragraph 2) is unclear. Which difference is abolished? There is still apical localization of Ush1C (albeit not exclusive anymore). Similarly, the authors claim "the total amount of apical markers is reduced". Total amount means "total protein amount". Do the authors mean localization? How can they exclude that the "reduced amount of apical markers" is due to (or contributed to by) reduced protein expression? Did they perform Western blot experiments?
4. All EM micrographs lack scale bars.
5. The Volcano plots shown in Fig. 2I lack sufficient labeling and/or explanation. What is the color code? The legend provides no information. The readers can only speculate.
6. The labeling to Fig. 2B-C is unclear. What is represented by the dotted line? Does it indicate the microvillar tip? What is the labeling of the X-axis, "tip distance (µm)" or "distance (µm)"? As pointed out above, the authors should provide representative IF images for each staining.
7. For Fig. 2E, images of DPPIV with higher magnification are required to judge the intensity and localization of DPPIV.
8. What is shown in Fig. EV2B, what is represented by the green fluorescence?
9. The terminology "thickness" and "intensity/thickness" for fluorescence signals is very uncommon. What do the authors means when saying DPPIV thickness, IAP thickness, NHE3 thickness,....? This is not scientific standard.
10. The notion "CDHR5 has mainly structural functions....but no major function in intracellular signaling" is a misconception. Intracellular signaling is not defined by changes in the levels of mRNAs ("gene expression").
11. The Methods section is incomplete. For example, a section describing antibodies used for IF stainings is missing. Also, how were fluorescence signal intensities measured? How were line scans performed? A section describing RNA isolation and sequencing is also missing.
12. In some figures it is not clear if the significance values (*) apply to all data sets or only to the two "outer" data sets (e.g. Fig. 3C, K). In other figures, showing statistical significance values for all data points provides not useful information (e.g. Fig. 4C, E). As one example, it is not helpful to compare WT mice at day 0 with KO mice at day 3 or 7 (Fig. 4C, E, G). The authors should also indicate non-significant differences in the figures where applicable. Also, the violin plots shown in Fig. 5D display significance values in a selective manner. Why? For example, for CDHR2 the difference between healthy and non-inflamed tissue is indicated with three stars. What about the differences between healthy and inflamed or between inflamed and non-inflamed?
13. In Fig. 3F bacteria (arrowheads) are not really visible for the reader at the given magnification (scale bar = 100 µm!).
14. In Fig. 4G, it is not clear what the Y-axis depicts. The Y-axis legend reads "signals / cells (%)". Signals per how many cells?
15. The methods used for quantification are mentioned in the Methods section. However, it is not clear which method has been used in a given experiment. This information must be included in the figure legends.
16. The notion "...the phenotypes observed in CDHR2-deficient mice...were phenocopied in CDHR5-deficient mice indicating that the protocadherins have overlapping function" (2nd paragraph in discussion) provides a poor discussion/interpretation of the observations. In fact, the two protocadherins have non-overlapping functions as they form a functional adhesive complex. Given that mouse CDHR5 can undergo homophilic interactions whereas mouse CDHR2 cannot (Gray et al (2022) PLoS Biol), it is clear that CDHR5 is a critical component of the IMAC to serve as binding partner for CDHR2 to mediate trans-interactions of microvilli.

Minor issues
1. In the Introduction, ezrin is mentioned to be a member of the ERM family as well as a member of the myosin family. This needs to be corrected.

** As a service to authors, EMBO Press provides authors with the ability to transfer a manuscript that one journal cannot offer to

publish to another journal, without the author having to upload the manuscript data again. To transfer your manuscript to another EMBO Press journal using this service, please click on
Link Not Available

Dear Dr. Breiling!

Many thanks for your response. We highly appreciate the reviewer´s comments and your decision. However, after carefully studying the comments we are very surprised about the bad news. Only referee #1 declared major concerns, which are already addressed in the attached letter to reviewers. Referee #2 is very positive and we could not find any concern in his/her response. Referee #3 is also quite positive and only raises minor points. The majority of comments comprise the addition of more data or restructuring the manuscript, only referee #2 suggested a time-consuming experiment: ("It would also be interesting for the authors to test infection of the CDHR5 KO mice with Citrobacter as a model for enteropathogenic Escherichia coli (EPEC) and enterohaemorrhagic E. coli (EHEC) human infections"). We totally agree with the referee but think that infection experiments should be performed and published in a separate manuscript. Since we have already performed most of the other experiments the referees suggest the amount of work to deal with the additional items could be completed in two weeks. Therefore, we could provide a revised version of our manuscript very soon. We kindly ask you to read our letter to reviewers carefully and would highly appreciate if you reconsider your decision. Thank you!

With warm regards,

Robert

Dear Robert,

Thank you for your letter and the preliminary p-b-p-response. I now looked through these, and would be willing to consider a revised manuscript. Please submit the revised paper as new submission including your final detailed point-by-point response (addressing all points made by the referees) and indicate during submission and in your cover letter that this is a re-submission, also mentioning the previous manuscript number and this conversation. Our assistant will then link this to the previous version.

Please also review the instructions that follow below to speed up the process.

PLEASE NOTE THAT upon resubmission revised manuscripts are subjected to an initial quality control prior to exposition to re-review. Upon failure in the initial quality control, the manuscripts are sent back to the authors, which may lead to delays. Frequent reasons for such a failure are the lack of the data availability section (please see below) and the presence of statistics based on n=2 (the authors are then asked to present scatter plots or provide more data points).

When submitting your revised manuscript, we will require:

1) a .docx formatted version of the final manuscript text (including legends for main figures, EV figures and tables), but without the figures included. Figure legends should be compiled at the end of the manuscript text.

2) individual production quality figure files as .eps, .tif, .jpg (one file per figure), of main figures (up to 8) and EV figures. Please upload these as separate, individual files upon re-submission.

The Expanded View format, which will be displayed in the main HTML of the paper in a collapsible format, has replaced the Supplementary information. You can submit up to 5 images as Expanded View. Please follow the nomenclature Figure EV1, Figure EV2 etc. The figure legend for these should be included in the main manuscript document file in a section called Expanded View Figure Legends after the main Figure Legends section. Additional Supplementary material should be supplied as a single pdf file labeled Appendix. The Appendix should have page numbers and needs to include a table of content on the first page (with page numbers) and legends for all content. Please follow the nomenclature Appendix Figure Sx, Appendix Table Sx etc. throughout the text, and also label the figures and tables according to this nomenclature.

For more details, please refer to our guide to authors:
http://www.embopress.org/page/journal/14693178/authorguide#manuscriptpreparation

Please consult our guide for figure preparation:
http://wol-prod-cdn.literatumonline.com/pb-assets/embo-site/EMBOPress_Figure_Guidelines_061115-1561436025777.pdf

See also the guidelines for figure legend preparation:
https://www.embopress.org/page/journal/14693178/authorguide#figureformat

3) a complete author checklist, which you can download from our author guidelines (https://www.embopress.org/page/journal/14693178/authorguide). Please insert page numbers in the checklist to indicate where the requested information can be found in the manuscript. The completed author checklist will also be part of the RPF.

Please also follow our guidelines for the use of living organisms, and the respective reporting guidelines: http://www.embopress.org/page/journal/14693178/authorguide#livingorganisms

4) that primary datasets produced in this study (e.g. RNA-seq, ChIP-seq, structural and array data) are deposited in an appropriate public database. If no primary datasets have been deposited, please also state this in a dedicated section (e.g. 'No primary datasets have been generated and deposited'), see below.

See also: http://embor.embopress.org/authorguide#datadeposition

Please remember to provide a reviewer password if the datasets are not yet public.

The accession numbers and database should be listed in a formal "Data Availability" section (placed after Materials & Methods) that follows the model below. This is now mandatory (like the COI statement). Please note that the Data Availability Section is restricted to new primary data that are part of this study. This section is mandatory. As indicated above, if no primary datasets have been deposited, please state this in this section

Data availability

The datasets produced in this study are available in the following databases:

- RNA-Seq data: Gene Expression Omnibus GSE46843 (https://www.ncbi.nlm.nih.gov/geo/query/acc.cgi?acc=GSE46843)
- [data type]: [name of the resource] [accession number/identifier/doi] ([URL or identifiers.org/DATABASE:ACCESSION])

*** Note - All links should resolve to a page where the data can be accessed. ***

5) Our journal encourages inclusion of *data citations in the reference list* to directly cite datasets that were re-used and obtained from public databases. Data citations in the article text are distinct from normal bibliographical citations and should directly link to the database records from which the data can be accessed. In the main text, data citations are formatted as follows: "Data ref: Smith et al, 2001" or "Data ref: NCBI Sequence Read Archive PRJNA342805, 2017". In the Reference list, data citations must be labeled with "[DATASET]". A data reference must provide the database name, accession number/identifiers and a resolvable link to the landing page from which the data can be accessed at the end of the reference. Further instructions are available at: http://www.embopress.org/page/journal/14693178/authorguide#referencesformat

6) Regarding data quantification and statistics, please make sure that the number "n" for how many independent experiments were performed, their nature (biological versus technical replicates), the bars and error bars (e.g. SEM, SD) and the test used to calculate p-values is indicated in the respective figure legends (also for potential EV figures and all those in the final Appendix). Please also check that all the p-values are explained in the legend, and that these fit to those shown in the figure. Please provide statistical testing where applicable. Please avoid the phrase 'independent experiment', but clearly state if these were biological or technical replicates. Please also indicate (e.g. with n.s.) if testing was performed, but the differences are not significant. In case n=2, please show the data as separate datapoints without error bars and statistics. See also:
http://www.embopress.org/page/journal/14693178/authorguide#statisticalanalysis

If n<5, please show single datapoints for diagrams.

7) Please add scale bars of similar style and thickness to microscopic images, using clearly visible black or white bars (depending on the background). Please place these in the lower right corner of the images themselves. Please do not write on or near the bars in the image but define the size in the respective figure legend.

8) Please also note our reference format:
http://www.embopress.org/page/journal/14693178/authorguide#referencesformat

9) We updated our journal's competing interests policy in January 2022 and request authors to consider both actual and perceived competing interests. Please review the policy https://www.embopress.org/competing-interests and update your competing interests if necessary. Please name this section 'Disclosure and Competing Interests Statement' and put it after the Acknowledgements section.

10) We now use CRediT to specify the contributions of each author in the journal submission system. CRediT replaces the author contribution section. Please use the free text box to provide more detailed descriptions and do not show an author contribution section in the manuscript text file. See also guide to authors:
https://www.embopress.org/page/journal/14693178/authorguide#authorshipguidelines

11) Please order the manuscript sections like this, using these names:
Title page - Abstract – Keywords - Introduction - Results - Discussion - Materials and Methods - Data availability section - Acknowledgements - Disclosure and Competing Interests Statement - References - Figure legends - Expanded View Figure legends

Finally, please note that all corresponding authors are required to supply an ORCID ID for their name upon submission of a revised manuscript. Please find instructions on how to link the ORCID ID to the account in our manuscript tracking system in our Author guidelines:
http://www.embopress.org/page/journal/14693178/authorguide#authorshipguidelines

All the best,

Achim

Achim Breiling, PhD
Senior Editor | EMBO reports
a.breiling@emboreports.org

Response to reviewers

Referee #1:

1) Many structural aspects / protein components of the intestinal brush border have been elucidated, but how defects in brush border structure might mechanistically contribute to intestinal disease has not yet been investigated in detail. This is significant because brush border defects have recently been associated with worse clinical disease metrics in Crohn's disease. In this study, a novel mouse model of Cdhr5-deficiency was generated, characterized, and analyzed in three commonly used colitis models. From the Abstract and Introduction, the authors' intent was to use this mouse model to examine how brush border defects affect intestinal barrier function and colitis outcomes. Barrier function at homeostasis did not appear to be affected in these mice, and genotype-based differences in colitis outcomes are observed with only one (DSS) of the three colitis models. The authors speculate that DSS-induced disruptions of the mucus layer promote increased bacterial access to the epithelium and subsequent translocation in the Cdhr5-deficient vs. control mice, leading to the more severe colitis injury. However, it appears that goblet cells are also lost in the other 2 colitis models, which one would assume would also lead to diminished mucus layers. Mucus layers and bacterial translocation are not examined in these other 2 colitis models. Barrier function differences in the context of colitis (other than bacterial translocation) are not examined in the Cdhr5-deficient mice.

The T cell-based models of colitis (oxazolone and TNBS) result in rapid (within 1-2 days) immunological destruction of intestinal epithelial cells. Protective effects of the mucus layer or brush border may not be effective in these colitis models because epithelial cells are immunologically destroyed and bacteria can enter via the unrestricted route (i.e., no barrier because the epithelium is lost). Rather, the lack of difference in these models reinforces the importance of brush border cross-linking in the DSS model, which is based on luminal effects on epithelial cells. We have now made this point clear in the discussion of the revised manuscript. In our experience, gavage experiments with FITC-dextran or other test substances for the barrier function yield heterogeneous results in colitogenic mice, since they could be taken up via the unrestricted route. Bacteria can be directly visualized in situ with FISH, but FITC-dextran or other test substances must be measured in the blood.

2) There are also no experiments directly testing whether disruption of the mucus layer is the key mechanism promoting worse injury in the DSS model.

We agree with the reviewer and changed the wording accordingly in the abstract of our revised manuscript. To prove our statement about the protective effect of the mucus layer would require an experimental approach that leads to a slight increase in the permeability of the mucus layer. Genetic deletion of mucus components such as Muc2 leads to severe mucus layer defects with associated colitis. Under such harsh conditions, the protective barrier effect of the brush border would be bypassed and not visible. We tried using high-fat diet feeding alongside DSS to induce a small increase in barrier permeability, but this was not sufficient to induce colitis in CDHR5$^{+/+}$ or CDHR5$^{\Delta/\Delta}$ mice (data not shown). We showed that DSS treatment favored bacterial access to the luminal epithelial membrane (Fig EV5F of our original and revised manuscript), which correlated with increased bacterial penetration and colitis in CDHR5$^{\Delta/\Delta}$ mice. We argue that this is a strong correlation.

3) As mice with brush border defects have been shown to be more sensitive to DSS in published reports (for example, Pls1-deficient mice), the main novel aspect of this manuscript would have been

the mechanism of the more severe colitis injury. More mechanistic experiments are needed to support the conclusions and add to the novelty of this work.

Pls1-deficient mice have brush border defects, but Pls1 is not part of the IMAC. We wanted to point out that the IMAC and in particular microvillus crosslinking by protocadherins protects against bacterial invasion. We apologize for the misunderstanding and replaced the term "brush border defects" with “microvillus crosslinking defects” in our revised manuscript whenever it was related to a pathological interpretation. We also included a discussion on Pls1-deficient mice.

Major comments:

4. The current title of this manuscript, "Brush border defects trigger colitis and inflammatory bowel disease", does not accurately describe what is tested or demonstrated by the experiments. If brush border defects "trigger" inflammation, then spontaneous inflammation should occur in the Cdhr5-deficient mice. This was not observed (data were not shown; this was simply stated in the Discussion section). The experiments were instead designed to test whether a brush border defect due to loss of Cdhr5 altered outcomes in 3 models of murine colitis, two of which did not show a genotype-based difference; one model (DSS) did suggest that the Cdhr5-deficient mice exhibited more histologic injury than control genotype mice. With regards to inflammatory bowel disease, the observation that CDHR5 mRNA expression is lower in the epithelial cells of UC patients does not lead to the conclusion that brush border defects trigger IBD; the Smillie et al. 2019 study dataset re-analyzed in this manuscript included UC patients with an established disease diagnosis and active disease, i.e., disease was already "triggered". These points should also be considered to revise statements and conclusions throughout the manuscript text.

We have shown in Fig 4A (day 0 of DSS treatment) of the original manuscript that there is no spontaneous colitis in CDHR5$^{\Delta/\Delta}$ mice. Hence, we have included additional images of H&E-stained colon sections in the new Fig EV2 of the revised manuscript confirming this result. We agree with the reviewer and have changed the title of our manuscript accordingly. We also changed our interpretation of expression data in UC patients accordingly, replacing “triggered” with “sensitized” throughout the manuscript.

5. The majority of the intestinal and brush border characterization data for the Cdhr5-deficient was performed in the duodenum, yet the intestinal inflammation / injury models used to challenge these mice target the colon. Also, the human data in Fig. 5 are from the colon. There is colonic baseline phenotype characterization data provided for the Cdhr5-deficient mice, but it is currently in a supplement. The authors should reconcile the organization of data vs. design of experiments.

We moved the brush border characterization of the colon (previous Fig EV1) to Fig 1 of the revised manuscript.

6. This paper has generated a new mouse model to examine intestinal phenotypes. Yet, baseline quantification of the major epithelial cell type proportions, proliferation, or apoptosis was not performed for the small intestine and most of these metrics were also not examined in the colon.

We have included the requested baseline quantification in the revised manuscript as new Appendix Fig S2.

7. The Fig. 2 data demonstrating IMAC disruption is not very convincing. The representative IF images do not appear to match their associated quantification (and the same goes for the enterocyte enzymes/transporter IF images). The representative images look fuzzy - what type of microscopy is

this? The methods do not report whether confocal imaging was used for fluorescence quantification. Higher resolution microscopy may be needed for this analysis.

We fully agree with the reviewer and performed new IF staining and analysis for IMAC proteins with a spinning disc microscope (Olympus IXplore SpinSR). We included corresponding high magnification images for USH1C/Ezrin, CDHR2/Ezrin and MYO7B/Ezrin in the revised Fig 2A. Lower magnification images are now shown as Appendix Fig S4. We also performed new profile plots that are now centered on the maximum Ezrin signal (Fig 2B) and provide a Spearman correlation coefficient for colocalization of IMAC components with Ezrin (Fig 2C). Furthermore, we included a correlation matrix for colocalization (Fig 2D). All these data show that IMAC components are not properly localized at the microvillus tips but rather localize like Ezrin along the whole microvillus. Regarding DPPIV, IAP, NHE3, P-Ezrin and Ezrin, we included higher magnification laser scanning IF images (Fig 2E) and detailed the method for quantitation of IF images in the Methods section of the Appendix and briefly in the legend of Fig 2F. We measured the amount of DPPIV, IAP, NHE3, P-Ezrin and Ezrin proteins with ImageJ using profile intensity of IF images. The area under the curve was calculated and used as measure for protein amount. We changed "relative intensity" for "AUC" in the corresponding scatter plots accordingly.

8. There is no validation data provided to support that epithelial cells were properly sorted, viable, and confirmed to have equal representation of enterocytes prior to RNA-seq analysis. Due to the linkage issue between the supplementary methods and cited works mentioned below, it is not clear how the epithelial purification was performed. This validation data is very important to include because previous publications of mouse models with brush border defects have reported increased fragility of epithelial cells. It is therefore possible that applying an epithelial cell purification technique could result in differing viability of the epithelial cell populations based on genotype.

We agree with the reviewer that the quality and purity of the epithelial cell preparations are crucial. We used a protocol for gentle isolation of intestinal epithelial cells that preserves cell viability. The protocol is based on inverting the intestine and scraping off whole epithelial layers. We also use this protocol routinely for organoid cultures. The method is now detailed the Methods section of the Appendix in the revised manuscript. The RNA integrity numbers (RIN values) of RNAs isolated from epithelial preparations were very high, indicating preserved cell viability. We controlled the purity of isolated epithelial cells using qPCR for epithelial (CDH1, CDHR5) and lamina propria (FN1, PDGFRA, ACTA2) markers before RNA-seq analysis. The qPCR demonstrated that the preparations are very pure with no significant contamination of lamina propria cells. We also extracted expression of the markers from the RNA-seq data confirming high purity of the epithelial cells. Examples for RNA quality and expression analyses for cell purity are now included as Appendix Fig S5A-C in the revised manuscript. Please note the logarithmic scale in Appendix Fig S5B, C. Also note that the expression values in Appendix Fig S5B were normalized for expression values in corresponding whole gut preparations (i.e., in whole gut RNA, all expression values would be 1).

9. Some quantifications (e.g. Fig. 1 J-L, Fig EV1) were performed with only 2 biological replicates (data from 2 mice). This is a major concern. The frequency distribution data and number of technical measurements are great. However, biological variation is typically greater than technical variation. Thus, analyses from a small number of mice could lower the rigor and reproducibility of this work and be more likely to produce false-positive statistical results.

We performed additional quantifications on three biological replicates which did not alter the main result of original Fig 1J-L, Fig EV1 except for the microvillus area in the colon. Similar to the small intestine, the microvillus area is now also significantly larger in the colon of CDHR5$^{\Delta/\Delta}$ mice. The difference seems small, but significance is reached by including the additional measurements (new Fig 1T). We have exchanged the graphs in original Fig 1G, H, Fig 1J-L, Fig EV1 H-J in the revised version. All data of the new graphs are now derived from 3 biological replicates per genotype and are shown in new Fig 1.

10. The conclusions for genotype-based effects in the 3 colitis models are mostly based on histology index scores. A secondary, objective approach (for example, analysis of cytokine levels, immune cell infiltration, fecal lipocalin, etc.) should be used to support histologic scoring index results. It would also be helpful to provide low-power microscopy images of the DSS histology, as the current views are difficult to compare between genotypes.

In our experience, the analysis of cytokine levels in the DSS model is problematic because they are highly variable and rarely provide a significant result. In the paper on the DSS-treated Pls1-deficient mice mentioned by the reviewer (see above), merely a survival curve and histological scoring were shown to assess the DSS effects. We included weight analysis and measurement of colon length as objective approaches to assess the severity of colitis (Fig 3C, H). In addition, the infiltration of immune cells is part of the scoring system and was shown in Fig EV3A of the original manuscript (Fig EV3B in the revised manuscript). Since all results fit with the total colitis score (Fig 3K), we argue that aggravated DSS-induced colitis is sufficiently evident in CDHR5 mice. As suggested by the reviewer, we included low-power microscopy images of colitogenic mice in the revised manuscript (new Fig EV3A).

11. Of the 3 colitis models examined, genotype-based differences in outcomes were only observed in 1 (DSS) of the 3 models. The authors suggest in their Discussion that this has to do with DSS reducing the mucus layer, thereby allowing bacteria to access the epithelium. However, if this is the mechanism of increased injury in DSS-treated Cdhr5-deficient mice vs. controls, then the Oxazolone and TNBS colitis models would need to not exhibit differences in goblet cell number, mucus layer thickness, or bacterial translocation. Based on the H&E images in Fig. 3, it does appear that goblet cells are lost in the Oxazolone and TNBS colitis models.

Please see our response to point 1.

12. In addition, the authors analysis of the mucus layer thickness (presumably from DSS-treated mice - the legend is unclear) showed no difference between the Cdhr5-deficient mice vs. controls. The barrier permeability assays should be repeated in the DSS model. The authors need to provide more data supporting their conclusions or need to consider whether their conclusions are aligned with their data.

We have shown mucus layer thickness only in untreated mice (Fig EV5D, E of the original manuscript) where no difference between the genotypes was detected. We have now also analyzed the mucus layer thickness of DSS-treated mice, which is similar to that of untreated mice and does not differ between genotypes (Fig EV5E of the revised manuscript). However, thickness is not an indication of permeability. Fig 4F of the original and the revised manuscript shows that bacteria did not reach the surface of epithelial cells in untreated mice of either genotype, suggesting that the mucus layer keeps them apart. In contrast, bacteria reached the epithelial surface in both genotypes after DSS treatment (Fig EV5F of the original and the revised

manuscript), showing that the mucus layer became permeable. This agrees with published data (reference Johansson et al, 2014 in the original manuscript). In the cited reference, the authors showed increased permeability of the mucus layer as early as 12 hours after DSS treatment, although the mucus layer appeared normal. Similar to our data, the authors analyzed the permeability of the mucus layer through FISH and found bacteria in close contact with the non-inflamed epithelium. Therefore, we believe that the DSS effects on mucus layer permeability are strongly supported by literature data.

13. The model presented in Fig. 6 contains speculative conclusions and should be reworked to focus on the observations made in this manuscript. For example, the effect of diet was not tested in this manuscript. The differences in bacterial translocation based on genotype are not conveyed in the model figure. It is not clear why Muc2 loss is included in the Cdhr5 +/+ portion of the model, as Muc2-deficiency was not examined in this manuscript.

We agree with the reviewer and have changed the model and the Figure legend accordingly (revised Fig 6).

14. In the Discussion, it is stated that "We have not observed spontaneous colitis in CDHR5-deficient mice [...] which is most likely due to the protective effect of the intestinal mucus layer". First, no data was shown to support the statement that the CDHR5-deficient mice do not develop spontaneous colitis. Second, it is speculation that the protective effect of the mucus layer is the likely reason that spontaneous colitis did not occur in this strain. No experiments directly tested whether removal of the mucus layer was sufficient for colitis induction in these mice.

Concerning the development of spontaneous colitis, please see our answer to point 4. For experiments directly testing whether removal of the mucus layer is sufficient to induce colitis in CDHR5$^{\Delta/\Delta}$, please see our answer to point 2. We agree with the reviewer that we did not test the mucus layer hypothesis directly and reformulated the statement accordingly in a revised manuscript.

Minor comments:

15. The Methods report that mice were age-matched, but the age of the mice is not reported for many of the experiments.

We used 6–9 weeks old mice for all experiments. This information is now provided in the methods section.

16. How many independent experiments were performed for the colitis models? These models typically yield variable degrees of inflammation and injury between experiments even within a single strain. How many mice were included in each colitis experiment? For the weight curves and histological scoring, were sex-based differences observed?

Scoring was performed with male mice and the animal number is indicated by the number of circles, triangles or squares in the scatter plots of Fig 3 in the revised manuscript. We have clarified in the methods section of the revised manuscript that male mice were used for colitis scoring. Separate cohorts including female mice (n = 3 per experimental group) were treated with DSS for timepoints shown in Fig 4 and for the microbiome experiments (Fig EV4 and Appendix Fig S7, 8). We could not assess sex-specific differences in these smaller cohorts, but we observed greater weight loss of CDHR5$^{\Delta/\Delta}$ mice compared to CDHR5$^{+/+}$ mice in general in these experiments. Furthermore, as shown in Fig 4C-G, both sexes of CDHR5$^{\Delta/\Delta}$ mice showed increased bacterial

infiltration (day 3 of DSS treatment), increased apoptosis (day 7 of DSS treatment) and increased proliferation (day 7 of DSS treatment). We therefore believe that increased susceptibility of CDHR5$^{\Delta/\Delta}$ mice to DSS-induced colitis is not sex-dependent.

17. Because there is microbiome analysis in this study, it would be helpful if the authors reported the exact diet fed to the mice in the Methods.
Mice were fed a standard diet, which is now detailed in the paragraph "Mice" in the Methods section of the revised manuscript.

18. References in the Appendix are cited with numbers, but the reference list is organized by first author last name. Thus, it is not possible to link these two sources to follow up on referenced works.
We have changed that accordingly.

19. There is a typo in Fig. 5A -- should be "inflamed".
We have changed that accordingly.

20. Regarding the statement "CDHR5-deficient mice did not develop adenomas up to the age of two years" in the Discussion: is this statement reporting "data not shown" by this group? There is no citation provided to support this statement.
This is our own observation. The journal does not allow a "data not shown" statement and we did not intend to show gut preparations from two-year-old mice with no pathology. Therefore, we have removed this statement in the discussion of the revised manuscript.

Referee #2:
The intestinal brush border is an organized collection of specialized microvilli found on the surface of intestinal enterocyte epithelial cells. Brush border microvilli mediate nutrient absorption in the gut and also play an important role in host tissue defense. Genetic conditions that result in perturbations to the brush border can be life threatening, as seen with microvillus inclusion disease. More recently, it has become appreciated that key proteins involved in brush border assembly are markedly reduced in inflammatory bowel disease also. These include the components of an adhesion complex, known as the IMAC, previously discovered to mediate the proper formation of the brush border. The IMAC forms cadherin-based linkages that join neighboring microvilli together in order to control their organization and proper assembly into a cohesive brush border. These linkages are made of two protocadherins: CDHR2 and CDHR5. While loss of CDHR2 in mice is known to result in significant brush border defects, this manuscript by Modl et al attempts to investigate whether loss of CDHR5 results in intestinal defects.
This manuscript addresses an important question of the role of CDHR5 in intestinal biology, and will be of great interest to a relatively broad audience. The experiments are well-done and include the appropriate controls/ analysis. The manuscript, overall, was easy to read; the figures are well laid-out and easy to interpret with only minor issues. I have a few suggestions/questions for the authors.

Major Points:
1) It was recently shown by the Sotomayor group (Gray et al. PLoS Biology 2021) that CDHR2 and CDHR5 exhibit species-dependent properties. In humans, CDHR2 exhibits weak homophilic adhesion, while CDHR5 has no homophilic adhesion. In mice, it is reversed, with mouse CDHR5 exhibiting weak homophilic adhesion and mouse CDHR2 exhibiting no homophilic adhesion. This would suggest that loss of CDHR5 in mice would completely abolish the appearance of intermicrovillar adhesion links (no homophilic or heterophilic interactions). Can the authors confirm this happens? Do they see the loss of linkages in their CDHR5 KO mice?
The Sotomayor group has demonstrated these interactions primarily with structural and bead aggregation assays. A direct demonstration of the interactions in vivo using electron microscopy or alternative high-resolution techniques could be challenging. The data of the Sotomayor group suggest that a residual microvillus crosslinking function is still present in CDHR2 KO mice due to homophilic CDHR5 interactions. In contrast, CDHR5 KO mice should lack any protocadherin-mediated microvillus crosslinking since there are no CDHR2 homophilic interactions. However, the similarity of the brush border phenotypes in CDHR2 KO (reference Pinette et al., 2019 in our manuscript) and CDHR5 KO (data of our manuscript) suggests that the possibly weak homophilic interactions between CDHR5 are not sufficient to improve brush border organization in CDHR2 KO mice significantly. We have rephrased the relevant paragraph and included this important point in the discussion of the revised manuscript.

2) It would also be interesting for the authors to test infection of the CDHR5 KO mice with Citrobacter as a model for enteropathogenic Escherichia coli (EPEC) and enterohaemorrhagic E. coli (EHEC) human infections. In et al Cell Mol Gastroenterol Hepatol. 2016 showed that EHEC secrete the serine protease, EspP, to initiates brush border damage through degradation of intermicrovillar adhesion links. Would the CDHR5 mice be more susceptible to infection?
We fully agree with the reviewer but think that infection experiments would be better suited in a separate manuscript.

Minor points:

1) Page 3 "In the IMAC, the extracellular domains of CDHR2 and CDHR5 mediate microvillus crosslinking while their intracellular domains are connected to the cytoplasmic proteins USH1C, MYO7B and ANKEV1B, which are anchored in the core actin bundle (Crawley et al., 2014a; Crawley et al., 2014b; Crawley et al, 2016)" ANKEV1B is incorrect. The scaffold name is ANKS4B. Also note that another IMAC component was recently discovered, named calmodulin-like protein 4 (CALML4) (Choi et al. JBC 2020).

We have corrected this error and included CALML4 in the introduction.

2) Fig 1F and Fig EV1E. It is unclear what "HPF" means in the Y-axis.

HPF mean high power field which is now explained in the corresponding Figure legends.

3) Fig 1I. You should point to an example of a fused microvillus for clarity.

We included an arrowhead pointing on fused microvilli in Fig 1I.

4) Page 9. "To date, no pathological consequences of IMAC dysfunction have been reported". Not sure if this is true. Patients with a deletion of their USH1C gene were described very early on to have severe enteropathy (Bitner-Glindzicz et al Nat Gen 2000). This correlates with the severe ultrastructural defects seen in the gut of USH1C KO mice (Crawley et al., Cell 2014).

Our statement referred to the interactions between protocadherins mediating microvillus cross-linking, which could be misleading. We have therefore made our statement more precise (we replaced "IMAC dysfunction" for "microvillus crosslinking defects" and included the literature on the patients with USH1C deletion in the discussion of the revised manuscript.

Referee #3:

The manuscript by Mödl and colleagues describes the phenotype of mice lacking CDHR5, a member of the protocadherin family that is involved in the formation of an adhesive complex at the tips of microvilli (intermicrovillar adhesive complex, IMAC). The authors find that CDHR5 depletion results in an altered organization and morphology (including reduced length, increased thickness and lower density) of microvilli, as well as in an altered localization of microvillar tip-associated proteins. The authors also observe an increased susceptibility to DSS-induced colitis. They find that this increase is not due to a breakdown on the tight junctions or due to an altered microbiome but is associated with increased apoptotic cell death and increased crypt cell proliferation. Consistent with enhanced susceptibility to DSS-induced colitis, bacterial infiltration of the mucosa is increased in CDHR5 knockout mice. Finally, using published data sets from human patients with ulcerative colitis the authors observe a downregulation of several IMAC components in enterocytes of inflamed tissues. In summary, the study provides evidence that CDHR5 is required for the ordered assembly of the brush border in intestinal epithelial cells and that its absence enhances colitis after DSS-induced epithelial damage. The study is largely well performed and includes the proper controls.

As a weak point of the study, a more detailed analysis of the localization of microvillar components is missing. The study shows only stainings of Ezrin and Ush1C (Fig. 2A), which, however, are of low quality with low resolution. Ezrin seems to be localized beneath Ush1C (Fig. 2A). Why? Shouldn't Ezrin be localized along the entire length of microvilli? And shouldn't P-Ezrin be localized at the tips to regulate the dynamic turnover of microvilli? Other microvillar tip markers such as CDHR2 and Myo7B are only displayed as line scans. Without immunofluorescence pictures at higher resolution and

magnification it is not possible to judge the quality of the stainings and specificity of the antibodies. The same applies for DPPIV stainings (Fig. 2E) and in additon for the IF analyses of IAP, NHE3, P-Ezrin (Fig. EV2). The authors do not provide any information on the selection of these molecules. Also, where is CDHR5 localized? Is it expressed (and localized in microvilli) only by cells lining the intestinal villi or is it also expressed (and localized in microvilli) by cells present in crypts where it could regulate proliferation? The authors provide some information from published databases which suggest a predominant expression in enterocytes and goblet cells but no expression in stem cells or TA cells. The authors should provide IF stainings for CDHR5 and the other microvillar components at high resolution to provide a better insight into the laterations observed after loss of CDHR5. As a further notion, in contrast to the increased bacterial invasion observed after depletion of CDHR5, the cell biological aspects of CDHR5 depletion are poorly discussed.

We fully agree with the reviewer and performed new IF staining and analysis for IMAC proteins with a spinning disc microscope (Olympus IXplore SpinSR). We included corresponding high magnification images for USH1C/Ezrin, CDHR2/Ezrin and MYO7B/Ezrin in the revised Fig 2A. Lower magnification images are now shown as Appendix Fig S4. Our stainings look quite similar to that of Pinette et al. (doi: 10.1091/mbc.E18-09-0558, Fig. 3) which used, however, Villin instead of Ezrin as marker for microvilli. We can only speculate that the Ezrin protein concentration gets lower at the microvillus tips. For P-Ezrin, Pinette et al. have also observed a similar distribution to Ezrin (doi: 10.1091/mbc.E18-09-0558, Fig. 6A) indicating that it is not exclusively localized to the microvillus tips. We also performed new profile plots that are now centered on the maximum Ezrin signal (Fig 2B) and provide a Spearman correlation coefficient for colocalization of IMAC components with Ezrin (Fig 2C). Furthermore, we included a correlation matrix for colocalization (Fig 2D). All these data show that IMAC components are not properly localized at the microvillus tips but rather localize like Ezrin along the whole microvillus.

We analyzed DPPIV, IAP, NHE3, P-Ezrin and Ezrin because these molecules are essential for intestinal function and were also analyzed by Pinette et al. (doi: 10.1091/mbc.E18-09-0558, Fig. 6) in the CDHR2 knockout mice. We rephrased the corresponding sentences in the Results section accordingly to make that clear. We included higher magnification laser scanning IF images of these proteins (Fig 2E) and detailed the method for quantitation of IF images in the Methods section of the Appendix and briefly in the legend of Fig 2F. We measured the amount of DPPIV, IAP, NHE3, P-Ezrin and Ezrin proteins with ImageJ using profile intensity of IF images. The area under the curve was calculated and used as measure for protein amount. We changed "relative intensity" for "AUC" in the corresponding scatter plots accordingly.

We detected protein expression of CDHR5 by IF mainly in differentiated intestinal epithelial cells whereas no fluorescent signals were evident in crypt cells. To illustrate this, we have replaced the low magnification images in Appendix Fig S1D with high magnification images. In addition, we performed a comprehensive analysis of CDHR5 expression in different intestinal cell using the single cell RNA-seq dataset of Haber et al. (doi: 10.1038/nature24489). The analysis showed that CDHR5 is mainly expressed in enterocytes (mature and immature) as well as in tuft cells, goblet cells and enteroendocrine cells. Expression is low in enterocyte progenitors and very weak in Paneth cells, stem cells and transit amplifying cells. We included these data in the revised manuscript as Fig EV1A.

Our discussion is already quite lengthy, also because additional text was required to discuss several points raised by the reviewers. We hope that the reviewer appreciates that we focused the discussion primarily on the pathological implications of impaired microvillus crosslinking.

Specific points:

1. The visualization of CDHR5 in WT, heterozygous and KO mice is very poor (Fig. S1D). The red fluorescence signals supposedly reflecting CDHR5 is still prominently present in Δ/Δ mice, albeit not at the apical membrane in the region depicted in the inset. The antibody does not seem to be specific. Also, the authors should provide images at higher magnification and resolution.

As mentioned above, we have replaced the low magnification images in Appendix Fig S1D with high magnification images. The CDHR5 antibody is highly specific. The original images of Appendix Fig S1D may have been overexposed for the red CDHR5 channel, giving the impression of an unspecific staining. This is now corrected in the revised Appendix Fig S1D.

2. The authors claim that colonic microvilli are disorganized and shortened and refer to Appendix Fig. S3B. This figure shows intestinal villi and does not provide information on microvilli organization.

Villi of the small intestine were shown in original Appendix Fig S3A. Original Appendix Fig S3B showed the surface of colonocytes with microvilli. To avoid confusion, we deleted Appendix Fig S3B, since high magnification SEM images of colonic microvilli are shown in Fig 1O, P of the revised manuscript. Original Appendix Fig S3A is now shown as Appendix Fig S3F.

3. The notion "normalization for brush border thickness abolished differences of apical marker expression in CDHR5+/+ and CDHR5-/- mice" (Results, paragraph 2) is unclear. Which difference is abolished? There is still apical localization of Ush1C (albeit not exclusive anymore). Similarly, the authors claim "the total amount of apical markers is reduced". Total amount means "total protein amount". Do the authors mean localization? How can they exclude that the "reduced amount of apical markers" is due to (or contributed to by) reduced protein expression? Did they perform Western blot experiments?

We apologize for the misleading formulations. With apical markers we meant DPPIV, IAP, NHE3, P-Ezrin and Ezrin but not IMAC proteins such as Ush1c. We made this clear in the revised manuscript. We have measured the amount of DPPIV, IAP, NHE3, P-Ezrin and Ezrin proteins with ImageJ using profile intensity of IF images. The area under the curve was calculated and used as measure for protein amount. We changed "relative intensity" for "AUC" in the corresponding scatter plots accordingly and described the method for quantitation in detail in the Methods section of the Appendix. Furthermore, the method is briefly described in the legend of Fig 2F. The quantitation resulted in reduced AUC in CDHR5$^{\Delta/\Delta}$ mice. However, we realized that the length of the fluorescent signals was also shortened in CDHR5$^{\Delta/\Delta}$ mice which is most likely due to the reduced microvillus length. When we normalized the AUC for the length of the fluorescent signals, the differences in AUC between CDHR5$^{+/+}$ and CDHR5$^{\Delta/\Delta}$ mice were abolished. We therefore argued that the overall protein expression of DPPIV, IAP, NHE3, P-Ezrin and Ezrin is not changed in CDHR5$^{\Delta/\Delta}$ mice, but the shortened microvilli provide less space for these proteins, which resulted in a reduced AUC. We made that point now clear in the revised manuscript. To strengthen our argument, we performed Western blot experiments for three apical markers (IAP, NHE3 and Ezrin) which are now included as Fig EV1G. The Western blot data proof that there is no reduced amount of overall IAP, NHE3 and Ezrin proteins in purified epithelial cells of CDHR5$^{\Delta/\Delta}$ mice.

4. All EM micrographs lack scale bars.

The original scale bars with additional information about EM settings are shown at the bottom of the SEM images. However, they are quite small for TEM images of Fig 1B, Fig 1I and Fig 1M of the revised manuscript. Therefore, we included separate scale bars for TEM images.

5. The Volcano plots shown in Fig. 2I lack sufficient labeling and/or explanation. What is the color code? The legend provides no information. The readers can only speculate.

We increased dot size, replaced gray with black colors to improve contrast and added a legend to the volcano plot (now shown as Fig 2G in the revised manuscript). We have also described the volcano plot in more detail in the legend of Fig 2G. In addition, quality control of the isolated intestinal epithelial cells and RNA used for the volcano plot is now provided in the revised manuscript as Appendix Fig S5.

6. The labeling to Fig. 2B-C is unclear. What is represented by the dotted line? Does it indicate the microvillar tip? What is the labeling of the X-axis, "tip distance (µm)" or "distance (µm)"? As pointed out above, the authors should provide representative IF images for each staining.

The dotted line indicated the microvillus tip in the original Fig 2B-D. As mentioned above, we provide new profile plots that are now centered on the maximum Ezrin signal (Fig 2B of the revised manuscript), which is explained in the Legend of Fig 2B.

7. For Fig. 2E, images of DPPIV with higher magnification are required to judge the intensity and localization of DPPIV.

We included higher magnification laser scanning IF images for all apical proteins (DPPIV, IAP, NHE3, P-Ezrin and Ezrin) in Fig 2E of the revised manuscript.

8. What is shown in Fig. EV2B, what is represented by the green fluorescence?

Original Fig EV2B showed high magnification IF images of the brush border stained for IAP. The images are intended to illustrate the method of measuring fluorescence signal length with the ZEN software and the reduced signal length in CDHR5$^{\Delta\Delta}$ mice. They are now shown in the revised manuscript as Fig EV1D and are described in detail in the Figure legend.

9. The terminology "thickness" and "intensity/thickness" for fluorescence signals is very uncommon. What do the authors means when saying DPPIV thickness, IAP thickness, NHE3 thickness,....? This is not scientific standard.

As state above, we changed "relative intensity" for "AUC" in the corresponding scatter plots and described the method for quantitation in detail in the methods section of the Appendix. We agree with the reviewer and have also replaced "thickness" and "intensity/thickness" for "signal length" and "AUC/signal length".

10. The notion "CDHR5 has mainly structural functions....but no major function in intracellular signaling" is a misconception. Intracellular signaling is not defined by changes in the levels of mRNAs ("gene expression").

We argued that altering intracellular signaling upon CDHR5 deletion would also result in a more substantial change in gene expression than observed by RNA-seq analysis. However, we did not analyze cell signaling pathways in CDHR5-deficient intestinal epithelial cells in more detail and therefore deleted this statement in the revised manuscript.

11. The Methods section is incomplete. For example, a section describing antibodies used for IF stainings is missing. Also, how were fluorescence signal intensities measured? How were line scans performed? A section describing RNA isolation and sequencing is also missing.

For reasons of space, methods such as immunofluorescence, in situ hybridization or RNA sequencing were described in the Appendix of the original submission. We have expanded our description about quantitation of fluorescence signals and how line scans were performed (see "Immunofluorescence" part of the revised Appendix). We have also included a paragraph on the methods used to quantify microvillus parameters under "Electron Microscopy" in the Methods section of the revised manuscript.

12. In some figures it is not clear if the significance values (*) apply to all data sets or only to the two "outer" data sets (e.g. Fig. 3C, K). In other figures, showing statistical significance values for all data points provides not useful information (e.g. Fig. 4C, E). As one example, it is not helpful to compare WT mice at day 0 with KO mice at day 3 or 7 (Fig. 4C, E, G). The authors should also indicate non-significant differences in the figures where applicable. Also, the violin plots shown in Fig. 5D display significance values in a selective manner. Why? For example, for CDHR2 the difference between healthy and non-inflamed tissue is indicated with three stars. What about the differences between healthy and inflamed or between inflamed and non-inflamed?

We only indicated significant differences. The bars above the data sets connect those data sets that differ significantly, which is commonly used for charts with more than two data sets. For example, in original Fig 3C, K, which contains three data sets, there is a significant difference between CDHR5$^{+/+}$ and CDHR5$^{\Delta/\Delta}$ mice, but not between CDHR5$^{+/+}$ or CDHR5$^{\Delta/\Delta}$ and heterozygous CDHR5$^{+/\Delta}$ mice. The same is true for original Fig 5D, which shows a significant difference between healthy and non-inflamed tissue for CDHR2, but not healthy and inflamed, or inflamed and non-inflamed. All statistical analysis of experiments with more than two datasets were performed with Anova and significant differences emerge from corresponding post hoc tests. Therefore, we also want to indicate all statistically significant differences in Fig 4C, E, which contains six datasets in total. We added vertical ticks in error bars above the datasets to make it clear which data sets were compared and to avoid further confusion.

13. In Fig. 3F bacteria (arrowheads) are not really visible for the reader at the given magnification (scale bar = 100 µm!).

We have shown a high magnification image of invading bacteria as an inset in Fig 4F. To better illustrate this, we have now also highlighted the region in the low magnification image used for the inset.

14. In Fig. 4G, it is not clear what the Y-axis depicts. The Y-axis legend reads "signals / cells (%)". Signals per how many cells?

We agree that this is confusing. We changed the Y-axis legend to "bacteria/intestinal cells (%)". A detailed description is now also included in the legend of Fig 4G.

15. The methods used for quantification are mentioned in the Methods section. However, it is not clear which method has been used in a given experiment. This information must be included in the figure legends.

Please see our response to point 11.

16. The notion "...the phenotypes observed in CDHR2-deficient mice...were phenocopied in CDHR5-deficient mice indicating that the protocadherins have overlapping function" (2nd paragraph in discussion) provides a poor discussion/interpretation of the observations. In fact, the two protocadherins have non-overlapping functions as they form a functional adhesive complex. Given that mouse CDHR5 can undergo homophilic interactions whereas mouse CDHR2 cannot (Gray et al (2022) PLoS Biol), it is clear that CDHR5 is a critical component of the IMAC to serve as binding partner for CDHR2 to mediate trans-interactions of microvilli.

We agree and have rephrased this paragraph accordingly in the discussion, based on the publication mentioned by the reviewer.

Minor issues

1. In the Introduction, Ezrin is mentioned to be a member of the ERM family as well as a member of the myosin family. This needs to be corrected.

We have mentioned members of the ERM and myosin families together. To avoid confusion, we moved the term Ezrin before MYO1A and MYO6.

1st Revision - Editorial Decision 20th Jul 2023

Dear Dr. Eferl,

Thank you for the submission of your revised manuscript to our editorial offices. I have already forwarded to you the reports I received from the three referees that I asked to re-evaluate your study, you will find again below. As you know, referee #2 now fully supports the publication of your manuscript in EMBO reports, whereas referees #1 and #3 have remaining concerns and suggestions to improve the manuscript.

After going through your preliminary point-by-points response (revision plan), I decided to proceed with the manuscript. Please revise the manuscript as indicated in your p-b-p-response, in particular by providing new with higher magnification (as indicated in the major point of referee #3). You might also show these in addition to the present images.

Please also provide a final detailed p-b-p-response addressing all the remaining referee comments.

Moreover, I have these editorial requests I ask you to address in a final revised manuscript:

- Please provide the abstract written in present tense throughout.

- We now use CRediT to specify the contributions of each author in the journal submission system. CRediT replaces the author contribution section. Please use the free text box to provide more detailed descriptions and remove the author contributions section from the manuscript text file. See also guide to authors:
https://www.embopress.org/page/journal/14693178/authorguide#authorshipguidelines

- Regarding data quantification and statistics, please make sure that the number "n" for how many independent experiments were performed, their nature (biological versus technical replicates), the bars and error bars (e.g. SEM, SD) and the test used to calculate p-values is indicated in the respective figure legends (main, EV figures and Appendix figures). Please also check that all the p-values are explained in the legend, and that these fit to those shown in the figure. Please provide statistical testing where applicable. Please avoid the phrase 'independent experiment', but clearly state if these were biological or technical replicates. Please also indicate (e.g. with n.s.) if testing was performed, but the differences are not significant. In case n=2 please show the data as separate datapoints or bars without error bars and statistics. See also:
http://www.embopress.org/page/journal/14693178/authorguide#statisticalanalysis

If n<5, please show single datapoints for diagrams.

- Please add scale bars of similar style and thickness to all the microscopic images (main, EV figures and Appendix figures), using clearly visible black or white bars (depending on the background). Please place these directly in the lower right corner of the images. Please do not write on or near the bars in the image but define the size in the respective figure legend. Some scale bars are presently rather thin. If possible, please remove the text present in some of the images.

- Please make sure that all figure panels are called out separately and sequentially (main, EV and Appendix figures). Presently, some panels of Figs. 1 and 3 and Appendix Fig S9 are not alphabetically/sequentially called out. Please change the text or the order of the panels in the figure. Moreover, there are no separate callouts for the panels of Appendix Fig S2A-J. Please check.

- Please remove the sections 'THE PAPER EXPLAINED' and 'SUPPORTING INFORMATION' from the manuscript text file.

- Please fuse the funding information with the Acknowledgements (or provide all funding information only in the acknowledgements section) and make sure that all the funding information is also entered into the online submission system and that it is complete and similar to the one in the acknowledgement section of the manuscript text file.

- Please order the manuscript sections like this, using these names:
Title page - Abstract - Keywords - Introduction - Results - Discussion - Materials and Methods - Data availability section - Acknowledgements - Disclosure and Competing Interests Statement - References - Figure legends - Expanded View Figure legends

- The data availability section is restricted to externally deposited datasets. Thus, please remove this part from this section: "Data and analytical methods have been included in the methods section. Materials will be made available to other researchers upon request under an appropriate material transfer agreement."

- Please move all methods information and references to the main manuscript text. We do not allow supplementary methods in the Appendix file.

- Please list the figures and tables separately in the Appendix TOC (table of contest), each with its page number.

- Please move the legends for each Appendix figure below the figure. I think this renders the Appendix file more comprehensible and easier to look at.

- It seems parts of the images shown in Figure 2A come from Appendix Figure S4 (showing full images) and parts of Appendix Figure S3B are also shown in Appendix figure S3D? Please confirm this and mention these reuses in the respective figure legends.

- At EMBO Press, we ask the authors to provide the source data that were used to generate the main figures. In the tow attached documents you will find a list of the figure panels for which we require source data and a FAQ file containing some useful information to help you prepare and submit the files. Please upload the source data file(s) when you submit your final revised version.

Please note that we do not expect you to provide us with "raw data" (e.g., qPCR Ct values), but with minimally processed data underlying the qualitative and quantitative data summarized in and used to generate the figures (e.g., mRNA increased expression folds), including replicates.

Data should be provided in a way that they could realistically be archived, shared, and possibly reused by other researchers for re-analysis purposes.

Please also note that upon acceptance for publication, source data will be linked to their respective figures. This will allow readers to access additional details from the experiments; they will also be stored at Biostudies (https://www.ebi.ac.uk/biostudies/) for long term archiving and they will get a unique accession number to make them citable in other studies.

- Finally, please find also attached a word file of the manuscript text (provided by our publisher) with changes we ask you to include in your final manuscript text and comments. Please use the attached file as basis for further revisions and provide your final manuscript file with track changes, in order that we can see any modifications done.

In addition, I would need from you:
- a short, two-sentence summary of the manuscript (not more than 35 words).
- two to four short bullet points highlighting the key findings of your study (two lines each).
- a schematic summary figure as separate file that provides a sketch of the major findings (not a data image) in jpeg or tiff format (with the exact width of 550 pixels and a height of not more than 400 pixels) that can be used as a visual synopsis on our website.

On a different note, I would like to alert you that EMBO Press offers a new format for a video-synopsis of work published with us, which essentially is a short, author-generated film explaining the core findings in hand drawings, and, as we believe, can be very useful to increase visibility of the work. Please see the following link for representative examples and their integration into the article web page:

https://www.embopress.org/video_synopses

https://www.embopress.org/doi/full/10.15252/embj.2019103932

Please let me know, should you be interested to engage in commissioning a similar video synopsis for your work. Operation instructions are available and intuitive.

I look forward to seeing the final revised version of your manuscript when it is ready. Please let me know if you have questions regarding the revision.

Yours sincerely,

Achim Breiling
Senior Editor
EMBO Reports

Referee #1:

Overall, the revised version of this manuscript shows significant improvements. Improvements include 1) the choice to focus the manuscript title and aim on whether microvillus cross-linking defects sensitize to murine colitis; 2) fuller characterization of the epithelial cells in the mutant mice; 3) the new IMAC/brush border IF microscopy images and analysis; 4) the addition of low-

power magnification views of colon histology; 5) several points within the text were refined or clarified.

A couple points remain that I feel were not adequately addressed.
1) I understand the point that the authors are trying to make for explaining why they observe no effect of genotype in the oxazolone and TNBS colitis models but do observe such an effect with the DSS model. However, I still feel that there is a missed opportunity to understand the mechanism behind the differences observed with these models in this study. The mechanism of DSS colitis is largely accepted to be that DSS is a chemical toxin that directly injures the colonic epithelial cells. As the authors point out, the T-cell mediated models are also targeting epithelial cells to induce an injury. So, are the mutant epithelial cells more sensitive to the DSS chemical, but they are not more sensitive to cytokine-induced injury? Is this an injury "dosage" situation? What is going on here?

2) It is disappointing that an objective measure of colitis severity was not included to support the subjective measures reported for the DSS colitis model.

Referee #2:

The authors have addressed my concerns/queries from their original manuscript submission.

Referee #3:

The authors provide a revised manuscript in which some concerns raised in my previous report have been addressed. One of my major points, however, remains. Higher resolution confocal images of the brush border are not provided. Although the authors claim to provide high magnification images of various IMAC components based on spinning disc microscopy, the new stainings shown in Fig. 2A show lower magnifications of BB components when compared with the original Fig.2A. So why do the authors claim to show "high magnification images"? Similarly, the authors claim that "Immunofluorescence and 3D image reconstruction of ZO-1, claudin-2 and claudin-5 proteins revealed no obvious tight junction defects", but the magnifications shown in App. Fig. S6 do allow for a reasonable judgement of the subcellular localization of these tight junction-localized proteins.

Additional points:

- Some statistical analyses make no sense (as one example, apoptotic rates of WT mice at day 0 are compared with Cdh5 KO mice at day 7; the same applies to several other quantifications in the same figure)
- The legend to Fig. EV1G is missing.
- Non-significant differences should be indicated by a "NS" where applicable.
- The authors claim: "loss of CDHR5 did not affect proliferation, apoptosis and differentiation of intestinal cell types (Appendix Fig. S2)". But: Apoptosis data are not shown in Appendix Fig. S2.

Response to reviewers

Referee #1:
Overall, the revised version of this manuscript shows significant improvements. Improvements include 1) the choice to focus the manuscript title and aim on whether microvillus cross-linking defects sensitize to murine colitis; 2) fuller characterization of the epithelial cells in the mutant mice; 3) the new IMAC/brush border IF microscopy images and analysis; 4) the addition of low-power magnification views of colon histology; 5) several points within the text were refined or clarified.
We thank the reviewer for valuable comments and recognition of our efforts to improve the manuscript.

A couple points remain that I feel were not adequately addressed.
1) I understand the point that the authors are trying to make for explaining why they observe no effect of genotype in the oxazolone and TNBS colitis models but do observe such an effect with the DSS model. However, I still feel that there is a missed opportunity to understand the mechanism behind the differences observed with these models in this study. The mechanism of DSS colitis is largely accepted to be that DSS is a chemical toxin that directly injures the colonic epithelial cells. As the authors point out, the T-cell mediated models are also targeting epithelial cells to induce an injury. So, are the mutant epithelial cells more sensitive to the DSS chemical, but they are not more sensitive to cytokine-induced injury? Is this an injury "dosage" situation? What is going on here?
We have addressed this point already in the discussion of the 1st revision. We do not fully agree that "the mechanism of DSS colitis is largely accepted to be that DSS is a chemical toxin that directly injures the colonic epithelial cells". There are other colitogenic effects of DSS and increased permeability of the mucus layer to bacteria is one of them. Our data suggest that this DSS effect but not epithelial injury/apoptosis (which occurred later than bacterial invasion) accounts for the difference in the severity of colitis between control and CDHR5-deficient mice which is explained in detail in our model (Fig. 6 of the 1st and the 2nd revision).

2) It is disappointing that an objective measure of colitis severity was not included to support the subjective measures reported for the DSS colitis model.
Which "objective measure of colitis severity" is meant? Cytokine levels are rarely shown in manuscripts, most likely because they are highly variable and inconsistent (at least in our experience, which we have already stated in our previous letter to reviewers). The same may be true for fecal bleeding or diarrhea. Most publications show weight loss, histology, colitis score (separate scores for immune infiltration, crypt damage and ulceration), and colon length to assess colitis severity (as we did).

Referee #3:
The authors provide a revised manuscript in which some concerns raised in my previous report have been addressed. One of my major points, however, remains. Higher resolution confocal images of the brush border are not provided. Although the authors claim to provide high magnification images of various IMAC components based on spinning disc microscopy, the new stainings shown in Fig. 2A show lower magnifications of BB components when compared with the original Fig.2A. So why do the authors claim to show "high magnification images"? Similarly, the authors claim that "Immunofluorescence and 3D image reconstruction of ZO-1, claudin-2 and claudin-5 proteins revealed no obvious tight junction defects", but the magnifications shown in App. Fig. S6 do allow for a reasonable judgement of the subcellular localization of these tight junction-localized proteins.

We apologize for the confusion, but we thought the reviewer was mostly unsatisfied with the resolution, not the magnification. We have significantly improved the resolution of the images using a spinning disk confocal microscope. Please compare Fig. 2A of the initial submission with that of our 1st revision:

Fig. 2A of initial manuscript:

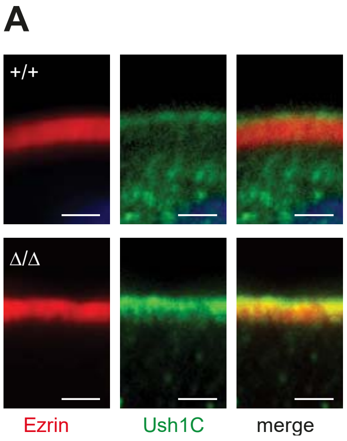

Fig. 2A of 1st revision:

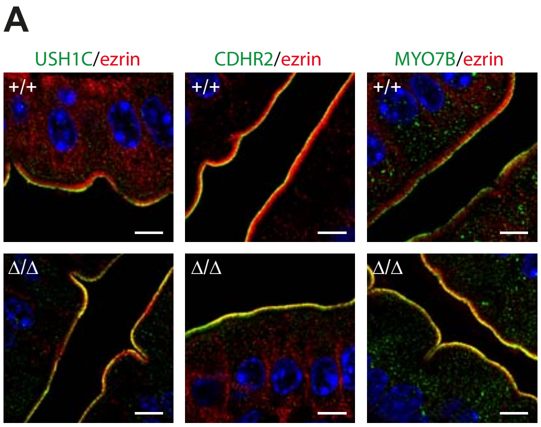

We cannot increase the resolution any further as there is no better microscope on the market. The images of the 1st revision are at high magnification (although not as high as in our initial manuscript) and show a clear difference. In order to emphasize the difference even more, we have replaced the images with very high magnification images in the 2nd revision.

Fig. 2A of 2nd revision:

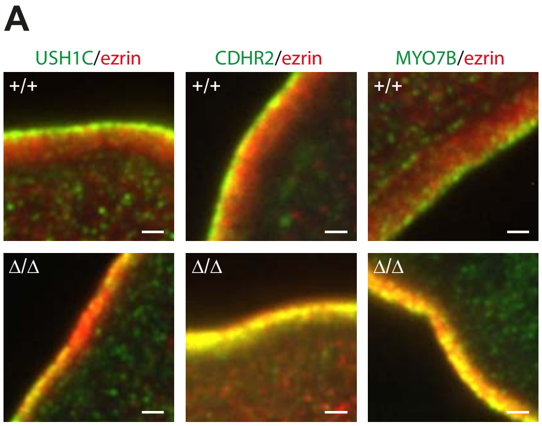

We have integrated the Fig. 2A images of the 1st revision into Appendix Fig. S4.
We now also show single confocal IF images of tight junction proteins in the 2nd revised Appendix Fig. S6A at high magnification and replaced the low magnification Z-stacks with high magnification Z-stacks in Appendix Fig. S6B.

Additional points:
1) Some statistical analyses make no sense (as one example, apoptotic rates of WT mice at day 0 are compared with Cdh5 KO mice at day 7; the same applies to several other quantifications in the same figure)
We don't agree on the statistics. We have shown all significant differences as the statistical analysis was performed using Anova (we have three time points) and a corresponding post hoc test. Some readers may be interested in whether there is a statistical difference between days 0, 3 and 7.

2) The legend to Fig. EV1G is missing.
We apologize for the mistake and have included a legend.

3) Non-significant differences should be indicated by a "NS" where applicable.
Additionally, showing all non-significant differences with NS in the graphs analyzed by Anova would make them quite confusing. Most graphs in scientific manuscripts do not indicate non-significant differences with NS. We could use separate t-tests for days 0, 3 and 7 in Fig. 4C, D and show non-significant differences with NS, but this is not a suitable statistical test for this type of data.

4) The authors claim: "loss of CDHR5 did not affect proliferation, apoptosis and differentiation of intestinal cell types (Appendix Fig. S2)". But: Apoptosis data are not shown in Appendix Fig. S2.
We have shown apoptosis data of the colon in Fig. 4B, C. Corresponding data of the small intestine are now included in the 2nd revised Appendix Fig. S2.

2nd Revision - Editorial Decision 22nd Aug 2023

Robert Eferl
Medical University of Vienna
Center for Cancer Research
Borschkegasse 8a
Vienna, Vienna 1090
Austria

Dear Dr. Eferl,

Thank you for the submission of your further revised manuscript to our editorial offices. I have now received the report from the referee that I asked to re-assess the study, you will find below. As you will see, the referee now fully supports the publication of your study in EMBO reports.

I am thus very pleased to accept your manuscript for publication in the next available issue of EMBO reports. Thank you for your contribution to our journal.

At the end of this email I include important information about how to proceed. Please ensure that you take the time to read the information and complete and return the necessary forms to allow us to publish your manuscript as quickly as possible.

As part of the EMBO publication's Transparent Editorial Process, EMBO reports publishes online a Review Process File to accompany accepted manuscripts. As you are aware, this File will be published in conjunction with your paper and will include the referee reports, your point-by-point response and all pertinent correspondence relating to the manuscript.

If you do NOT want this File to be published, please inform the editorial office within 2 days, if you have not done so already, otherwise the File will be published by default [contact: emboreports@embo.org]. If you do opt out, the Review Process File link will point to the following statement: "No Review Process File is available with this article, as the authors have chosen not to make the review process public in this case."

Thank you again for your contribution to EMBO reports and congratulations on a successful publication. Please consider us again in the future for your most exciting work.

Yours sincerely,

Achim Breiling
Senior Editor
EMBO Reports

Referee #3:

The authors provide a revised manuscript in which my remaining issues concerning the microscopic images of the brush border and of intestinal epithelial cells have been adequately addressed.

****

THINGS TO DO NOW:

Please note that you will be contacted by Wiley Author Services to complete licensing and payment information. The required 'Page Charges Authorization Form' is available here: https://www.embopress.org/pb-assets/embo-site/er_apc.pdf - please download and complete the form and return to embopressproduction@wiley.com

EMBO Press participates in many Publish and Read agreements that allow authors to publish Open Access with reduced/no publication charges. Check your eligibility: https://authorservices.wiley.com/author-resources/Journal-Authors/open-access/affiliation-policies-payments/index.html

You will receive proofs by e-mail approximately 2-3 weeks after all relevant files have been sent to our Production Office; you should return your corrections within 2 days of receiving the proofs.

Please inform us if there is likely to be any difficulty in reaching you at the above address at that time. Failure to meet our

deadlines may result in a delay of publication, or publication without your corrections.

All further communications concerning your paper should quote reference number EMBOR-2023-57084V3 and be addressed to emboreports@wiley.com.

Should you be planning a Press Release on your article, please get in contact with emboreports@wiley.com as early as possible, in order to coordinate publication and release dates.

EMBO Press Author Checklist

Corresponding Author Name: Robert Eferl	
Journal Submitted to: EMBO Reports	
Manuscript Number: EMBOR-2023-57084V2-Q	

USEFUL LINKS FOR COMPLETING THIS FORM
The EMBO Journal - Author Guidelines
EMBO Reports - Author Guidelines
Molecular Systems Biology - Author Guidelines
EMBO Molecular Medicine - Author Guidelines

Reporting Checklist for Life Science Articles (updated January
This checklist is adapted from Materials Design Analysis Reporting (MDAR) Checklist for Authors. MDAR establishes a minimum set of requirements in transparent reporting in the life sciences (see Statement of Task: 10.31222/osf.io/9sm4x). Please follow the journal's guidelines in preparing your manuscript.
Please note that a copy of this checklist will be published alongside your article.

Abridged guidelines for figures
1. Data
The data shown in figures should satisfy the following conditions:
- the data were obtained and processed according to the field's best practice and are presented to reflect the results of the experiments in an accurate and unbiased manner.
- ideally, figure panels should include only measurements that are directly comparable to each other and obtained with the same assay.
- plots include clearly labeled error bars for independent experiments and sample sizes. Unless justified, error bars should not be shown for technical
- if n<5, the individual data points from each experiment should be plotted. Any statistical test employed should be justified.
- Source Data should be included to report the data underlying figures according to the guidelines set out in the authorship guidelines on Data

2. Captions
Each figure caption should contain the following information, for each panel where they are relevant:
- a specification of the experimental system investigated (eg cell line, species name).
- the assay(s) and method(s) used to carry out the reported observations and measurements.
- an explicit mention of the biological and chemical entity(ies) that are being measured.
- an explicit mention of the biological and chemical entity(ies) that are altered/varied/perturbed in a controlled manner.
- the exact sample size (n) for each experimental group/condition, given as a number, not a range;
- a description of the sample collection allowing the reader to understand whether the samples represent technical or biological replicates (including how many animals, litters, cultures, etc.).
- a statement of how many times the experiment shown was independently replicated in the laboratory.
- definitions of statistical methods and measures:
  - common tests, such as t-test (please specify whether paired vs. unpaired), simple χ2 tests, Wilcoxon and Mann-Whitney tests, can be unambiguously identified by name only, but more complex techniques should be described in the methods section;
  - are tests one-sided or two-sided?
  - are there adjustments for multiple comparisons?
  - exact statistical test results, e.g., P values = x but not P values < x;
  - definition of 'center values' as median or average;
  - definition of error bars as s.d. or s.e.m.

Please complete ALL of the questions below.
Select "Not Applicable" only when the requested information is not relevant for your study.

Materials

Newly Created Materials	Information included in the manuscript?	In which section is the information available? (Reagents and Tools Table, Materials and Methods, Figures, Data Availability Section)
New materials and reagents need to be available; do any restrictions apply?	Not Applicable	

Antibodies	Information included in the manuscript?	In which section is the information available? (Reagents and Tools Table, Materials and Methods, Figures, Data Availability Section)
For **antibodies** provide the following information:<br>- Commercial antibodies: RRID (if possible) or supplier name, catalogue number and or/clone number<br>- Non-commercial: RRID or citation	Yes	

DNA and RNA sequences	Information included in the manuscript?	In which section is the information available? (Reagents and Tools Table, Materials and Methods, Figures, Data Availability Section)
**Short novel DNA or RNA including primers, probes**: provide the sequences.	Yes	

Cell materials	Information included in the manuscript?	In which section is the information available? (Reagents and Tools Table, Materials and Methods, Figures, Data Availability Section)
**Cell lines:** Provide species information, strain. Provide accession number in repository **OR** supplier name, catalog number, clone number, and/**OR** RRID.	Not Applicable	
**Primary cultures:** Provide species, strain, sex of origin, genetic modification status.	Not Applicable	
Report if the cell lines were recently **authenticated** (e.g., by STR profiling) and tested for mycoplasma contamination.	Not Applicable	

Experimental animals	Information included in the manuscript?	In which section is the information available? (Reagents and Tools Table, Materials and Methods, Figures, Data Availability Section)
**Laboratory animals or Model organisms:** Provide species, strain, sex, age, genetic modification status. Provide accession number in repository **OR** supplier name, catalog number, clone number, **OR** RRID.	Yes	
**Animal observed in or captured from the field:** Provide species, sex, and age where possible.	Not Applicable	
Please detail **housing and husbandry conditions**.	Yes	

Plants and microbes	Information included in the manuscript?	In which section is the information available? (Reagents and Tools Table, Materials and Methods, Figures, Data Availability Section)
**Plants:** provide species and strain, ecotype and cultivar where relevant, unique accession number if available, and source (including location for collected wild specimens).	Not Applicable	
**Microbes:** provide species and strain, unique accession number if available, and source.	Not Applicable	

Human research participants	Information included in the manuscript?	In which section is the information available? (Reagents and Tools Table, Materials and Methods, Figures, Data Availability Section)
If collected and within the bounds of privacy constraints report on age, sex and gender or ethnicity for all study participants.	Not Applicable	

Core facilities	Information included in the manuscript?	In which section is the information available? (Reagents and Tools Table, Materials and Methods, Figures, Data Availability Section)
If your work benefited from core facilities, was their service mentioned in the acknowledgments section?	Yes	

Design

Study protocol	Information included in the manuscript?	In which section is the information available? (Reagents and Tools Table, Materials and Methods, Figures, Data Availability Section)
If study protocol has been **pre-registered, provide DOI in the manuscript**. For clinical trials, provide the trial registration number **OR** cite DOI.	Not Applicable	
Report the **clinical trial registration number** (at ClinicalTrials.gov or equivalent), where applicable.	Not Applicable	

Laboratory protocol	Information included in the manuscript?	In which section is the information available? (Reagents and Tools Table, Materials and Methods, Figures, Data Availability Section)
Provide DOI OR other citation details if **external detailed step-by-step protocols** are available.	Yes	

Experimental study design and statistics	Information included in the manuscript?	In which section is the information available? (Reagents and Tools Table, Materials and Methods, Figures, Data Availability Section)
Include a statement about **sample size** estimate even if no statistical methods were used.	Yes	
Were any steps taken to minimize the effects of subjective bias when allocating animals/samples to treatment (e.g. **randomization procedure**)? If yes, have they been described?	Yes	
Include a statement about **blinding** even if no blinding was done.	Yes	
Describe **inclusion/exclusion criteria** if samples or animals were excluded from the analysis. Were the criteria pre-established?<br><br>If sample or data points were omitted from analysis, report if this was due to attrition or intentional exclusion and provide justification.	Yes	
For every figure, are **statistical tests** justified as appropriate? Do the data meet the assumptions of the tests (e.g., normal distribution)? Describe any methods used to assess it. Is there an estimate of variation within each group of data? Is the variance similar between the groups that are being statistically compared?	Yes	

Sample definition and in-laboratory replication	Information included in the manuscript?	In which section is the information available? (Reagents and Tools Table, Materials and Methods, Figures, Data Availability Section)
In the figure legends: state number of times the experiment was **replicated** in laboratory.	Yes	
In the figure legends: define whether data describe **technical or biological replicates**.	Yes	

Ethics

Ethics	Information included in the manuscript?	In which section is the information available? (Reagents and Tools Table, Materials and Methods, Figures, Data Availability Section)
Studies involving **human participants**: State details of **authority granting ethics approval** (IRB or equivalent committee(s), provide reference number for approval.	Not Applicable	
Studies involving **human participants**: Include a statement confirming that **informed consent** was obtained from all subjects and that the experiments conformed to the principles set out in the WMA Declaration of Helsinki and the Department of Health and Human Services Belmont Report.	Not Applicable	
Studies involving **human participants:** For publication of **patient photos**, include a statement confirming that consent to publish was obtained.	Not Applicable	
Studies involving experimental **animals**: State details of **authority granting ethics approva**l (IRB or equivalent committee(s), provide reference number for approval. Include a statement of compliance with ethical regulations.	Yes	
Studies involving **specimen and field samples:** State if relevant **permits** obtained, provide details of authority approving study; if none were required, explain why.	Not Applicable	

Dual Use Research of Concern (DURC)	Information included in the manuscript?	In which section is the information available? (Reagents and Tools Table, Materials and Methods, Figures, Data Availability Section)
Could your study fall under dual use research restrictions? Please check biosecurity documents and list of **select agents and toxins** (CDC): https://www.selectagents.gov/sat/list.htm	Not Applicable	
If you used a select agent, is the security level of the lab appropriate and reported in the manuscript?	Not Applicable	
If a study is subject to dual use research of concern regulations, is the name of the **authority granting approval and reference number** for the regulatory approval provided in the manuscript?	Not Applicable	

Reporting

The MDAR framework recommends adoption of discipline-specific guidelines, established and endorsed through community initiatives. Journals have their own policy about requiring specific guidelines and recommendations to complement MDAR.

Adherence to community standards	Information included in the manuscript?	In which section is the information available? (Reagents and Tools Table, Materials and Methods, Figures, Data Availability Section)
State if relevant guidelines or checklists (e.g., **ICMJE, MIBBI, ARRIVE, PRISMA**) have been followed or provided.	Not Applicable	
For **tumor marker prognostic studies,** we recommend that you follow the **REMARK** reporting guidelines (see link list at top right). See author guidelines, under 'Reporting Guidelines'. Please confirm you have followed these guidelines.	Not Applicable	
For **phase II and III randomized controlled trials**, please refer to the **CONSORT** flow diagram (see link list at top right) and submit the CONSORT checklist (see link list at top right) with your submission. See author guidelines, under 'Reporting Guidelines'. Please confirm you have submitted this list.	Not Applicable	

Data Availability

Data availability	Information included in the manuscript?	In which section is the information available? (Reagents and Tools Table, Materials and Methods, Figures, Data Availability Section)
Have **primary datasets** been deposited according to the journal's guidelines (see 'Data Deposition' section) and the respective accession numbers provided in the Data Availability Section?	Yes	
Were **human clinical and genomic datasets** deposited in a public access-controlled repository in accordance to ethical obligations to the patients and to the applicable consent agreement?	Not Applicable	
Are **computational models** that are central and integral to a study available without restrictions in a machine-readable form? Were the relevant accession numbers or links provided?	Not Applicable	
If publicly available data were reused, provide the respective **data citations in the reference list**.	Yes	
